# Supplementary material for: TRIM2 inhibits apoptosis by ubiquitinating BNIP3 to protect the intestine against ischemia-reperfusion injury in mice
Source: Commun Biol. 2025 Aug 29;8:1308. doi: 10.1038/s42003-025-08708-2 (PMC12397258; doi:10.1038/s42003-025-08708-2)

TRIM2 inhibits apoptosis by ubiquitinating BNIP3 to protect the intestine against ischemia-reperfusion injury in mice

Jinping Nie[1†], Chao Mei[1,2†], Aiping Wei[1], Yingjie Wang[1], Chenlu Fan[1], Yingjie Huang[1], Ming Jiang[1], Han Che[1], Tao Chen[1], Juan Tian[1], Yong Li[1]*, Xuan Huang[3]*, Xuekang Zhang[1]*

[1]Department of Anesthesiology, The First Affiliated Hospital, Jiangxi Medical College, Nanchang University, Nanchang 330006, China;

[2]Department of Surgery and Anesthesia, Ganjiang New Area Hospital of The First Affiliated Hospital of Nanchang University, Ganjiang New Area People's Hospital, Nanchang 330029, China

[3]The National Engineering Research Center for Bioengineering Drugs and the Technologies, Jiangxi Provincial Key Laboratory of Bioengineering Drugs, Institute of Translational Medicine, Jiangxi Medical College, Nanchang University, Nanchang 330031, PR China;

[†]These authors contributed equally

***Correspondence:**

Yong Li, Phone: +86-13979131536; Email: liyong@ncu.edu.cn;

Xuan Huang, Phone: +86-13807058627; Email: huangxuan@ncu.edu.cn.

Xuekang Zhang, Phone: +86-13870639612; Email: ndyfy00768@ncu.edu.cn;

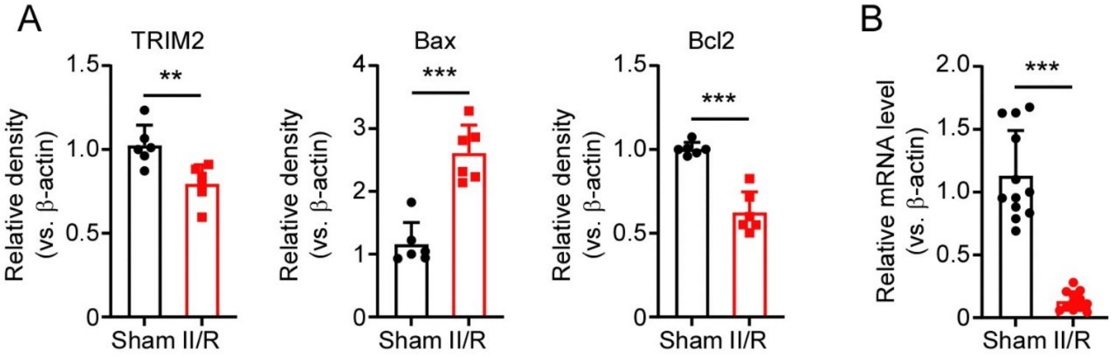

Supplementary Figure 1. The expression of TRIM2 is down-regulated in II/R mice model

(A) The quantification of the Western blot bands shown in Figure 1I was performed using the Gel-Pro Analyzer software. (B) TRIM2 mRNA levels in the intestine of WT mice subjected to either a sham operation or II/R was investigated. All data are presented as the mean ± SD. The levels of statistical significance are indicated as follows: ** $p < 0.01$, *** $p < 0.001$ by Student's t-test.

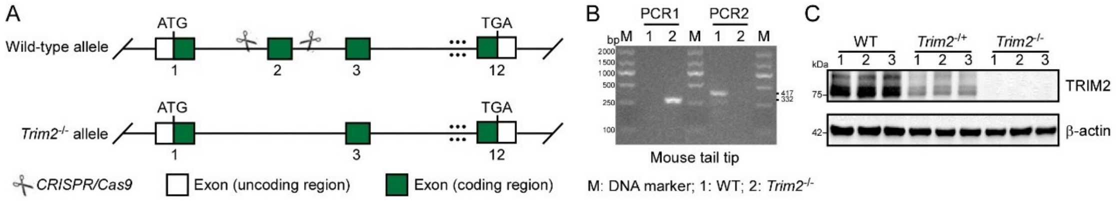

Supplementary Figure 2. Construction and identification of *Trim2*$^{-/-}$ mice

(A) Diagram of *Trim2*$^{-/-}$ mice strategy (B) The tails of WT and *Trim2*$^{-/-}$ mice were clipped by 2 mm to extract DNA. The extracted DNA was amplified by PCR using specific primers, and DNA electrophoresis was conducted to identify the mice genotypes. (C) Western blot analysis was performed to test the TRIM2 protein expression in the intestine of WT, *Trim2*$^{-/+}$, and *Trim2*$^{-/-}$ mice.

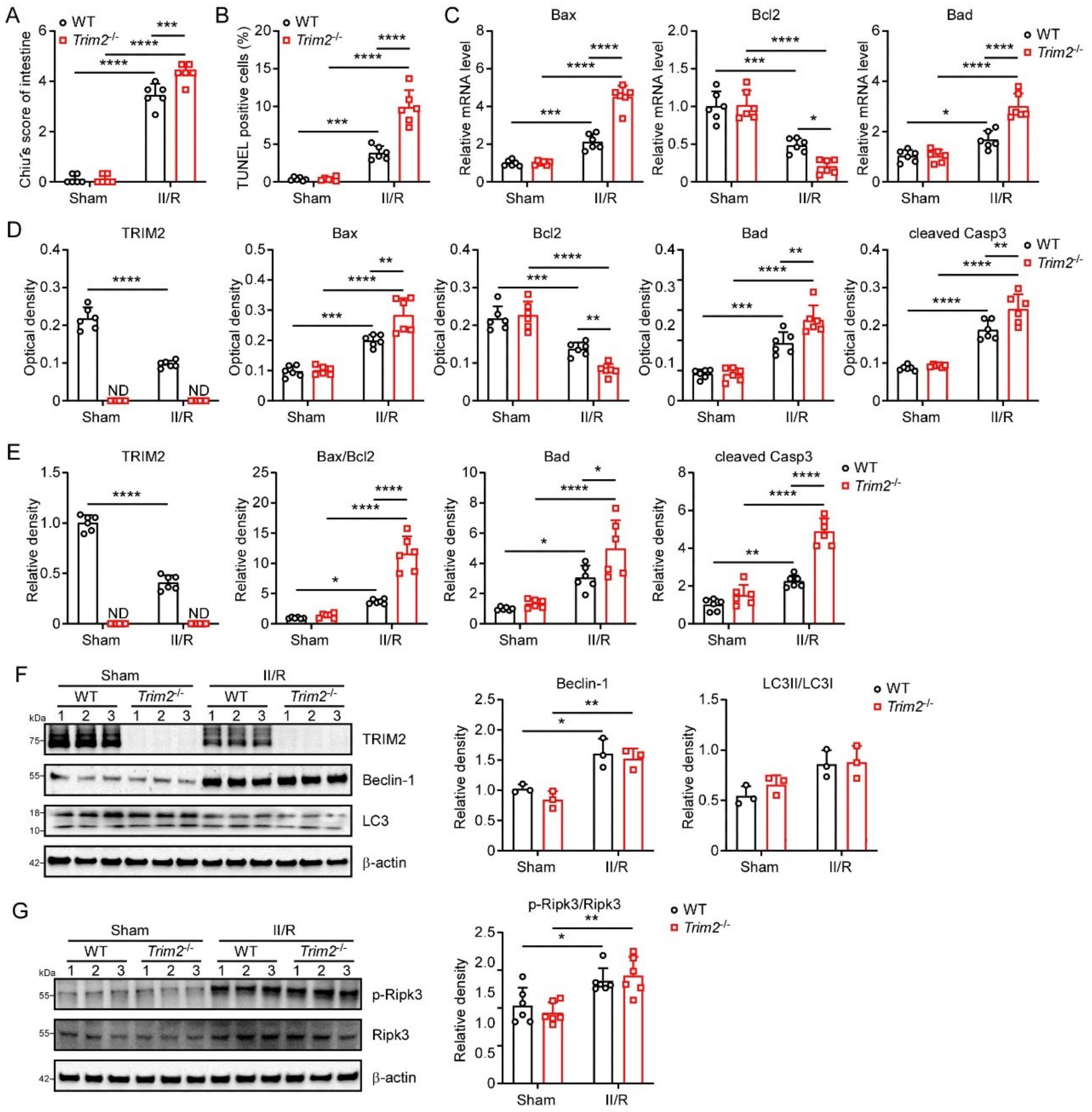

Supplementary Figure 3. The knockout of TRIM2 promotes II/R-induced apoptosis in mice

(A) The histopathological injury scores of the intestine were quantified using the criteria of Chiu's score. (B) The percentage of TUNEL-positive cells in the WT-sham, *Trim2*$^{-/-}$-sham, WT-II/R, and *Trim2*$^{-/-}$-II/R groups shown in Figure 2B was determined using ImageJ software. (C) The relative mRNA levels of Bax, Bcl-2, and Bad in the intestine were determined by qRT-PCR. (D) The optical intensities of TRIM2, Bax, Bcl-2, Bad, and cleaved Casp3 IHC staining, as illustrated in Figure 2C, were quantified using ImageJ software. (E) The quantification of the Western blot bands presented in Figure 2D was conducted using the ImageJ software. (F) The expression levels of autophagy-related proteins, Beclin-1 and LC3, were determined through Western blot analysis and subsequently quantified in grayscale using the ImageJ software. (G) The expression levels of necroptosis-related proteins, p-Ripk3 and Ripk3, were determined through Western blot analysis and subsequently quantified in grayscale using the ImageJ software. All results are expressed as the mean ± SD. The statistical significance was determined using one-way ANOVA followed by Tukey's test, with the following levels of significance: * $p < 0.05$, ** $p < 0.01$, *** $p < 0.001$, and **** $p < 0.0001$.

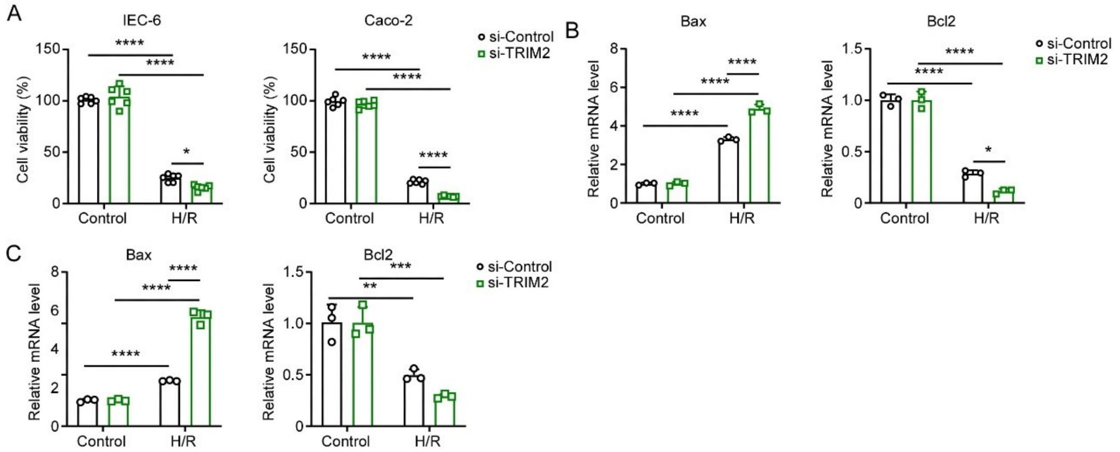

Supplementary Figure 4. TRIM2 knockdown exacerbates H/R-induced apoptosis in vitro

(A) CCK8 experiment was conducted to detect the cell viability of IEC-6 and Caco-2 cells. (B) The relative mRNA levels of Bax and Bcl-2 in IEC-6 cells with TRIM2 knockdown that were subjected to either a control or H/R treatment. (C) Relative mRNA levels of Bax and Bcl-2 in TRIM2-knockdown Caco-2 cells subjected to control or H/R treatment. All results are expressed as the mean ± SD. Statistical significance was determined using one-way ANOVA followed by Tukey's test, with the following levels of significance: * $p < 0.05$, ** $p < 0.01$, *** $p < 0.001$, **** $p < 0.0001$.

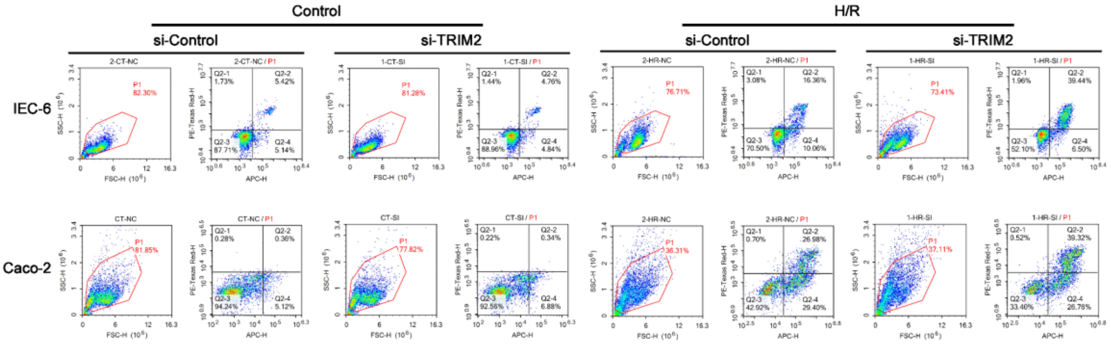

Supplementary Figure 5. Flow cytometric analysis of apoptosis in TRIM2 knockdown cells.

Representative example of gating strategy in apoptosis, using co-staining with Annexin-V and PI. Cells in the lower right quadrant (Annexin V^{+}/PI^{-}) represent early apoptosis; cells in the upper right quadrant (Annexin V^{+}/PI^{+}) represent late apoptosis.

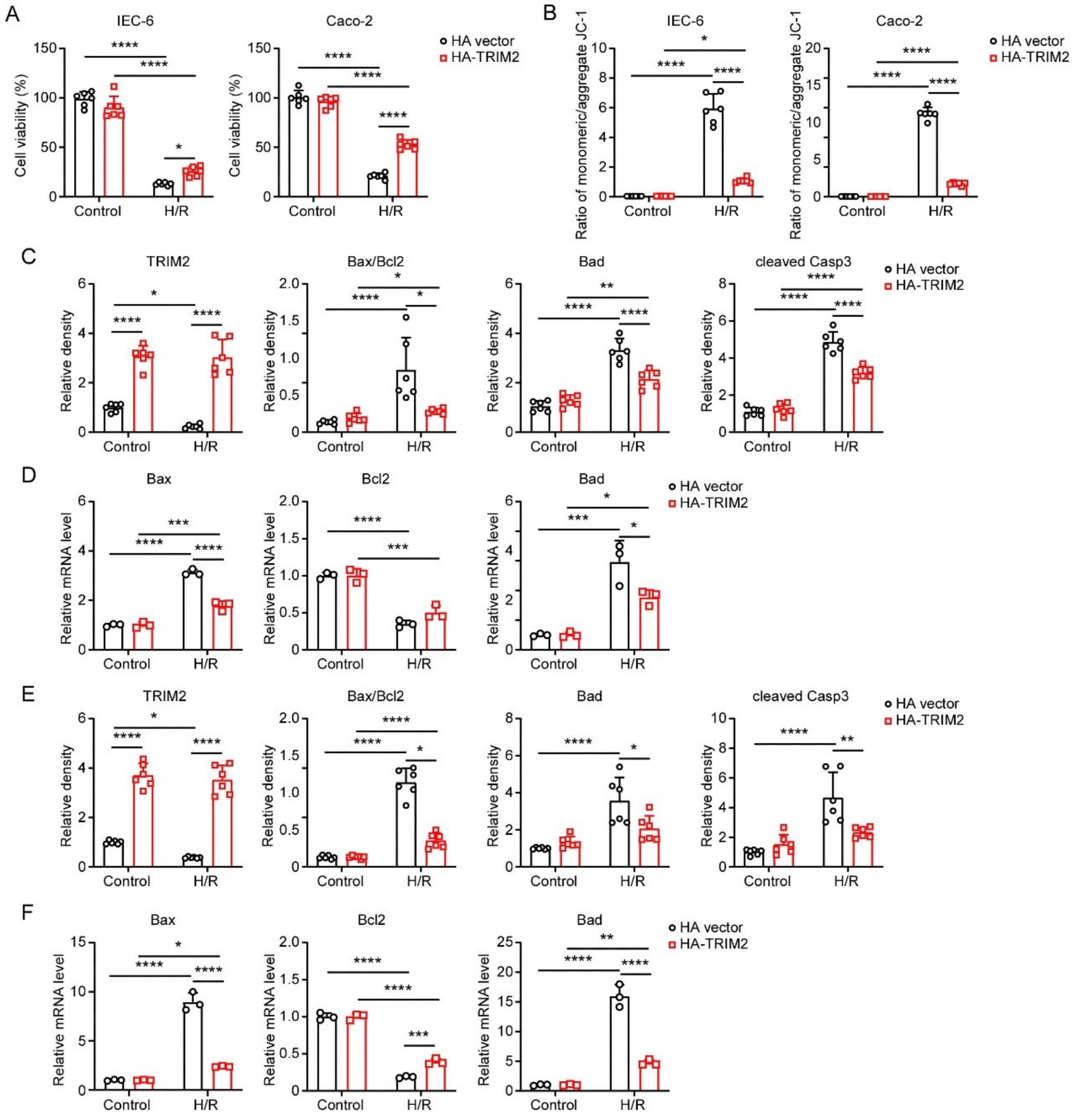

Supplementary Figure 6. The elevation of TRIM2 mitigates H/R-induced apoptosis in vitro. (A) A CCK8 experiment was conducted to ascertain the viability of IEC-6 and Caco-2 cells in the Control+HA-vector, Control+HA-TRIM2, H/R+HA-vector, and H/R+HA-TRIM2 groups. (B) Ratios of monomeric/aggregate JC-1 in IEC-6 and Caco-2 cells, as illustrated in Figure 4B, were determined using the ImageJ software. (C) The quantification of the western blot bands in IEC-6 cells, as illustrated in Figure 4C, was conducted using the Gel-Pro Analyzer software. (D) Relative mRNA expression levels of Bax, Bcl-2, and Bad in TRIM2-overexpressing IEC-6 cells under Control or H/R conditions. (E) Quantification of the western blot bands in Caco-2 cells shown in Figure 4C was performed using Gel-Pro Analyzer software. (F) Relative mRNA expression levels of Bax, Bcl-2, and Bad in TRIM2-overexpressing CACO-2 cells under Control or H/R conditions. All results are expressed as the mean ± SD. Statistical significance was determined using one-way ANOVA followed by Tukey's test, with the following levels of significance: * $p < 0.05$, ** $p < 0.01$, *** $p < 0.001$, **** $p < 0.0001$.

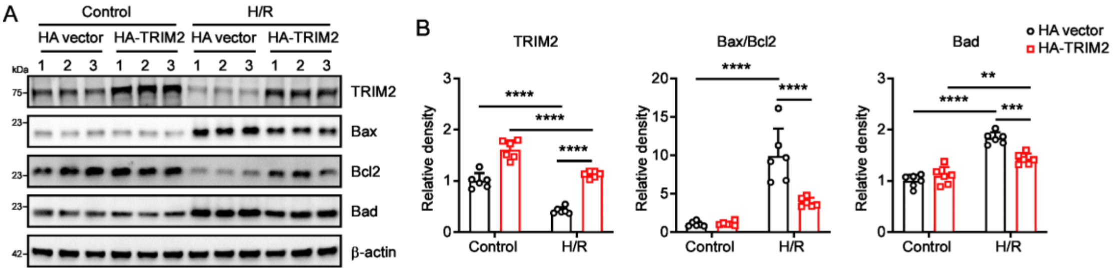

Supplementary Figure 7. Overexpression of TRIM2 promotes H/R-induced apoptosis in IECs. (A) Western blot analysis of the effect of overexpression of HA-TRIM2 in IECs on apoptosis related proteins Bax, Bcl-2, and Bad. (B) The quantification of the Western blot bands was conducted using the ImageJ software. All results are expressed as the mean ± SD. Statistical significance was determined using one-way ANOVA followed by Tukey's test, with the following levels of significance: ** $p < 0.01$, *** $p < 0.001$, **** $p < 0.0001$.

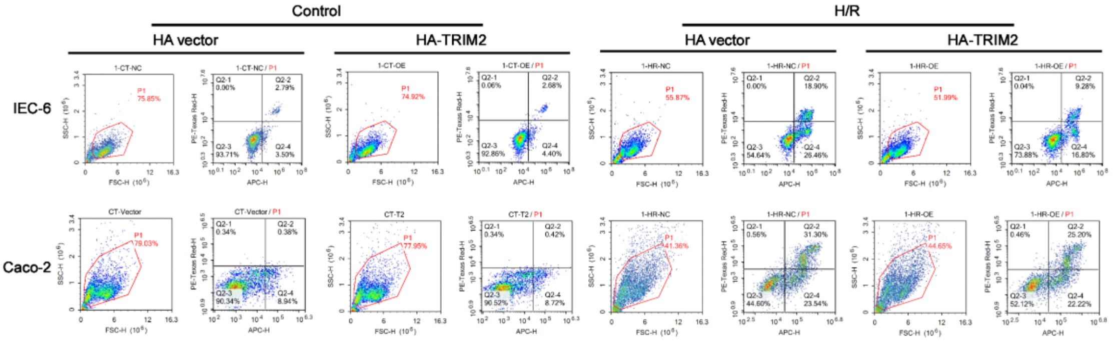

Supplementary Figure 8. Flow cytometric analysis of apoptosis in TRIM2-overexpressing cells. Representative example of gating strategy in apoptosis, using co-staining with Annexin-V and PI. Cells in the lower right quadrant (Annexin V^+/PI^-) represent early apoptosis; cells in the upper right quadrant (Annexin V^+/PI^+) represent late apoptosis.

A

Gene	Position	Gly (K) Probabilities	Lenti-control/Lenti-Trim2	*p*-value
BNIP3	130	ENIPPK(1)EFLF	8.124	0.00081
BNIP3	111	EVESILK(0.908)K(0.092)	0.478	0.29458
BNIP3	153	K(1)GGIFSAEFLK	1.676	0.54906

B

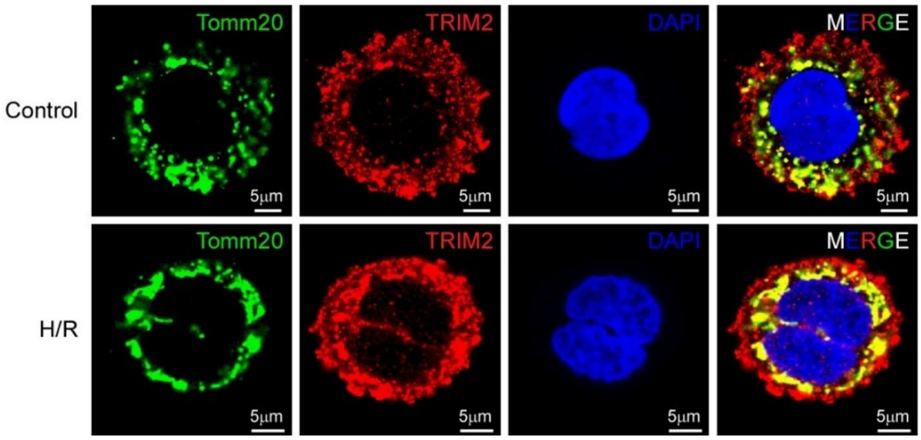

Supplementary Figure 9. TRIM2 partially localizes to mitochondria and mediates ubiquitination of BNIP3

(A) Quantitative ubiquitination proteomics screening revealed that the K130 site of BNIP3 may be a ubiquitination site mediated by TRIM2. (B) Representative confocal images of Caco-2 cells demonstrated the co-localization and distribution of TRIM2 and Tomm20.

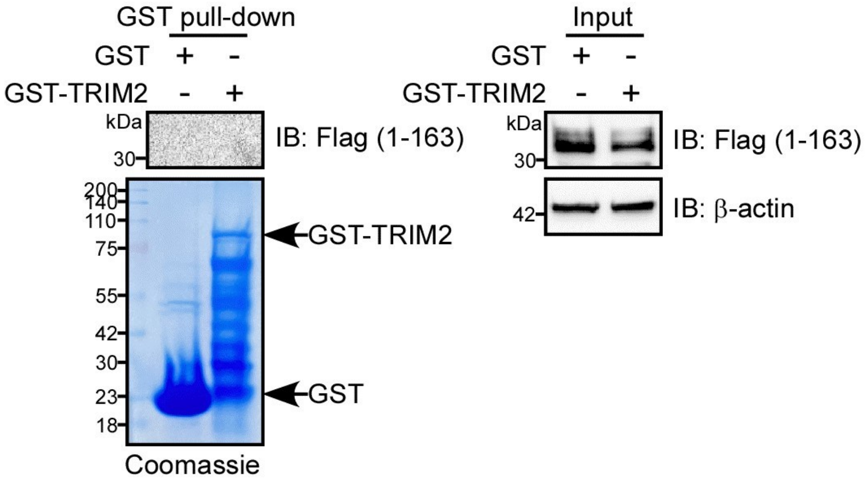

Supplementary Figure 10. The TRIM2 protein does not bind to the N-terminus of the BNIP3 protein

Flag-tagged N-terminus of BNIP3 (1-163) plasmids were transfected into HEK293T cells, and collecting lysates from HEK293T cells after 24 hours of transfection. Purified GST or GST-TRIM2 proteins incubated with the collected lysates overnight. The direct interaction between TRIM2 and the N-terminus of BNIP3 (1-163) was verified by GST-pulldown and western blot experiment, and purity of GST-TRIM2 was assessed through Coomassie blue staining.

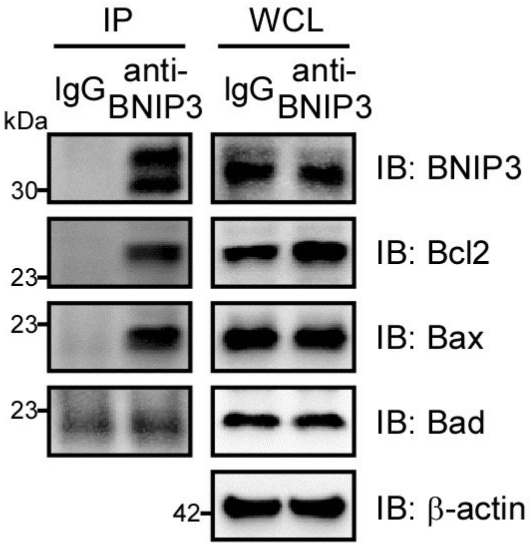

Supplementary Figure 11. TRIM2 can bind to Bax and Bcl-2, but cannot bind to Bad in Caco-2 cells

Using BNIP3 specific antibodies for immunoprecipitation experiments, IgG was used as a negative control to detect the interaction between BNIP3 and apoptosis related proteins Bax, Bcl-2, and Bad in Caco-2 cells.

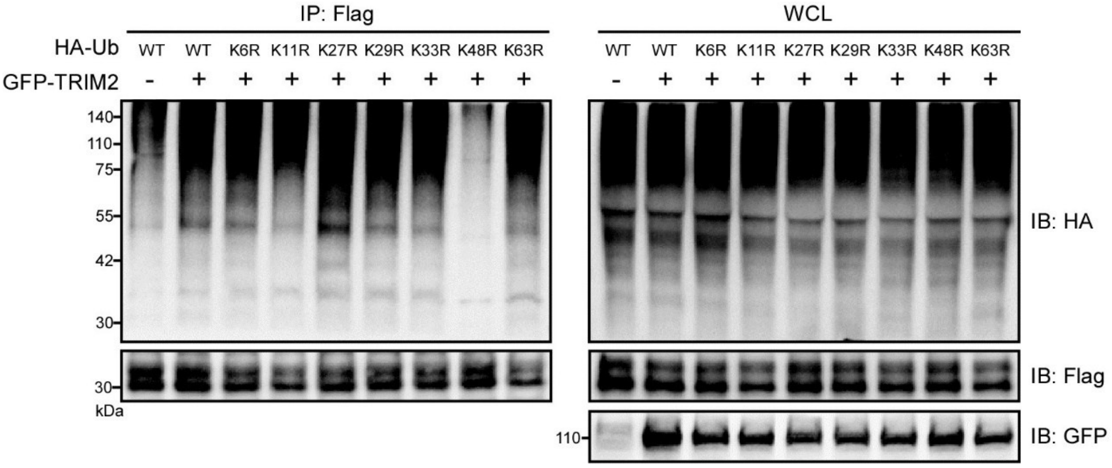

Supplementary Figure 12. TRIM2 catalyzes K48-linked polyubiquitination of BNIP3

The indicated plasmids were cotransfected into HEK293T cells for 24 hours, the absence and level of ubiquitination of BNIP3 was detected by co-immunoprecipitation with anti-Flag tag antibodies, and IB with the indicated antibodies.

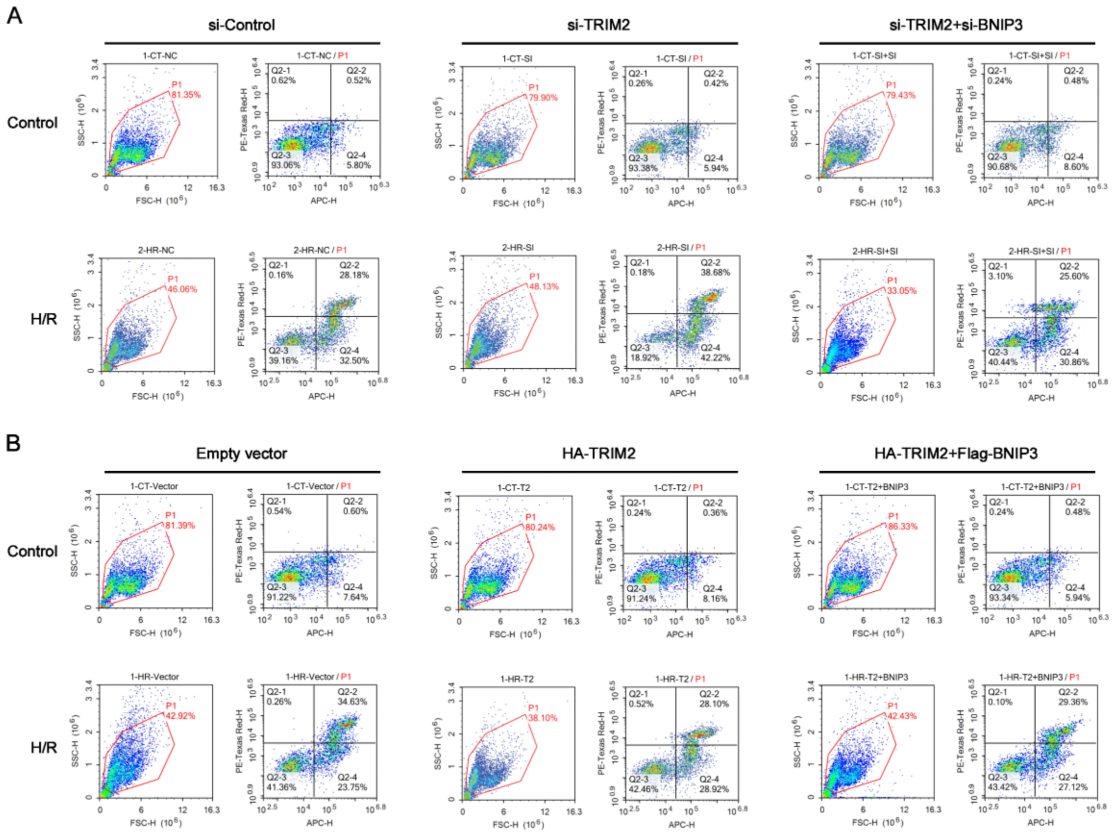

Supplementary Figure 13. Flow cytometric analysis of apoptosis using Annexin V and PI co-staining.

(A) Representative dot plot showing the gating strategy for quantifying apoptotic cells presented in Figure 8C. Cells in the lower right quadrant (Annexin V^+/PI^-) represent early apoptosis; cells in the upper right quadrant (Annexin V^+/PI^+) represent late apoptosis. (B) Representative dot plot showing the gating strategy for quantifying apoptotic cells presented in Figure 8H.

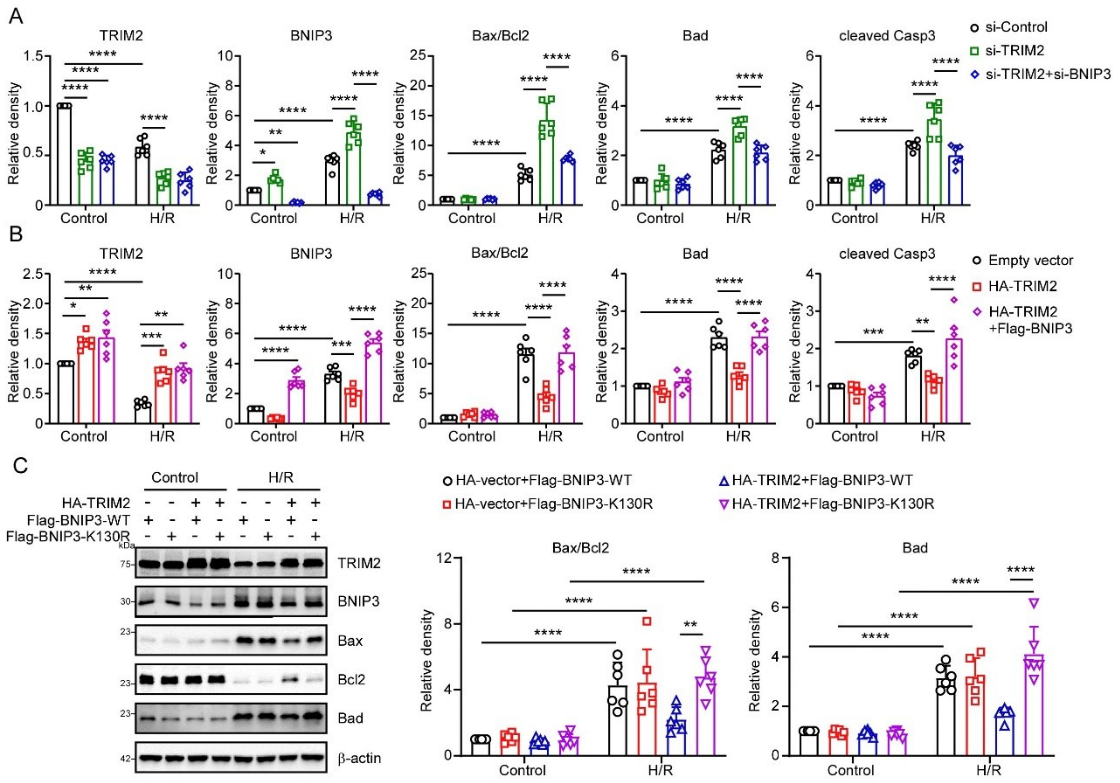

Supplementary Figure 14. TRIM2 catalyzes K48-linked polyubiquitination of BNIP3

(A) The quantification of the Western blot bands presented in Figure 8E was conducted using the Gel-Pro Analyzer software. (B) The quantification of the Western blot bands presented in Figure 8I was conducted using the Gel-Pro Analyzer software. The relative mRNA levels of Bax, Bcl-2, and Bad in the intestine were determined by qRT-PCR. (C) Western blot was used to detect the protein expression of TRIM2, BNIP3, Bax, Bcl-2, Bad and cleaved Casp3 in Caco-2 cells subjected to TRIM2 and BNIP3 protein or its K130R mutant overexpressed. The quantification of the Western blot bands was conducted using the Gel-Pro Analyzer software. All results are expressed as the mean ± SD. The statistical significance was determined using one-way ANOVA followed by Tukey's test, with the following levels of significance: * $p < 0.05$, ** $p < 0.01$, *** $p < 0.001$, and **** $p < 0.0001$.

Full unedited pictures for WB results

Figure 1C

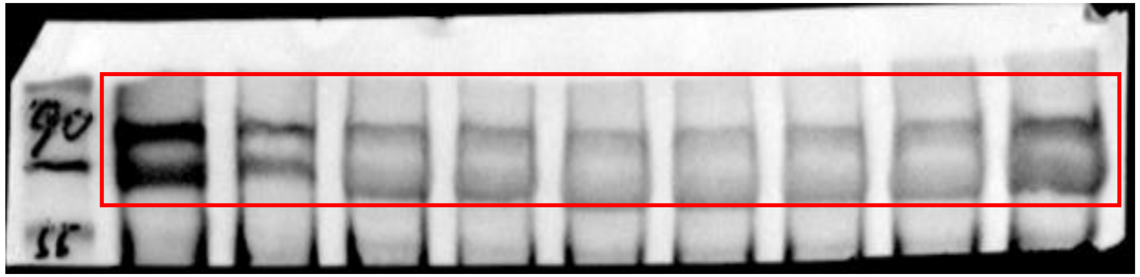

IEC-6 cell
IB: Trim2

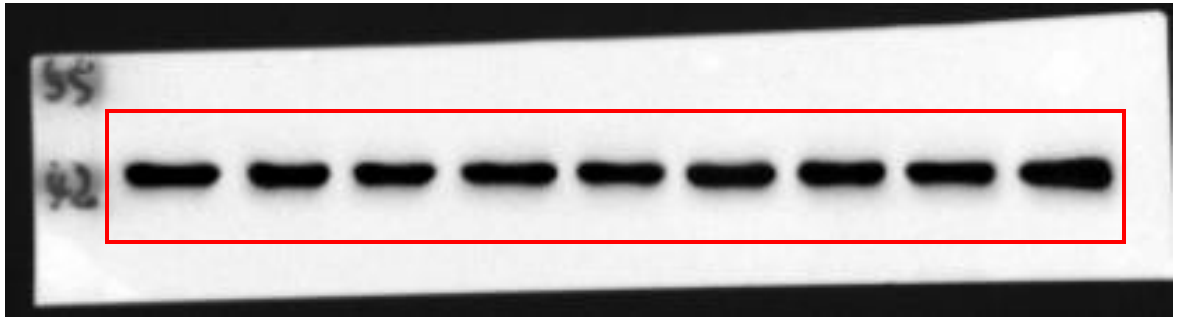

IEC-6 cell
IB: β-actin

Figure 1D

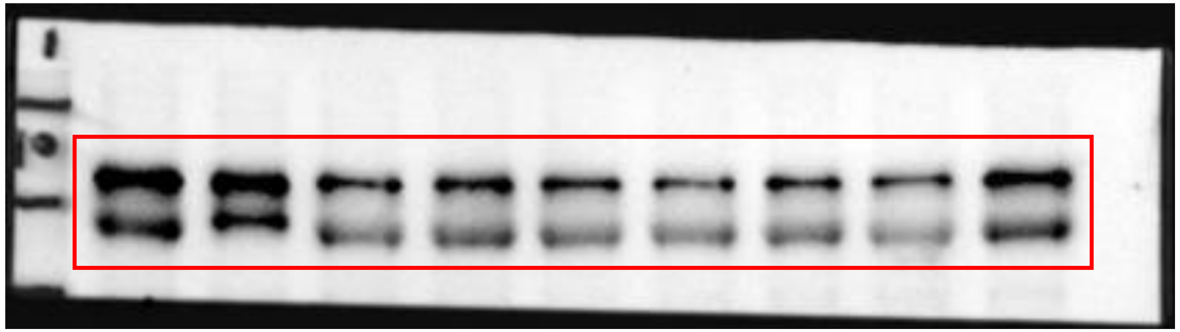

Caco-2 cell
IB: Trim2

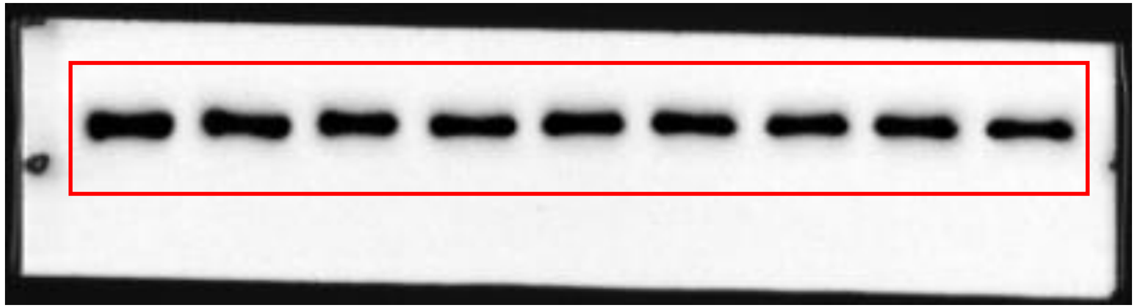

Caco-2 cell
IB: β-actin

Figure 1I

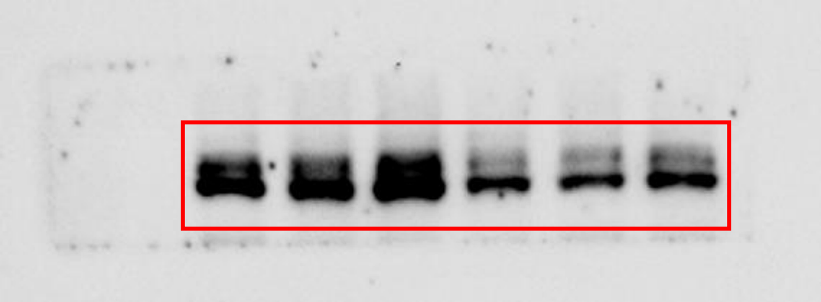

IB: Trim2

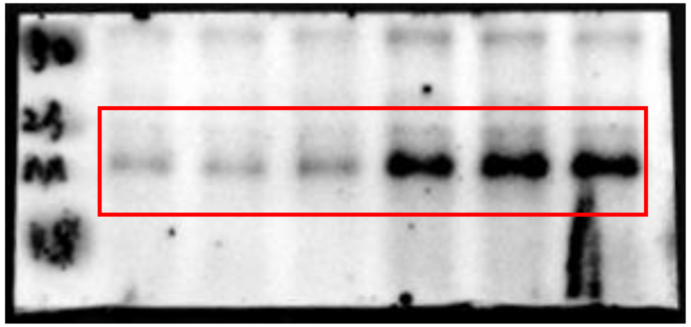

IB: Bax

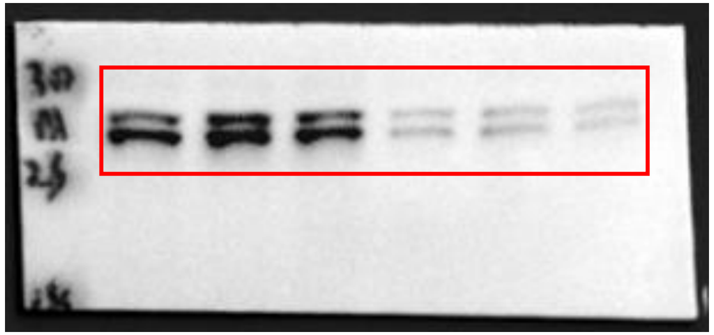

IB: Bcl-2

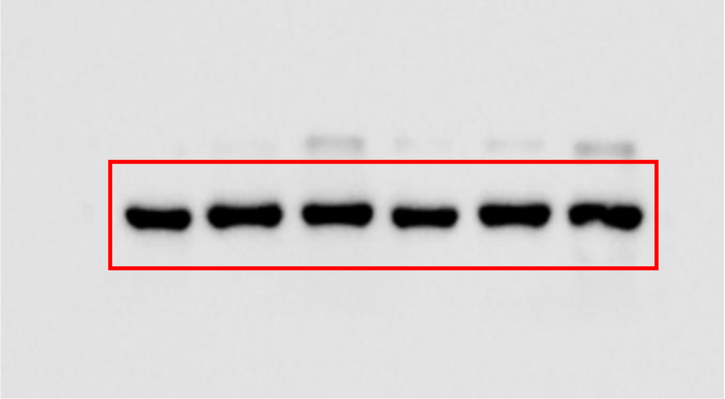

IB: β-actin

Figure 2D

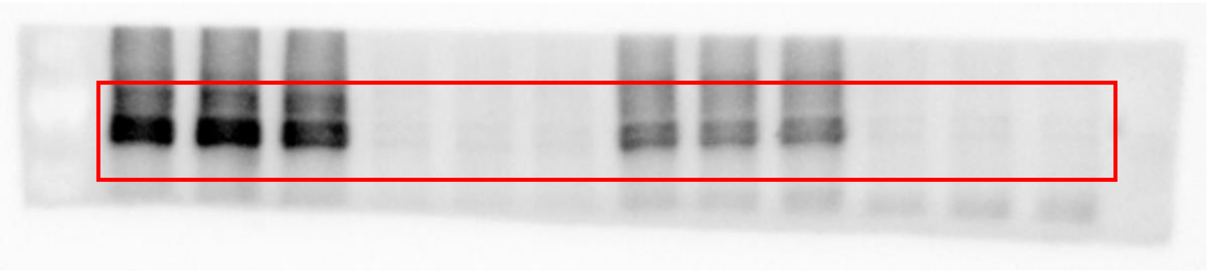

IB: Trim2

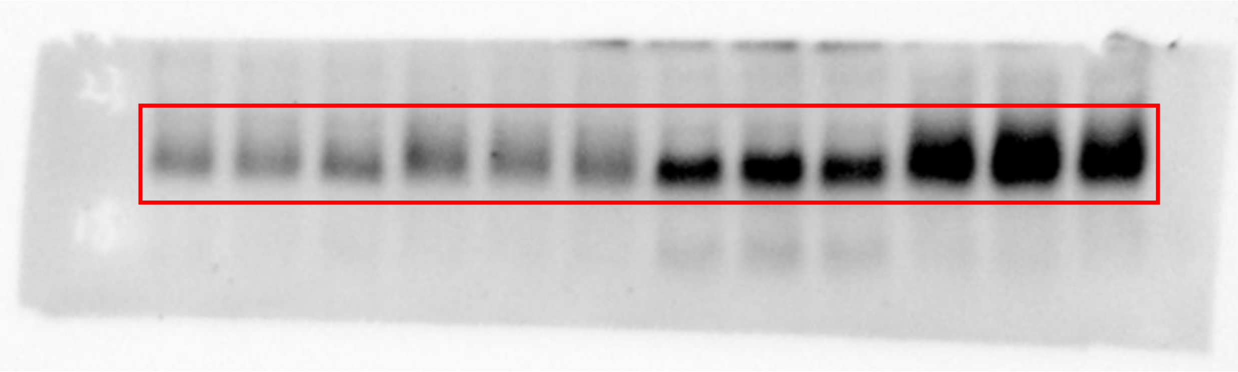

IB: Bad

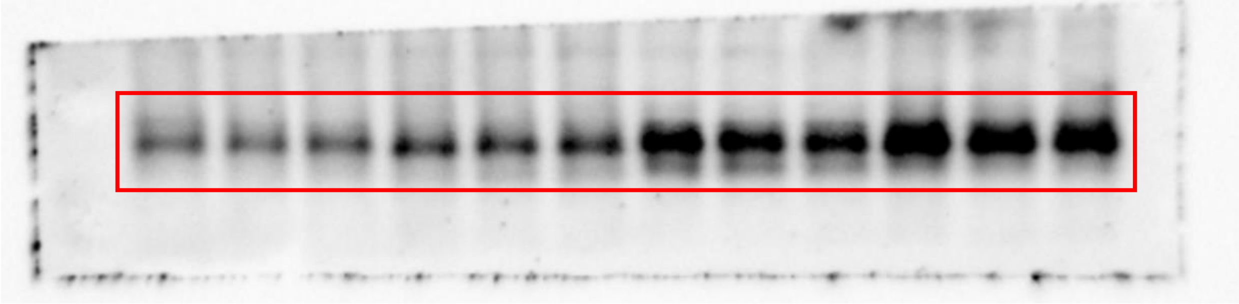

IB: Bax

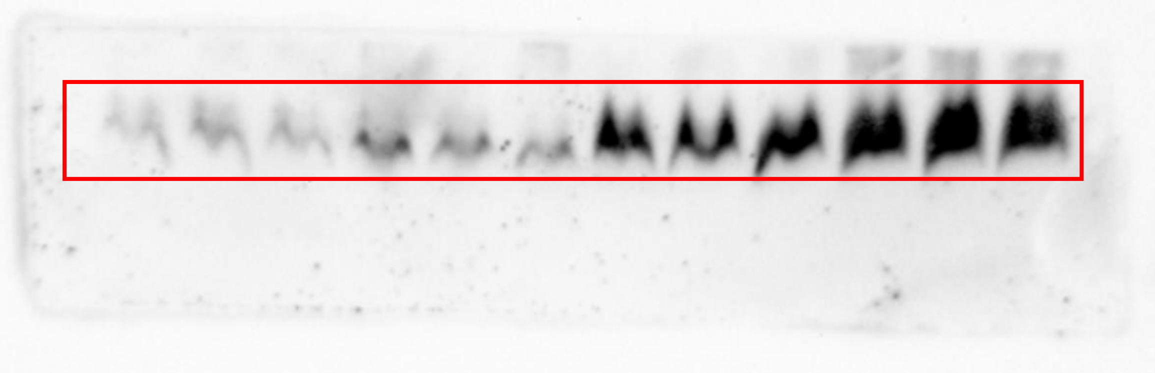

IB: Cleaved Casp3

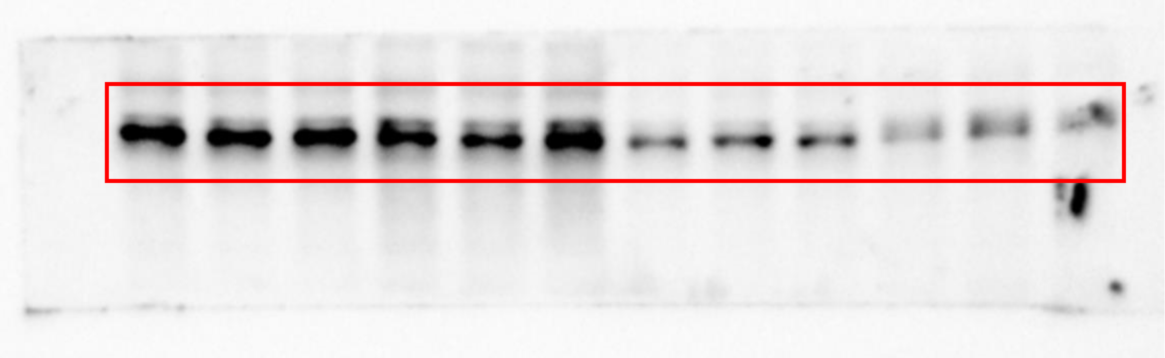

IB: Bcl-2

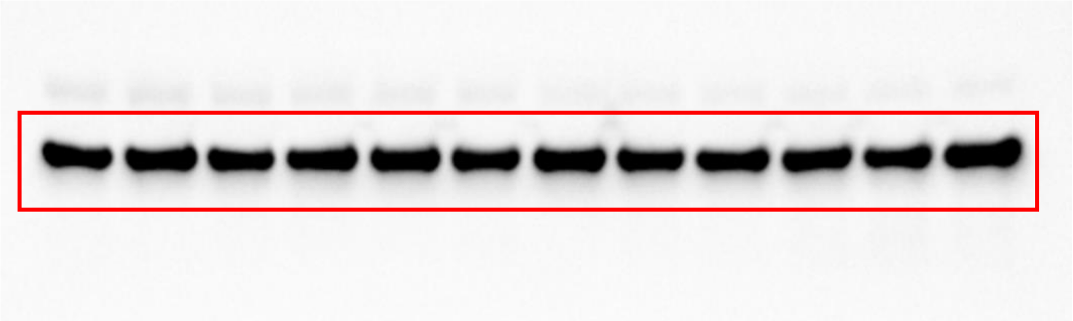

IB: β-actin

Figure 3B

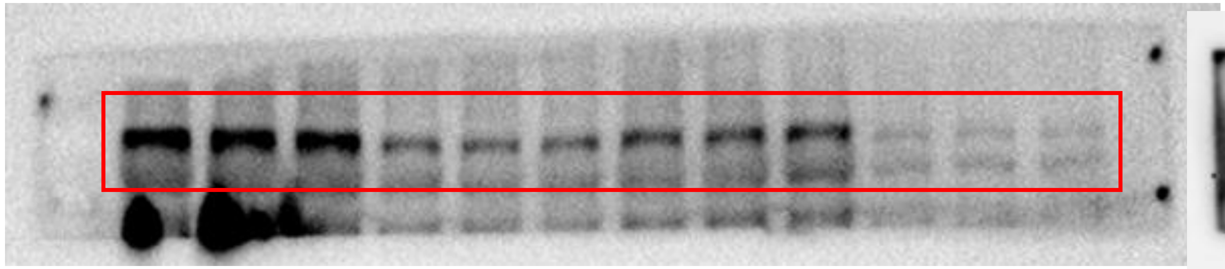

IEC-6 cell
IB: Trim2

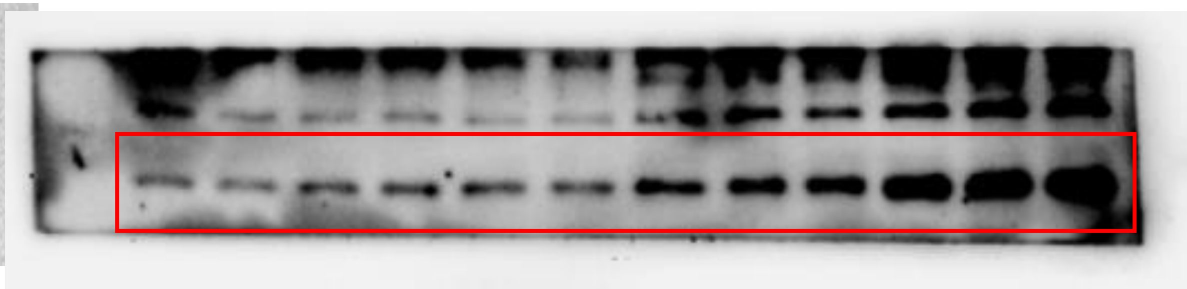

IEC-6 cell
IB: Bad

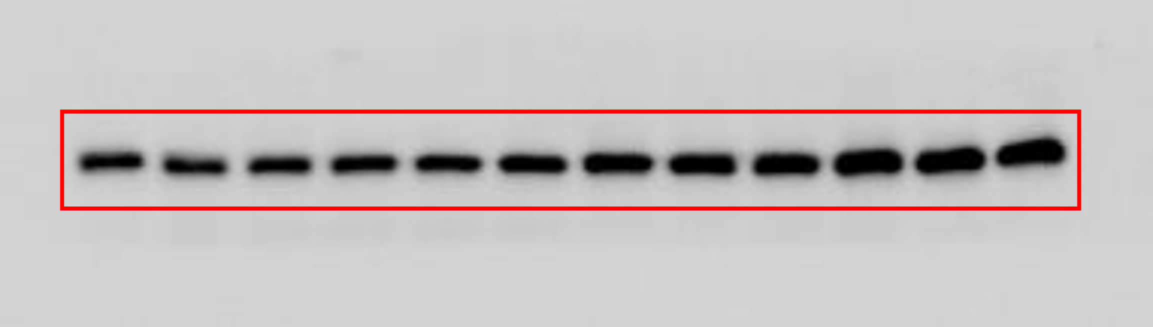

IEC-6 cell
IB: Bax

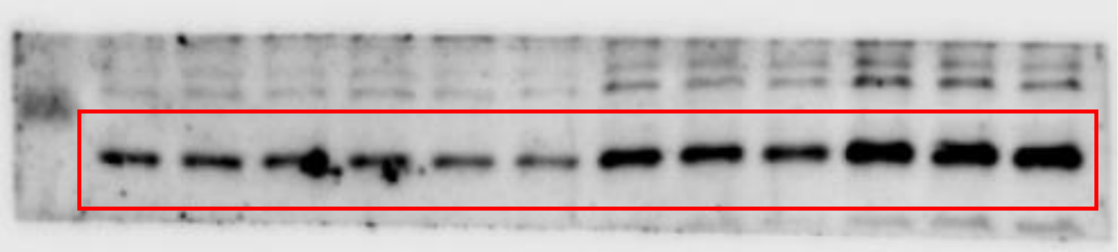

IEC-6 cell
IB: Cleaved Casp3

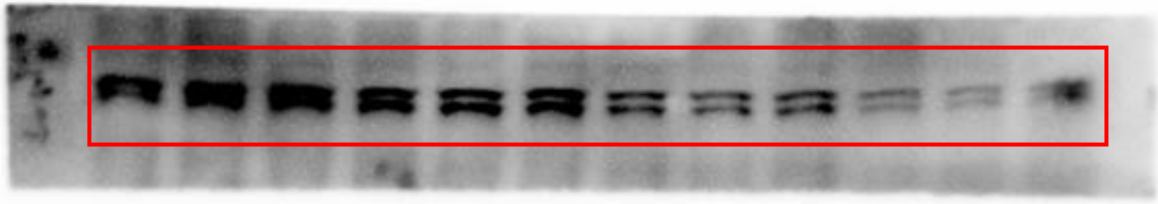

IEC-6 cell
IB: Bcl-2

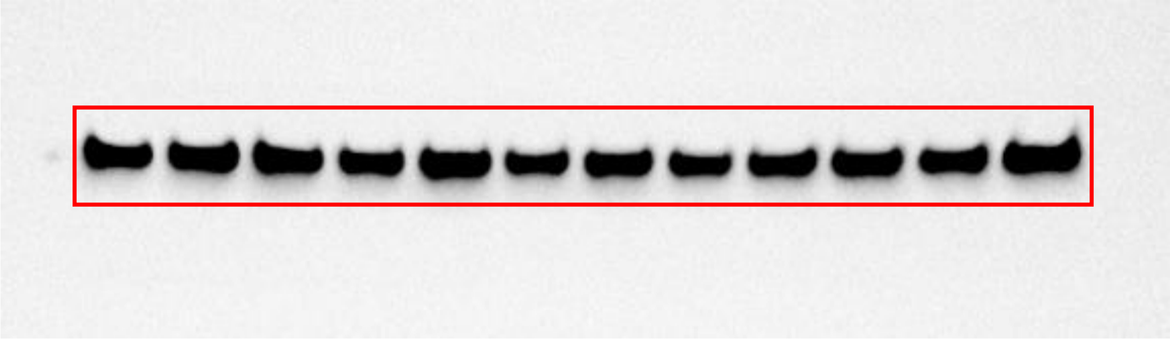

IEC-6 cell
IB: β-actin

Figure 3B

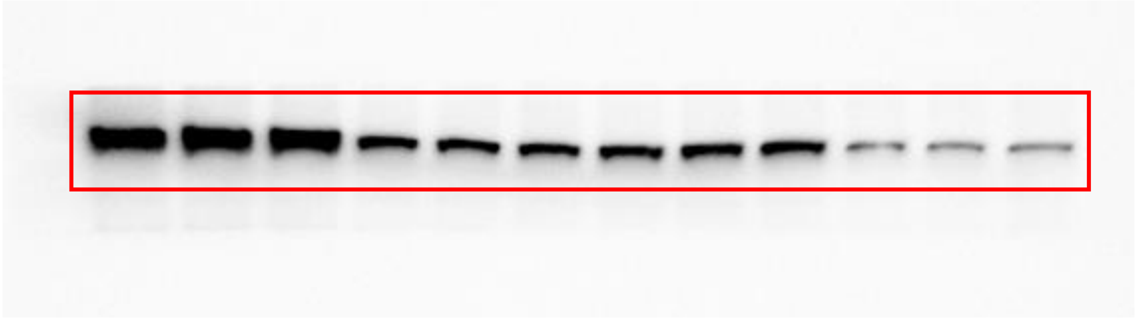

Caco-2 cell
IB: Trim2

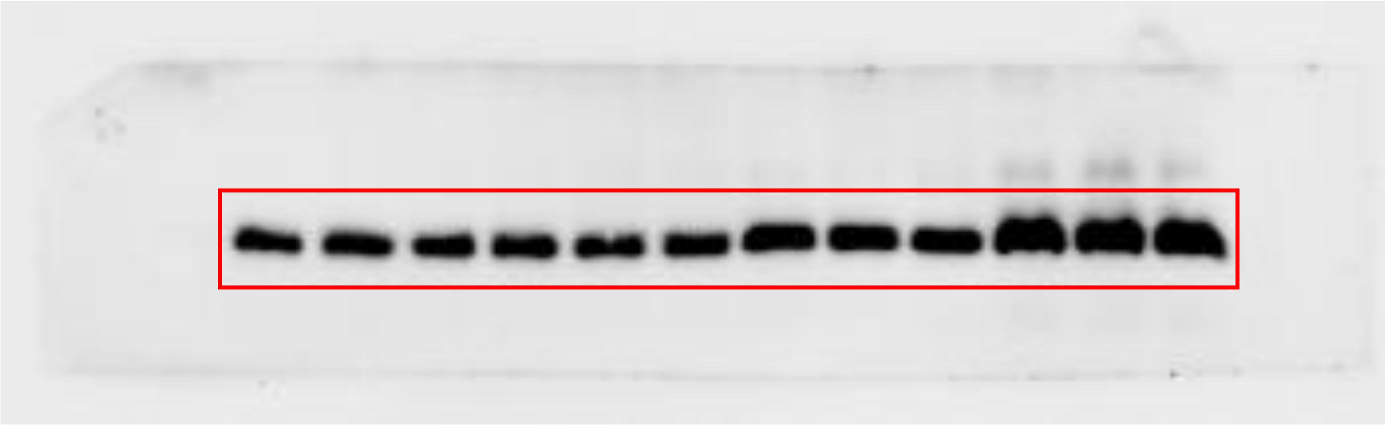

Caco-2 cell
IB: Bad

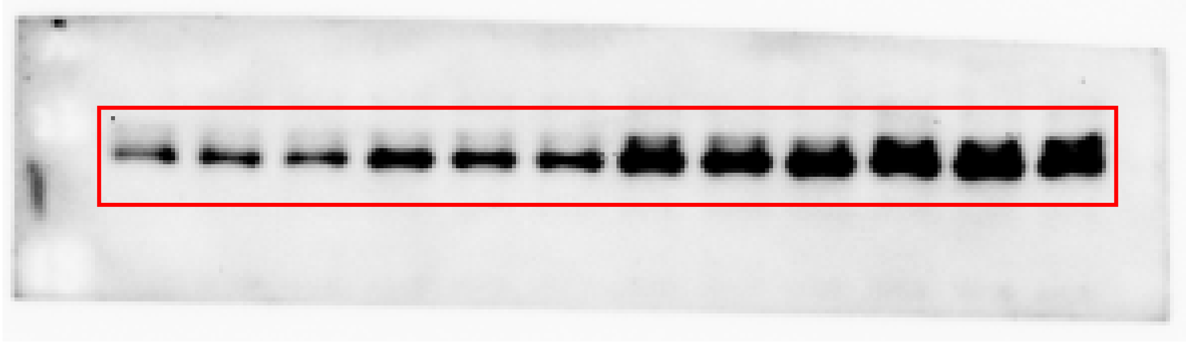

Caco-2 cell
IB: Bax

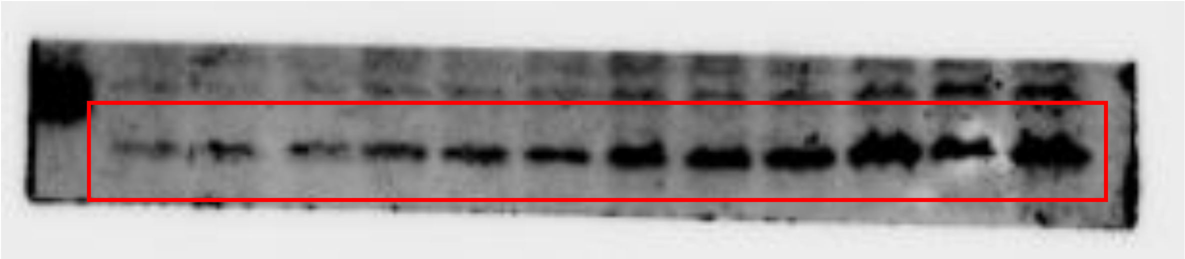

Caco-2 cell
IB: cleaved Casp3

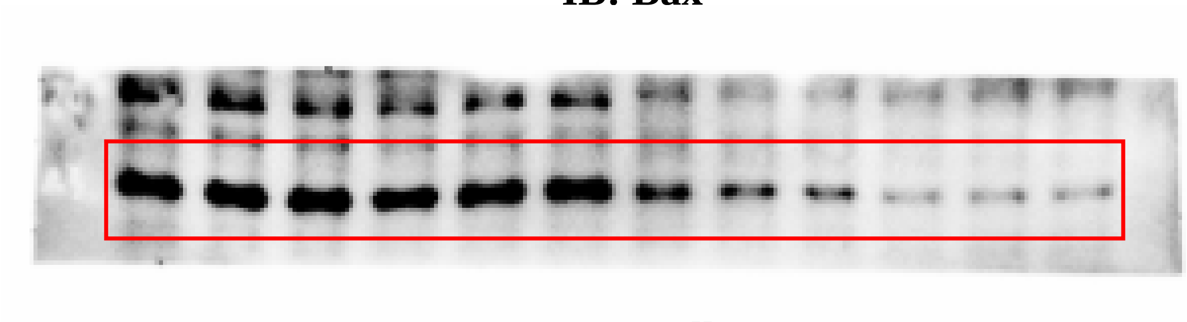

Caco-2 cell
IB: Bcl-2

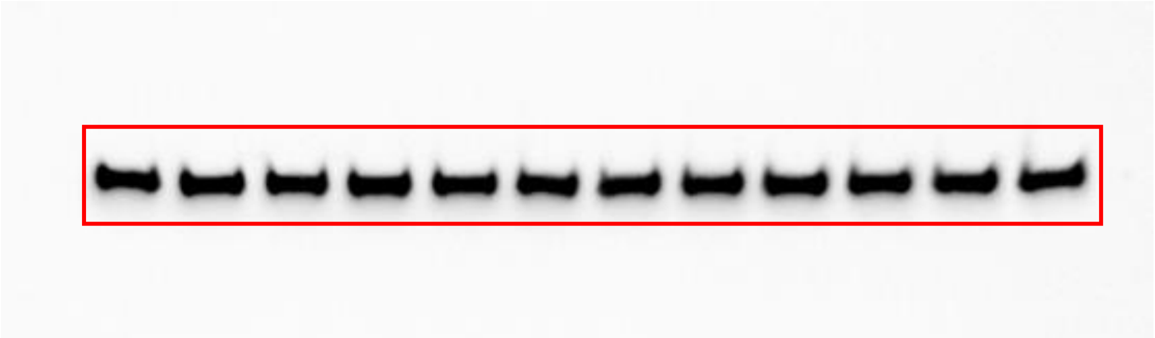

Caco-2 cell
IB: β-actin

Figure 4C

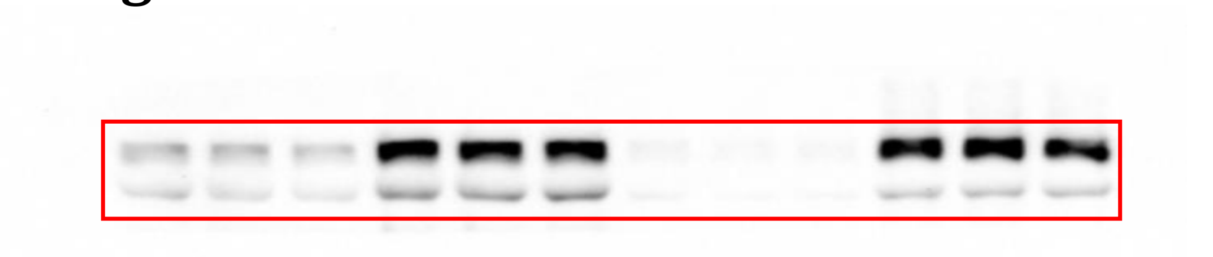

IEC-6 cell
IB: Trim2

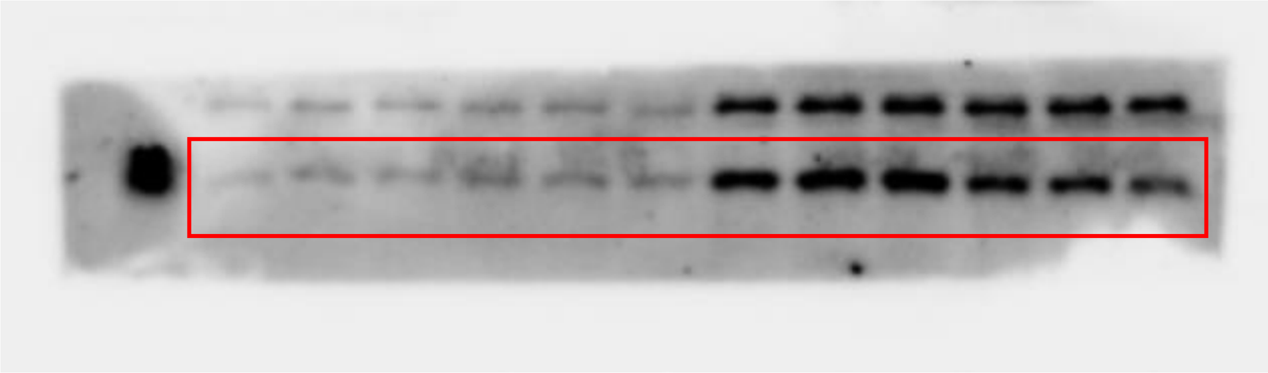

IEC-6 cell
IB: Bad

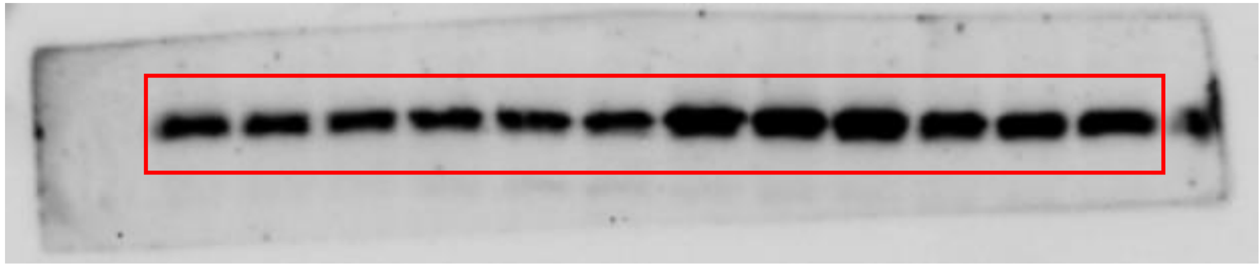

IEC-6 cell
IB: Bax

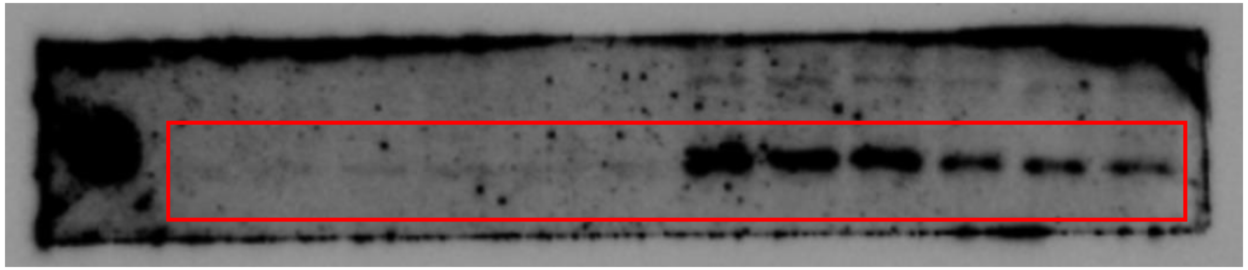

IEC-6 cell
IB: Cleaved Casp3

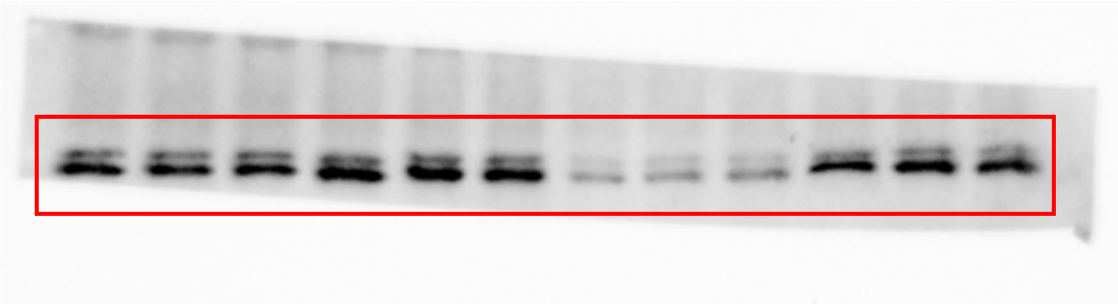

IEC-6 cell
IB: Bcl-2

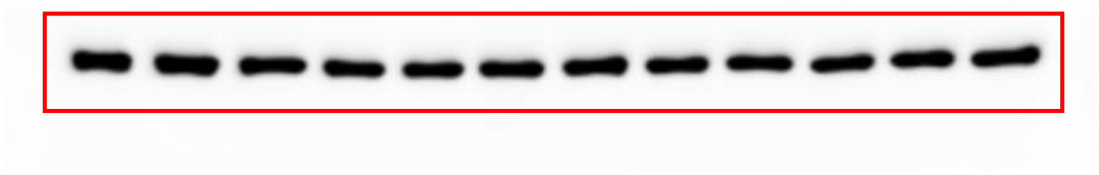

IEC-6 cell
IB: β-actin

Figure 4C

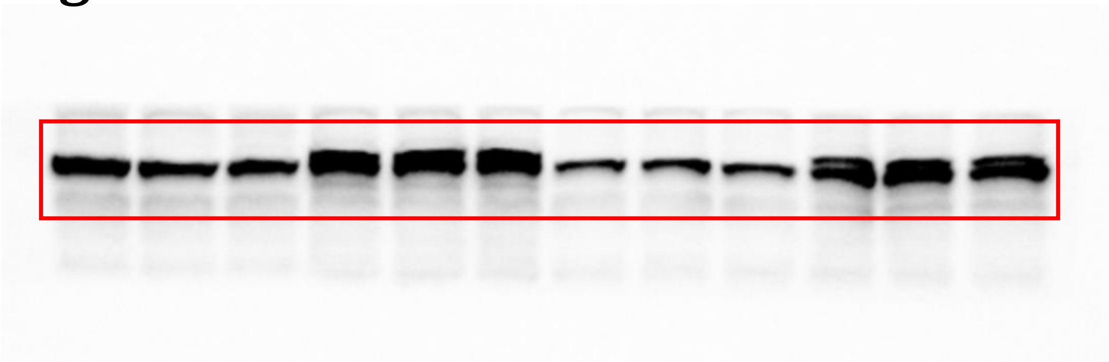

Caco-2 cell
IB: Trim2

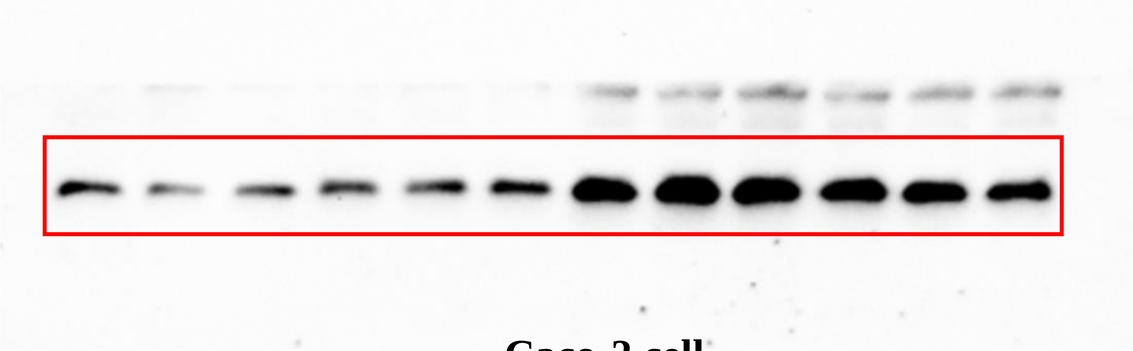

Caco-2 cell
IB: Bax

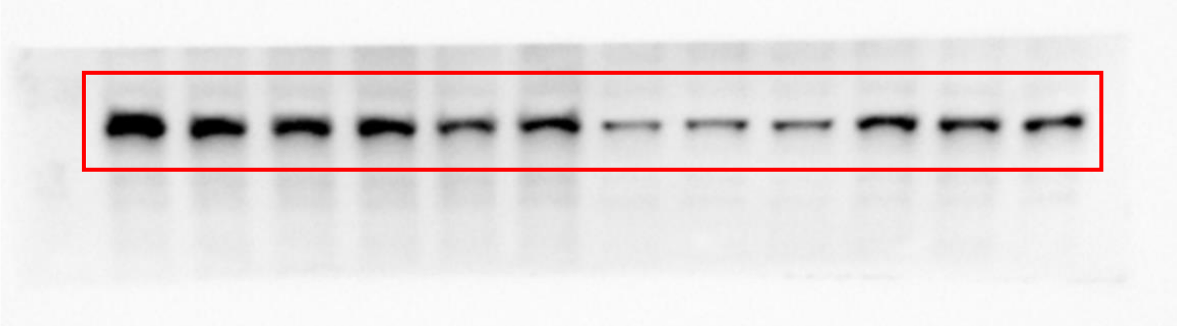

Caco-2 cell
IB: Bcl-2

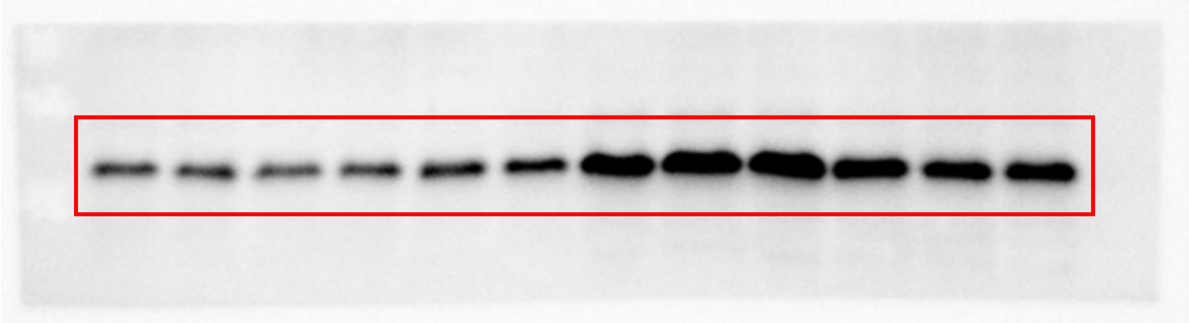

Caco-2 cell
IB: Bad

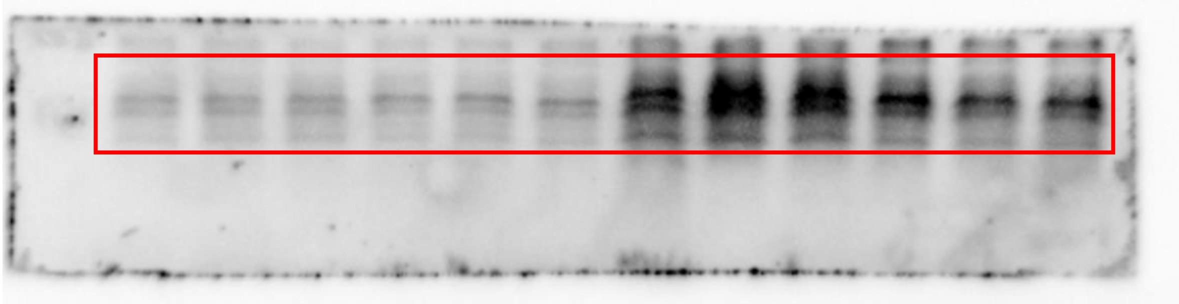

Caco-2 cell
IB: cleaved Casp3

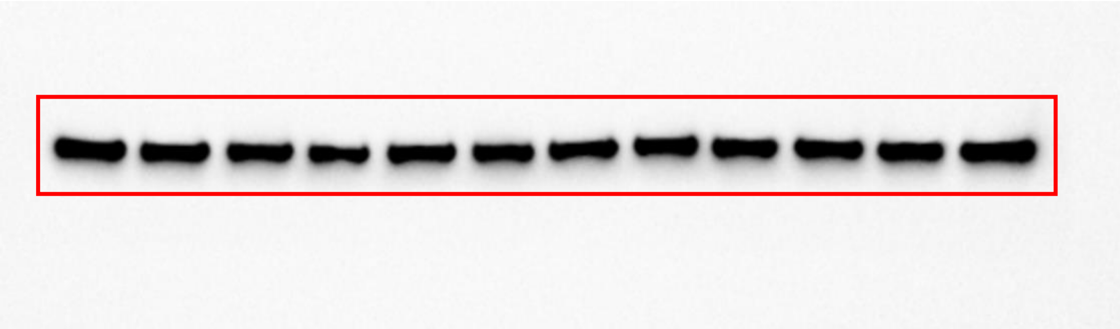

Caco-2 cell
IB: β-actin

Figure 5A

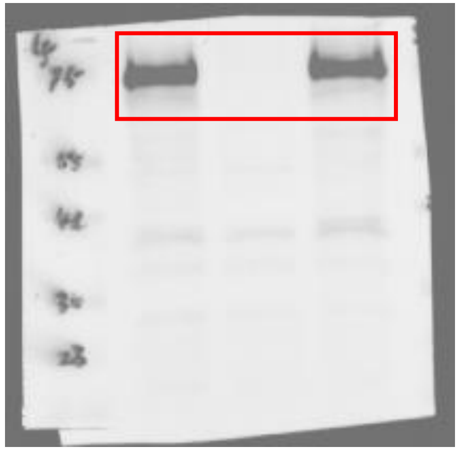

WCL
IB: HA

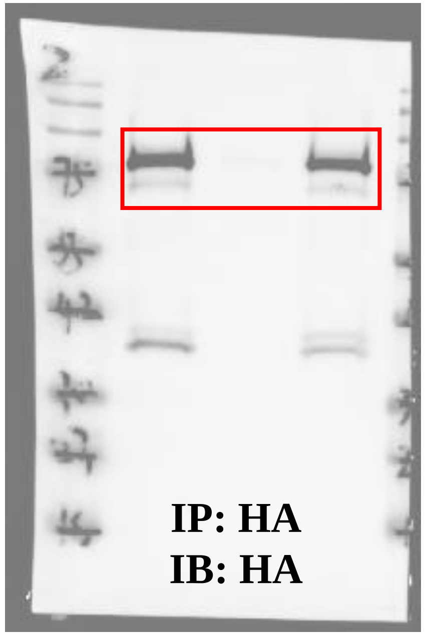

IP: HA
IB: HA

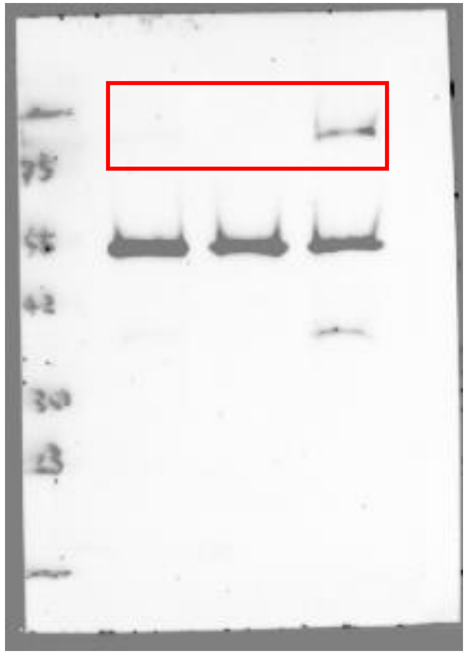

IP: Flag
IB: HA

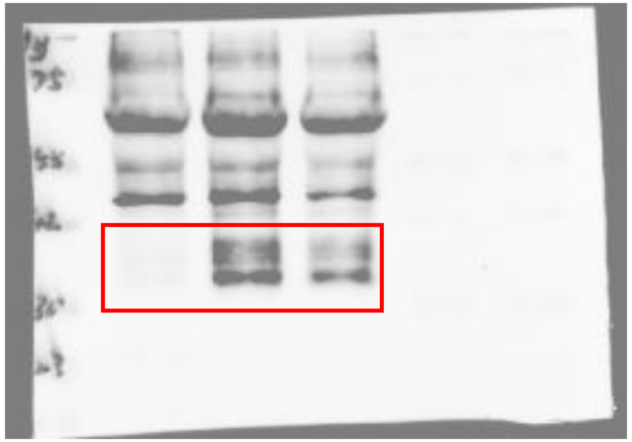

WCL
IB: Flag

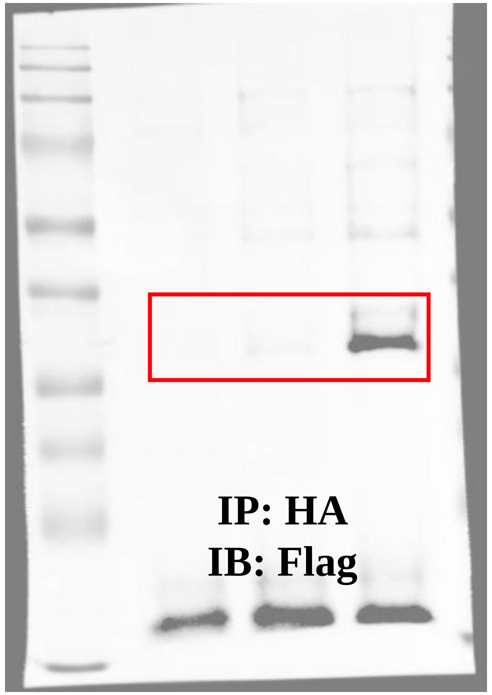

IP: HA
IB: Flag

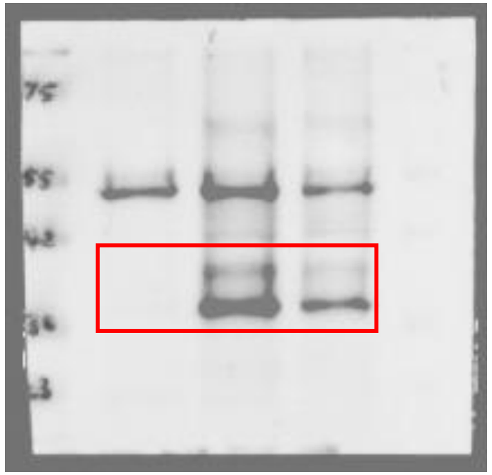

IP: Flag
IB: Flag

Figure 5B

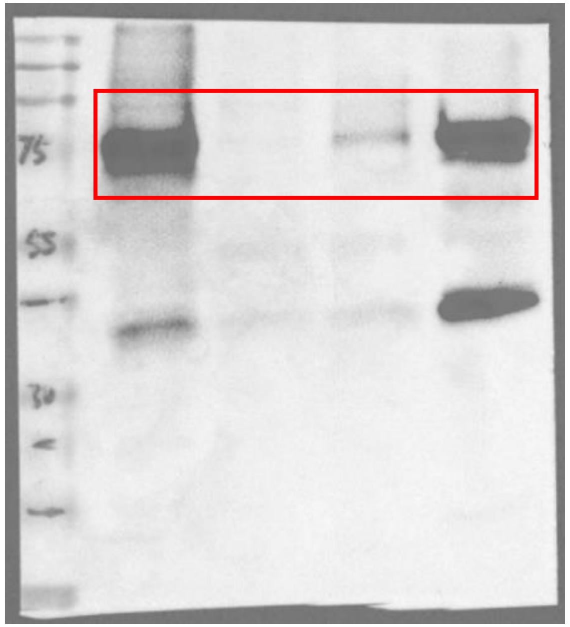

IB: HA

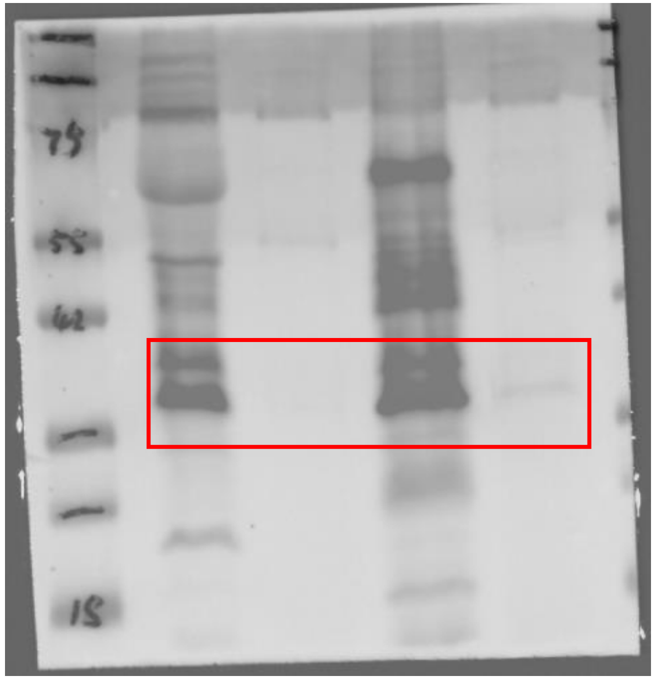

IB: Flag

Figure 5C

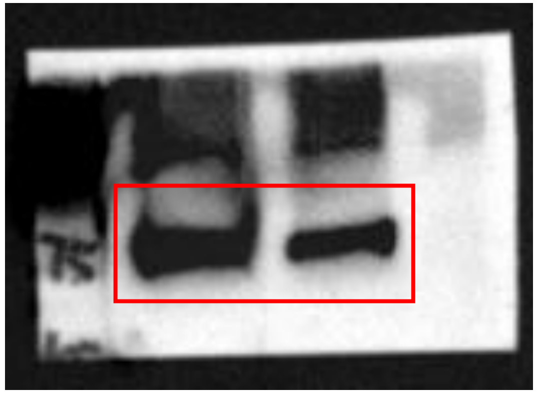

WCL
IB: Trim2

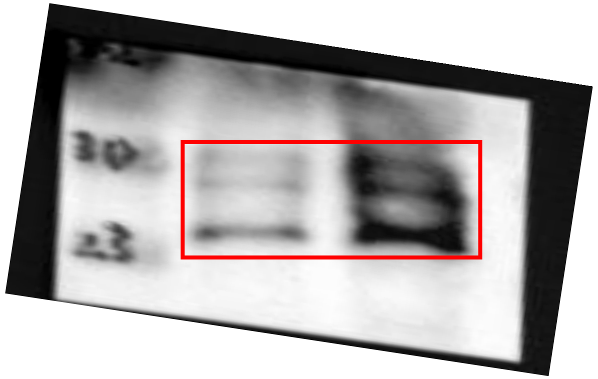

WCL
IB: Bnip3

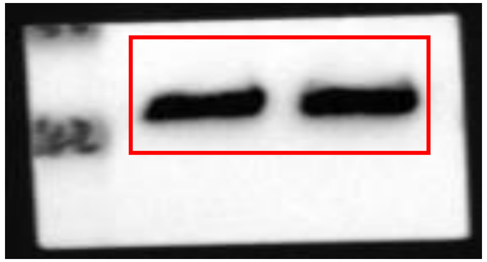

WCL
IB: β-actin

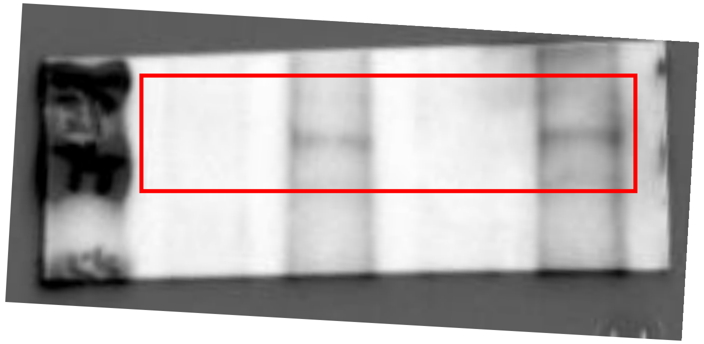

IP: Trim2 (M)
IB: Trim2 (R)

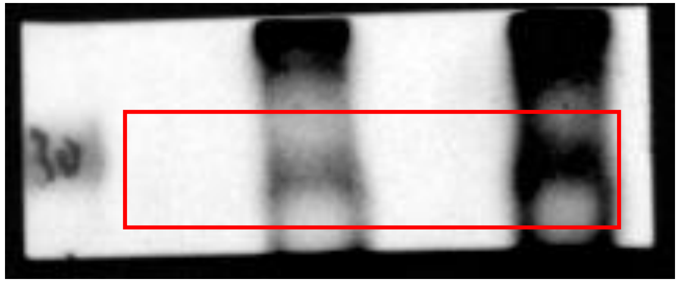

IP: Trim2 (M)
IB: Bnip3 (R)

Figure 5E

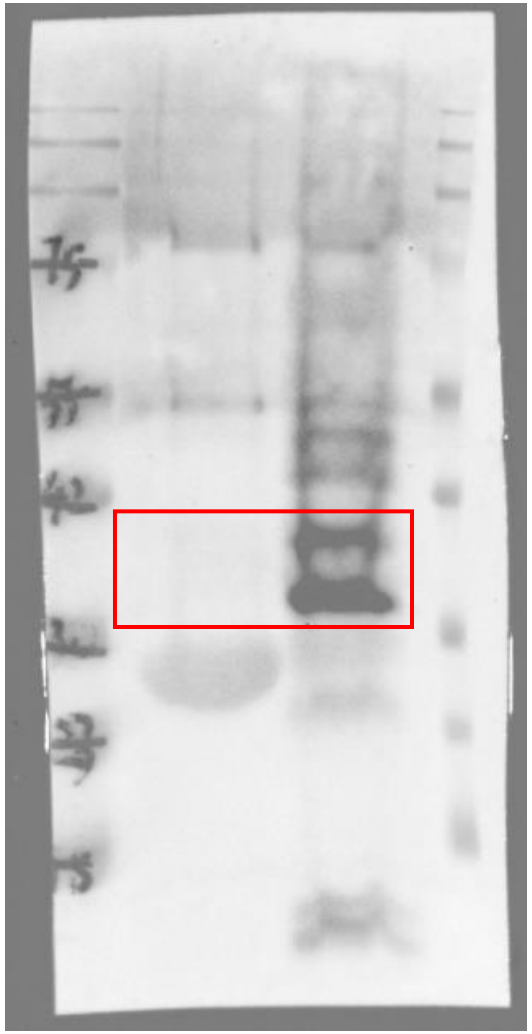
GST pull-down
IB: Flag

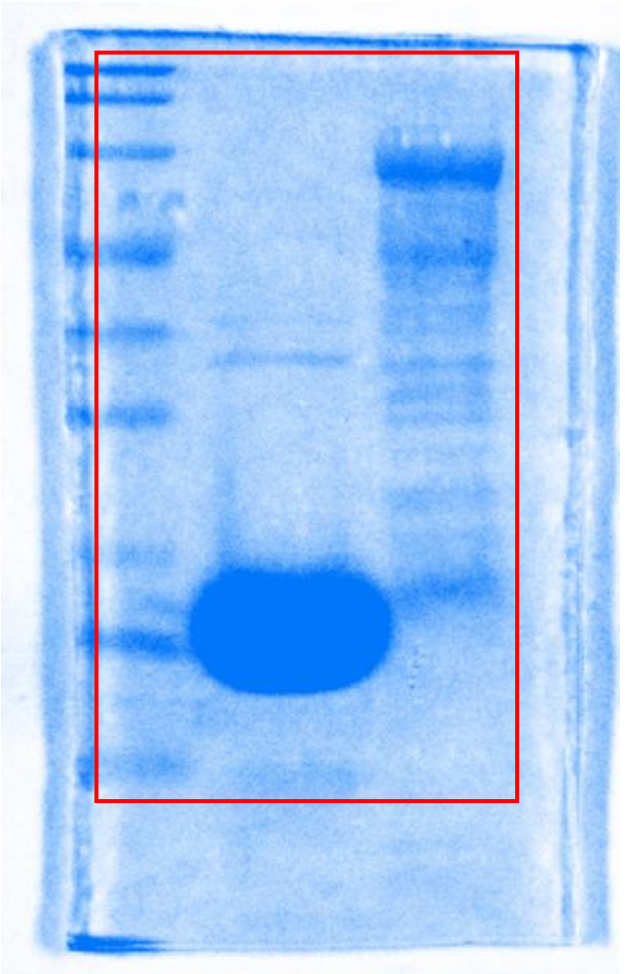
GST pull-down
Coomassie blue

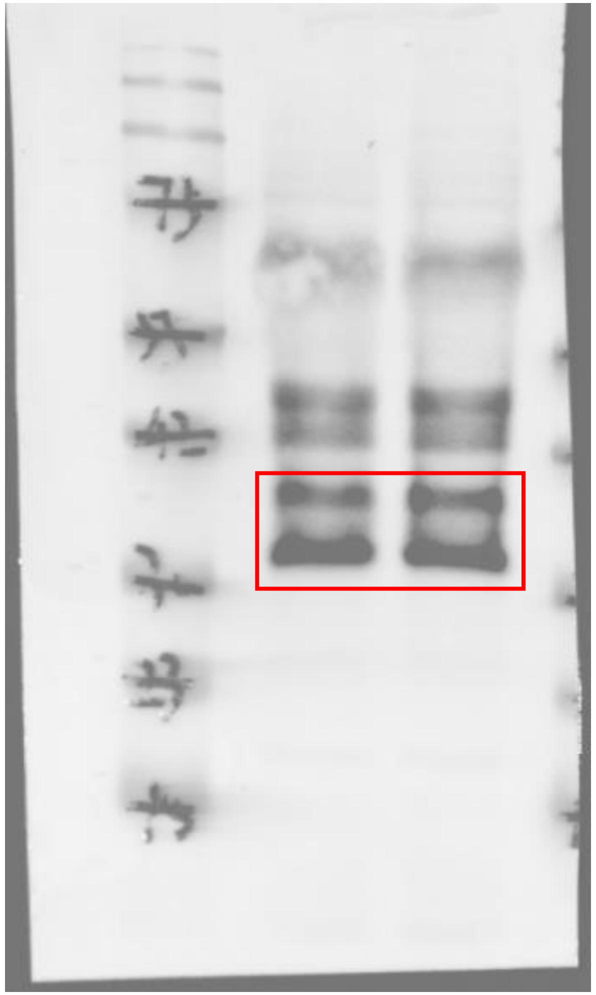
Input
IB: Flag

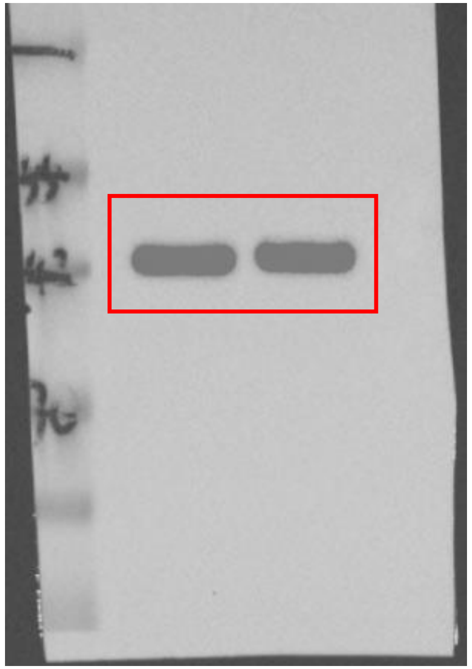
Input
IB: β-actin

Figure 5F

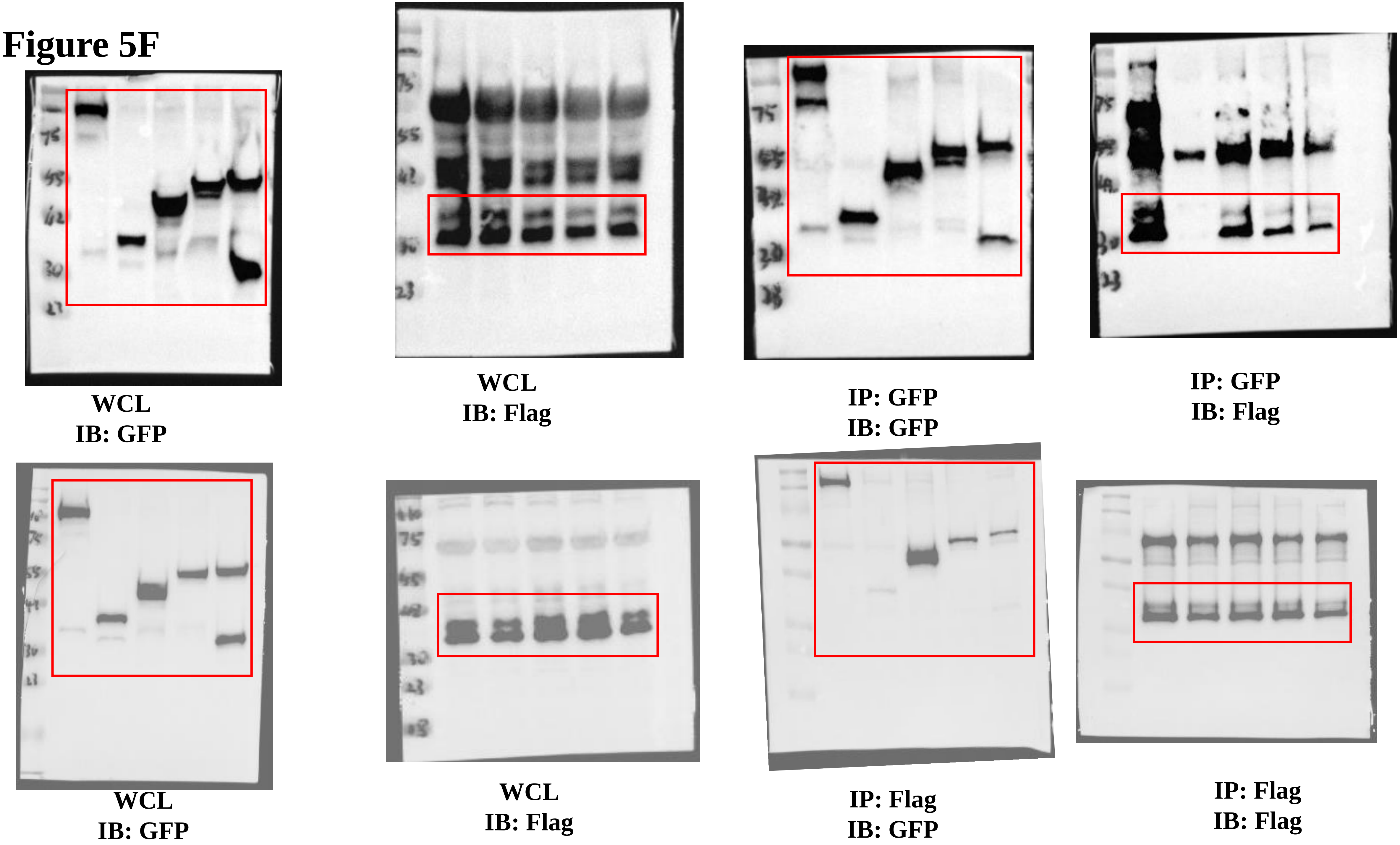
WCL
IB: GFP
WCL
IB: Flag
IP: GFP
IB: GFP
IP: GFP
IB: Flag
WCL
IB: GFP
WCL
IB: Flag
IP: Flag
IB: GFP
IP: Flag
IB: Flag

Figure 5H

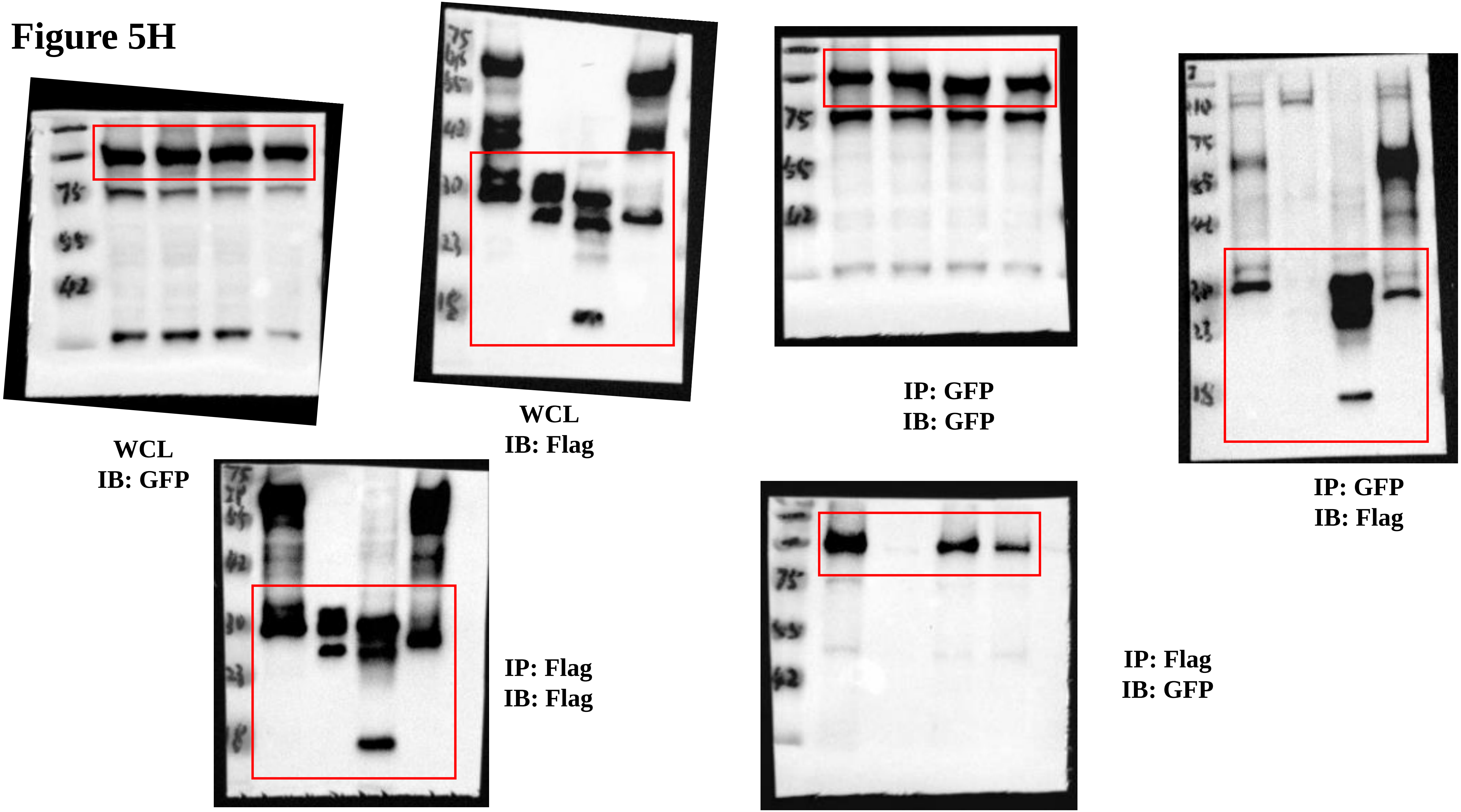

WCL
IB: GFP

WCL
IB: Flag

IP: GFP
IB: GFP

IP: GFP
IB: Flag

IP: Flag
IB: Flag

IP: Flag
IB: GFP

Figure 5F

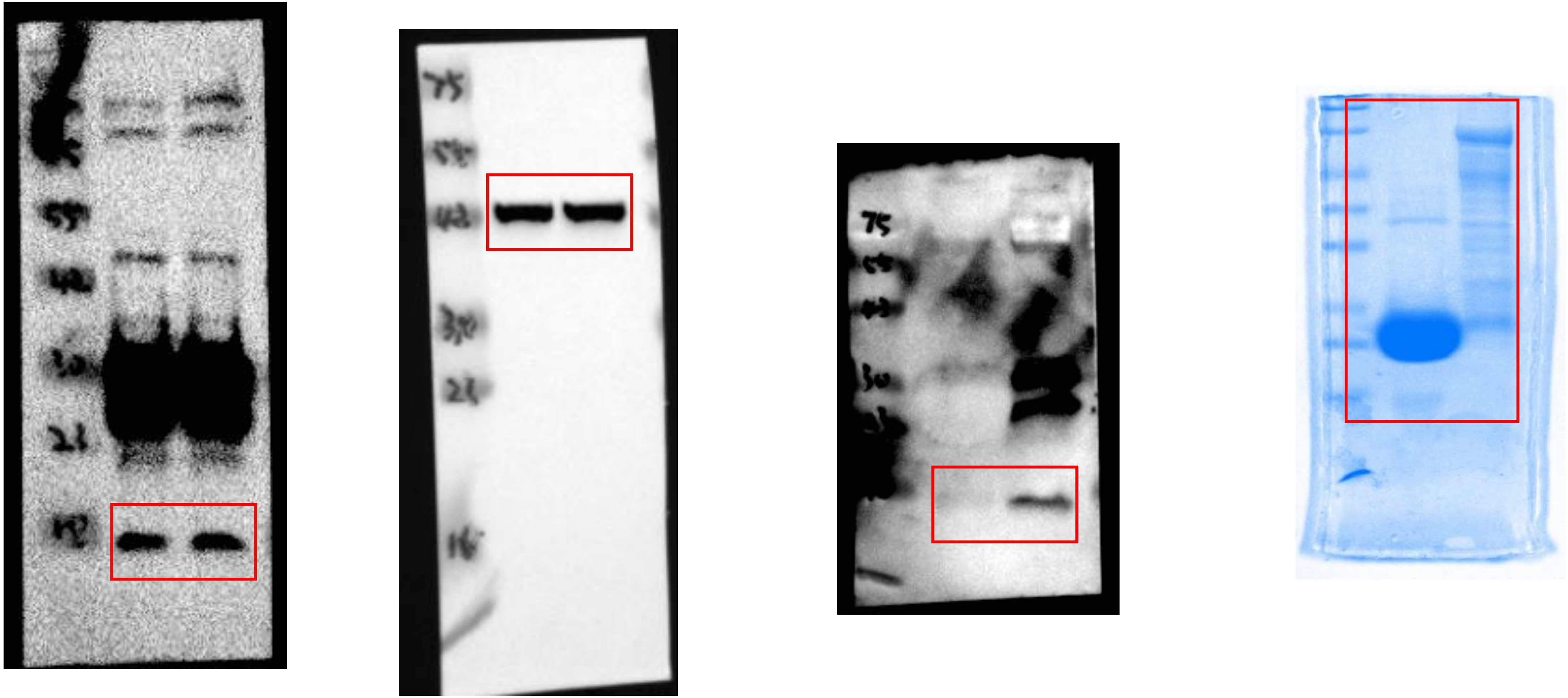

Input (95-194)
IB: Flag

Input (95-194)
IB: β-actin

GST pull-down (95-194)
IB: Flag

GST pull-down (95-194)
Coomassie

Figure 5F

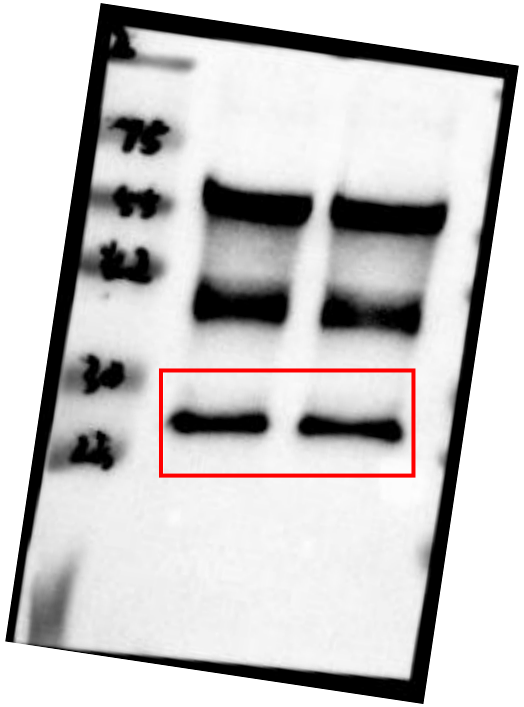

Input (del-BH3)
IB: Flag

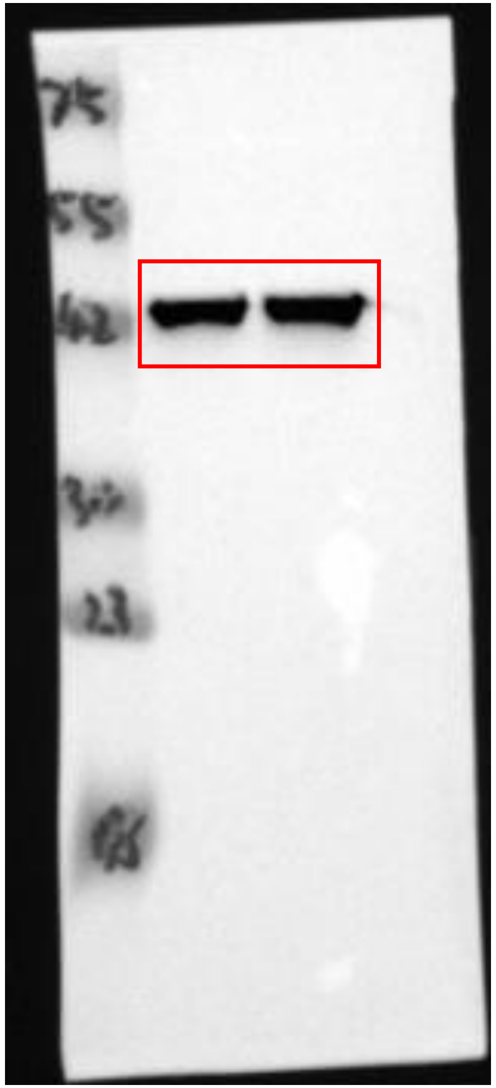

Input (del-BH3)
IB: β-actin

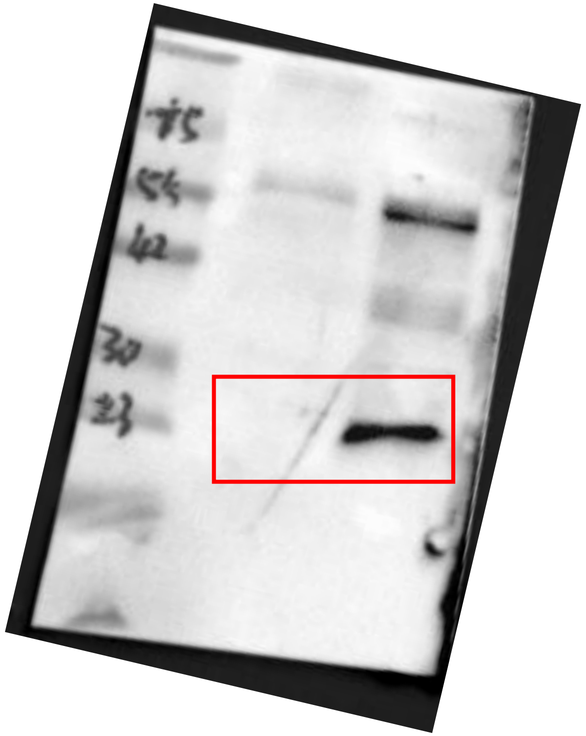

GST pull-down (del-BH3)
IB: Flag

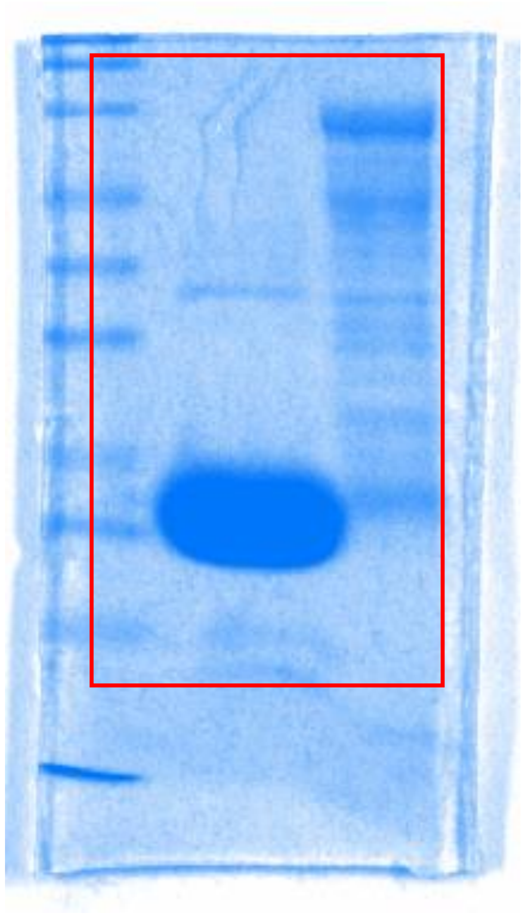

GST pull-down (del-BH3)
Coomassie

Figure 6A

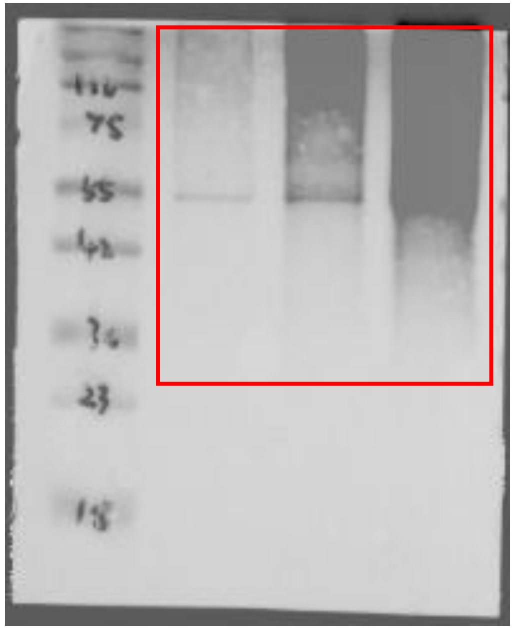

IP: Flag
IB: HA

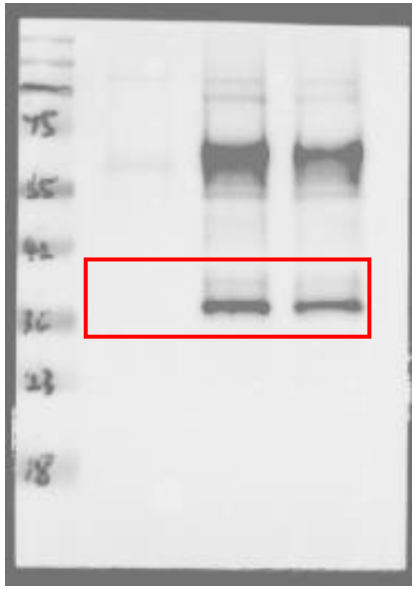

IP: Flag
IB: Flag

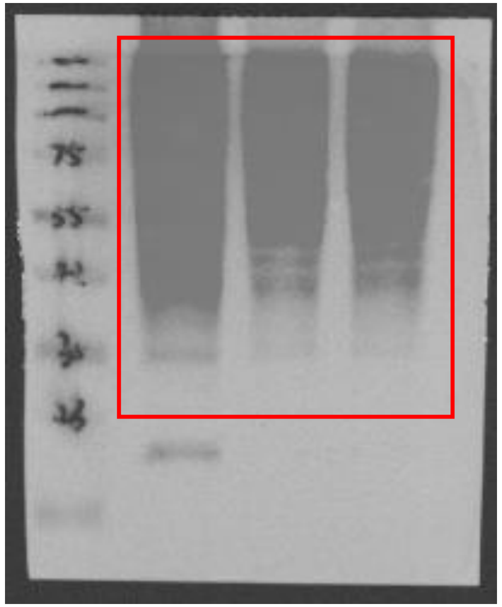

WCL
IB: HA

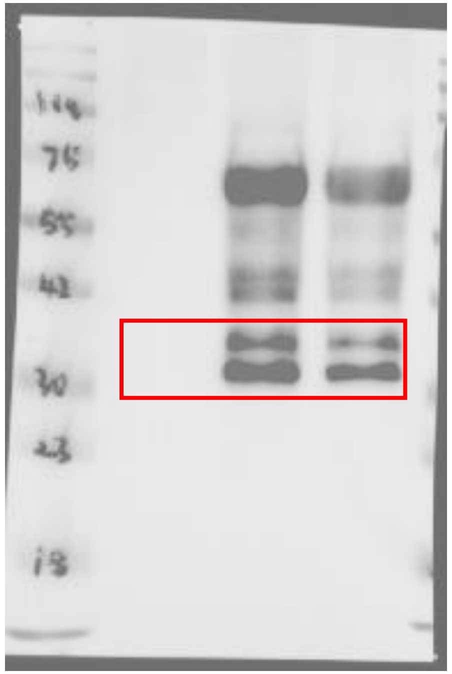

WCL
IB: Flag

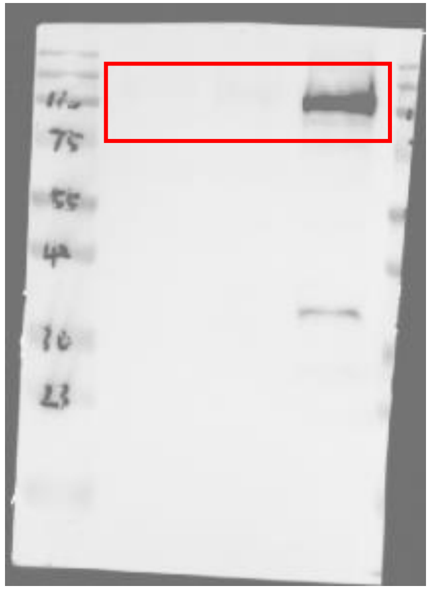

WCL
IB: GFP

Figure 6B

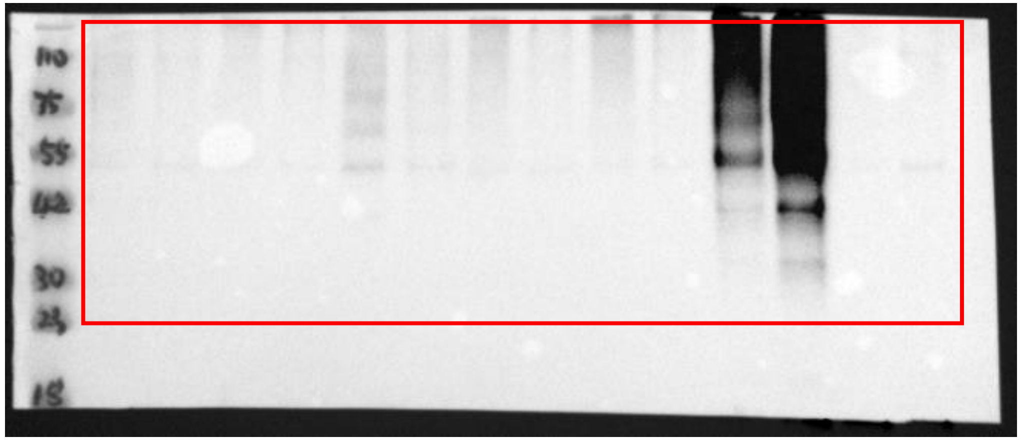

IP: Flag
IB: HA

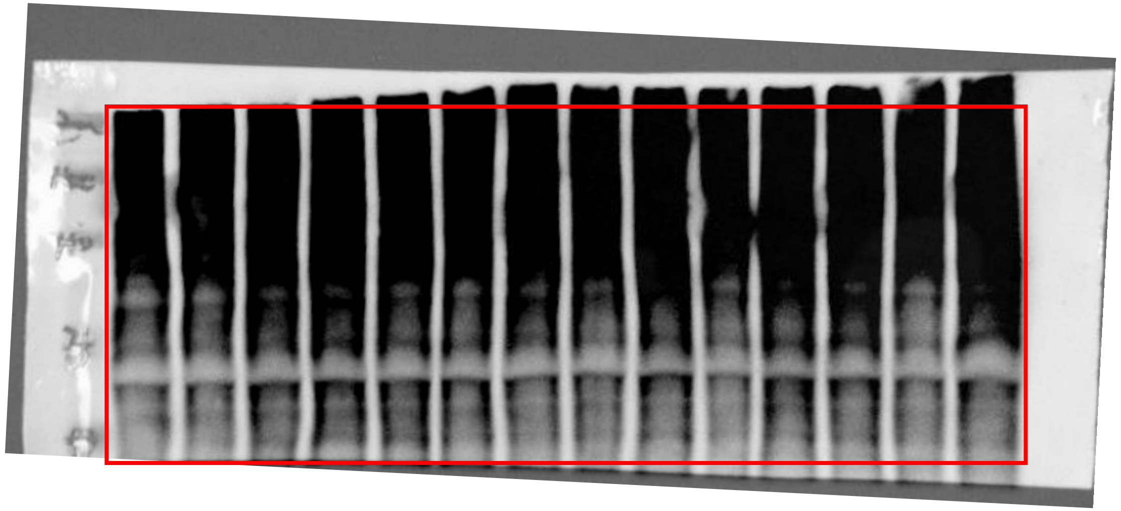

WCL
IB: HA

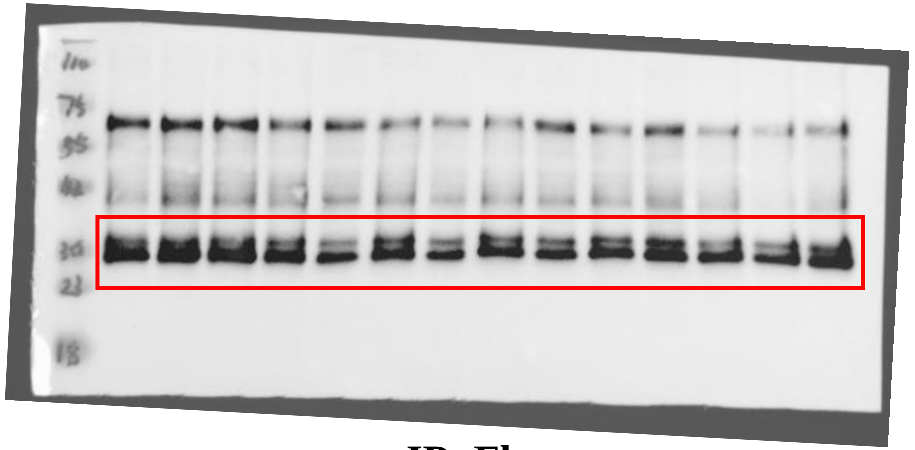

IP: Flag
IB: Flag

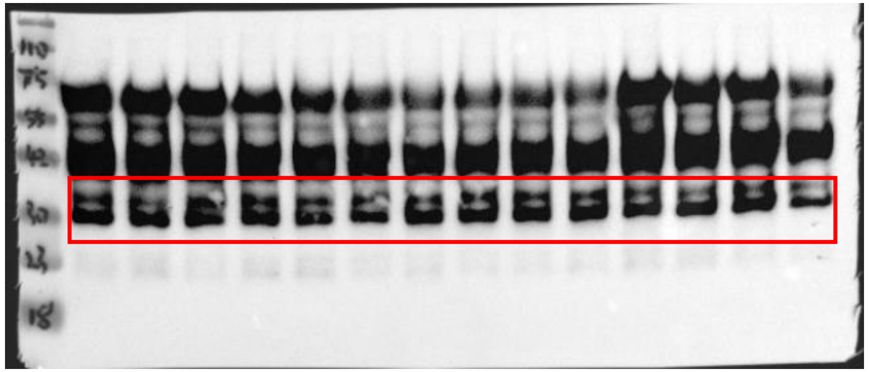

WCL
IB: Flag

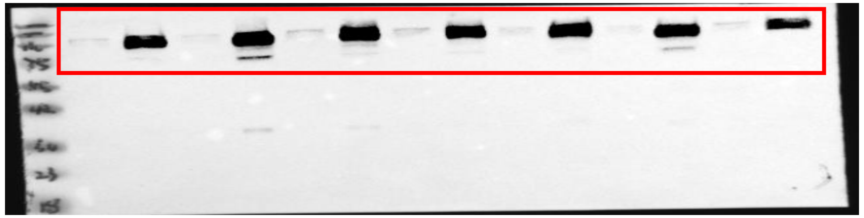

WCL
IB: GFP

Figure 6C

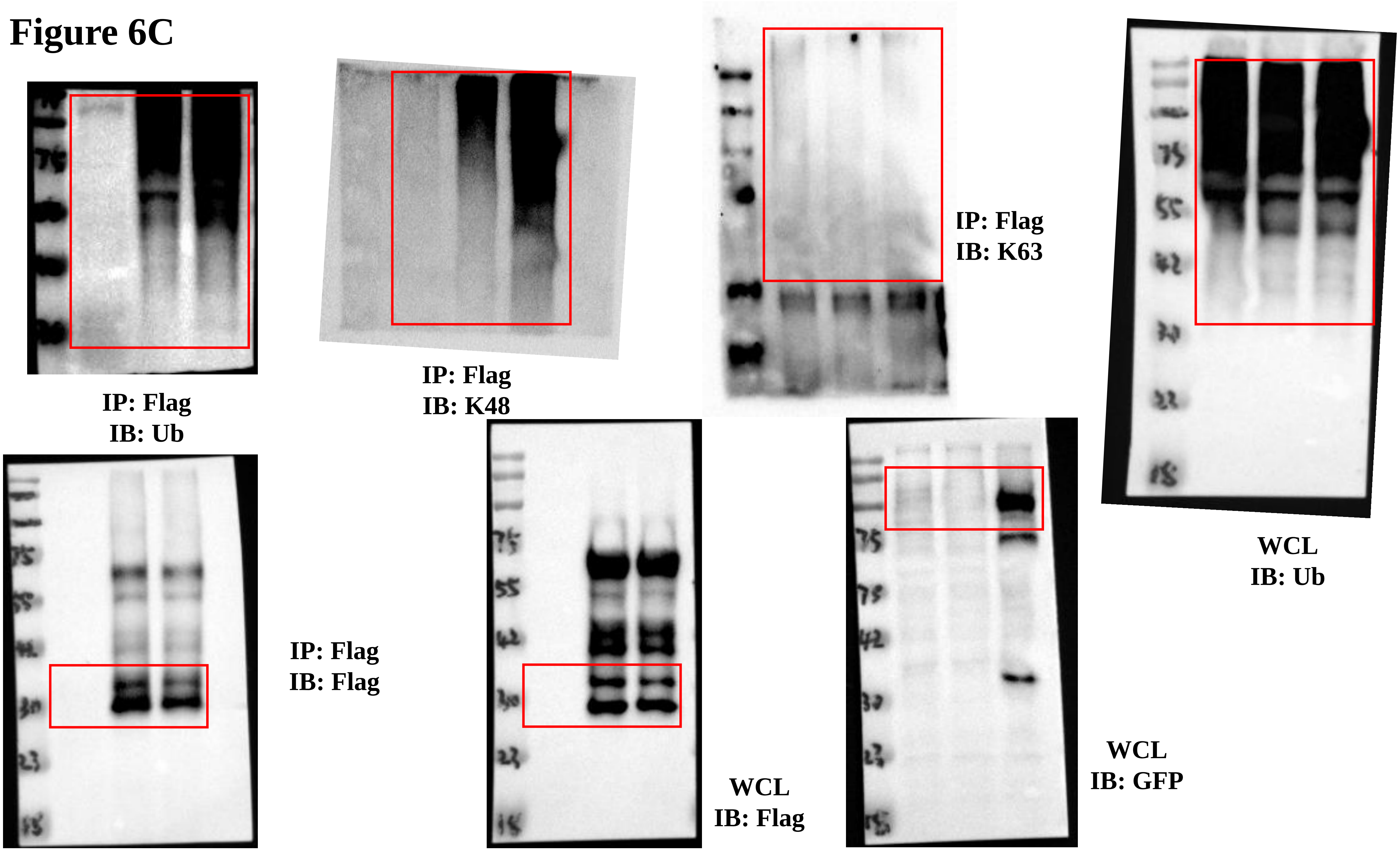
IP: Flag
IB: Ub
IP: Flag
IB: K48
IP: Flag
IB: K63
WCL
IB: Ub
IP: Flag
IB: Flag
WCL
IB: Flag
WCL
IB: GFP

Figure 6D

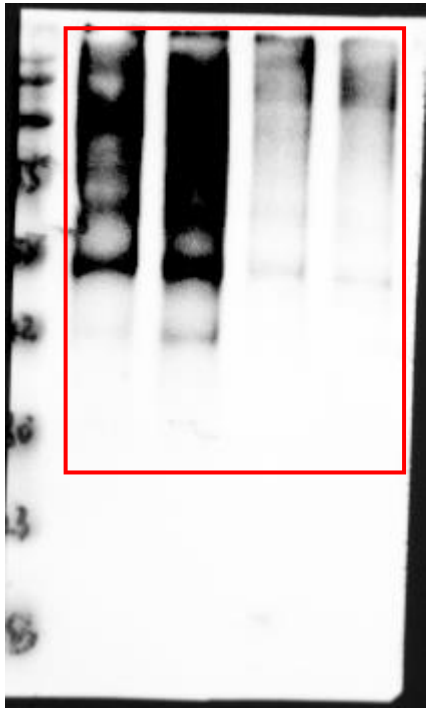

IP: Flag
IB: HA

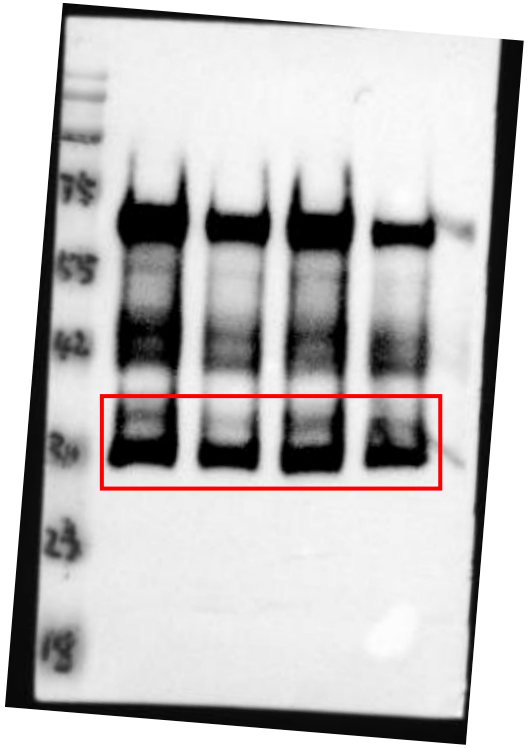

IP: Flag
IB: Flag

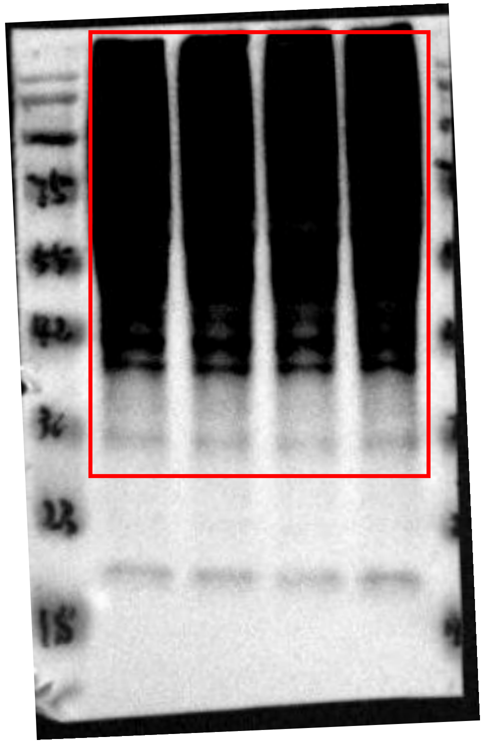

WCL
IB: HA

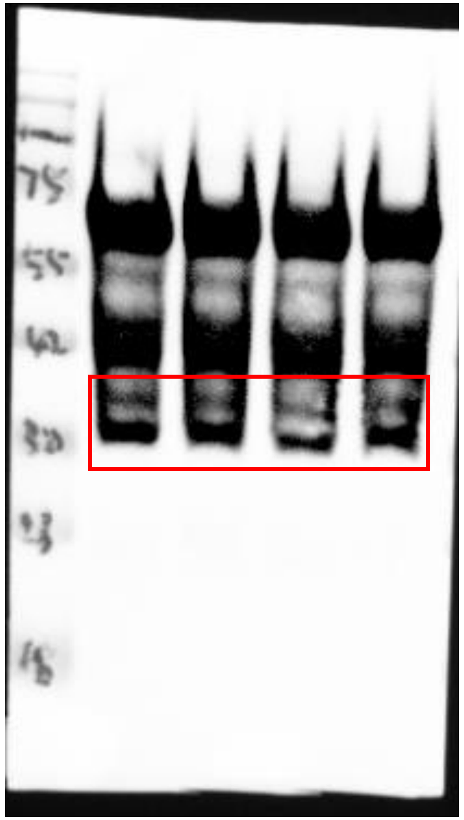

WCL
IB: Flag

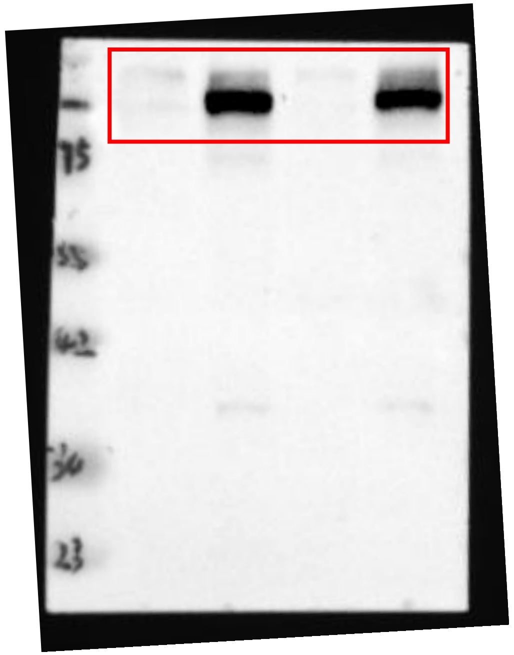

WCL
IB: GFP

Figure 6E

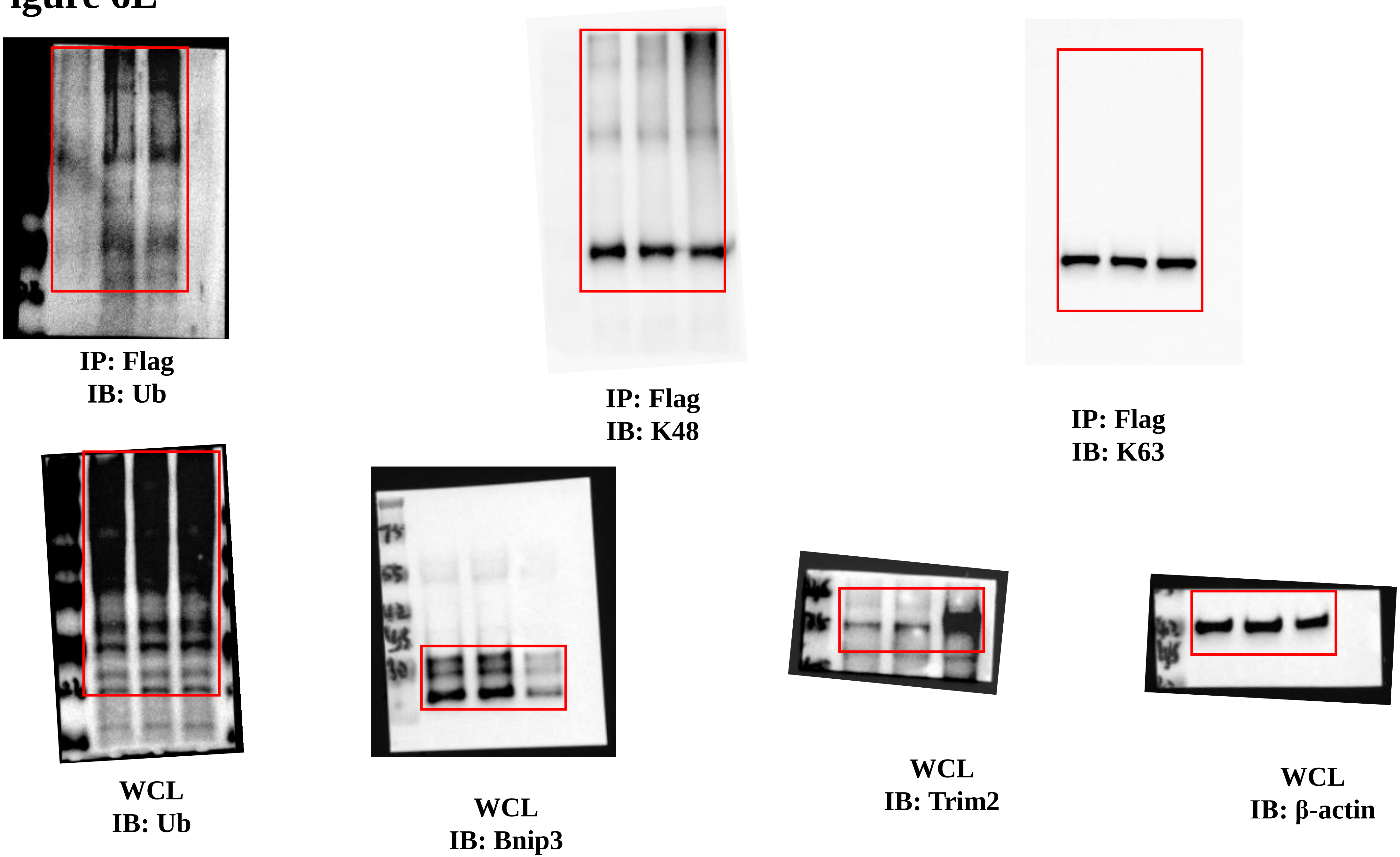
IP: Flag
IB: Ub
IP: Flag
IB: K48
IP: Flag
IB: K63
WCL
IB: Ub
WCL
IB: Bnip3
WCL
IB: Trim2
WCL
IB: β-actin

Figure 6F

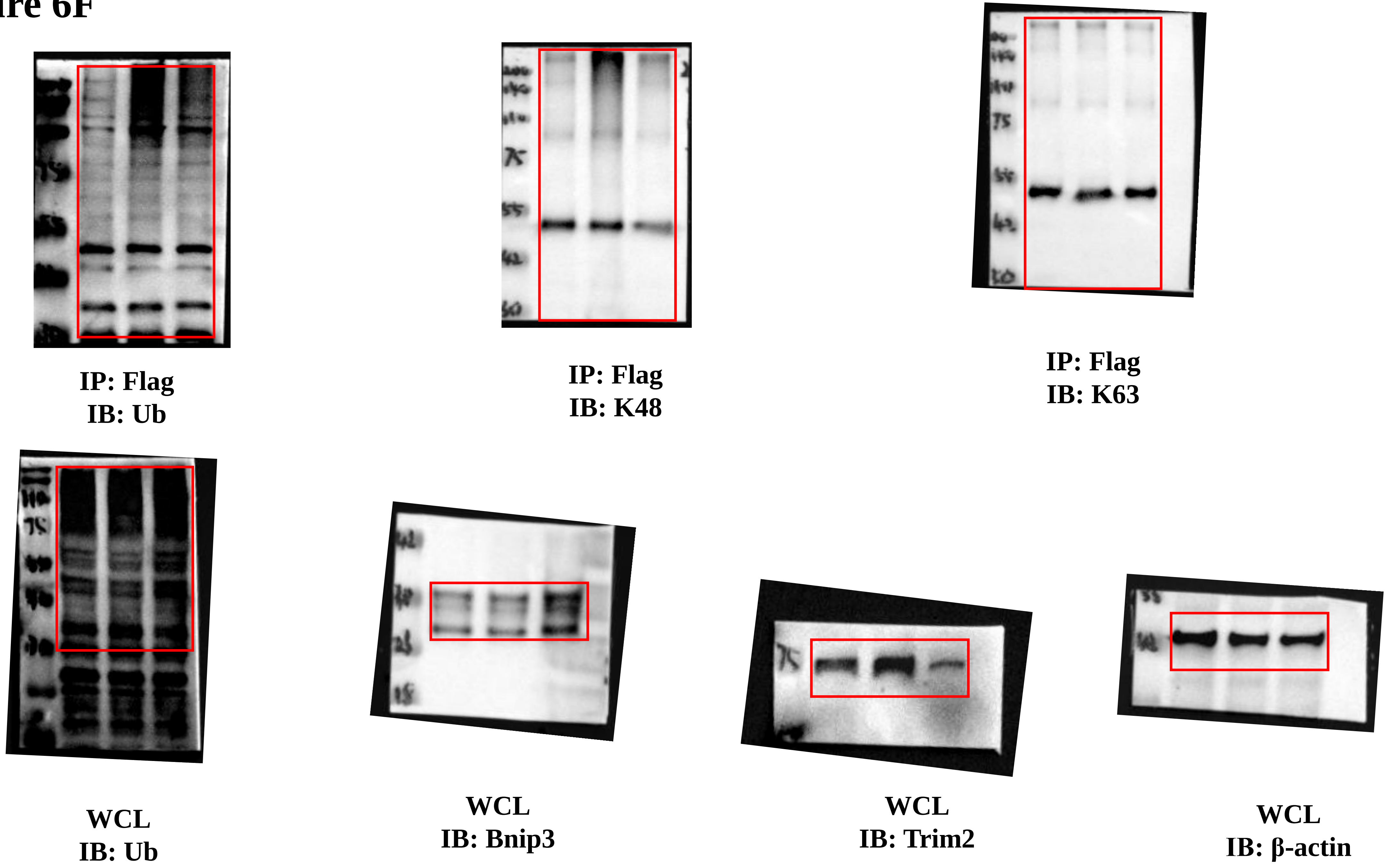
IP: Flag
IB: Ub
IP: Flag
IB: K48
IP: Flag
IB: K63
WCL
IB: Ub
WCL
IB: Bnip3
WCL
IB: Trim2
WCL
IB: β-actin

Figure 6H

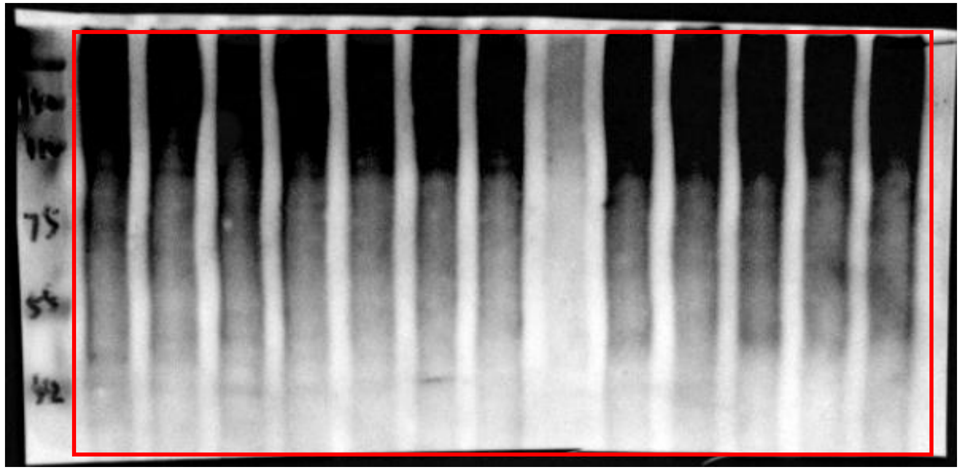

IP: Flag
IB: HA

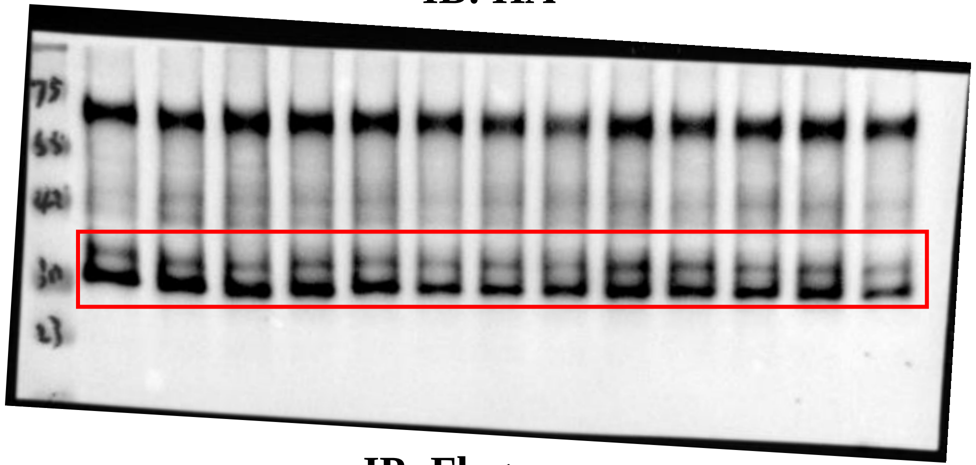

IP: Flag
IB: Flag

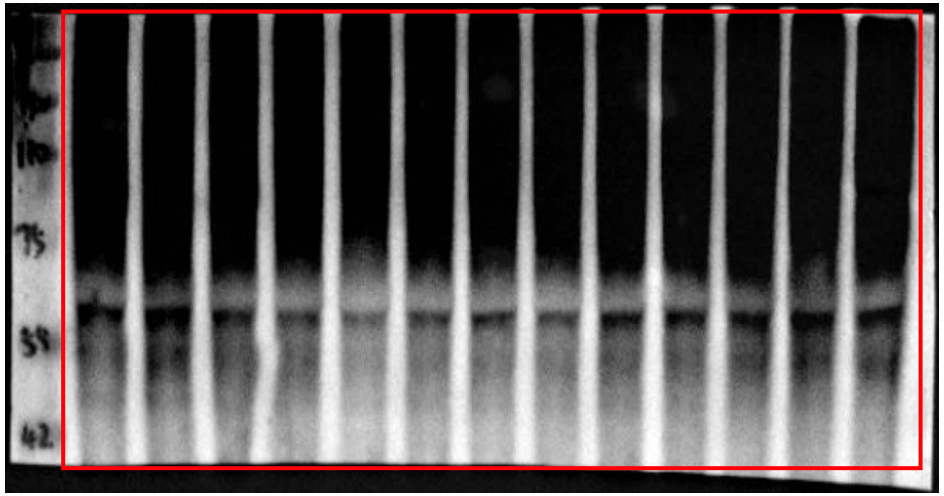

WCL
IB: HA

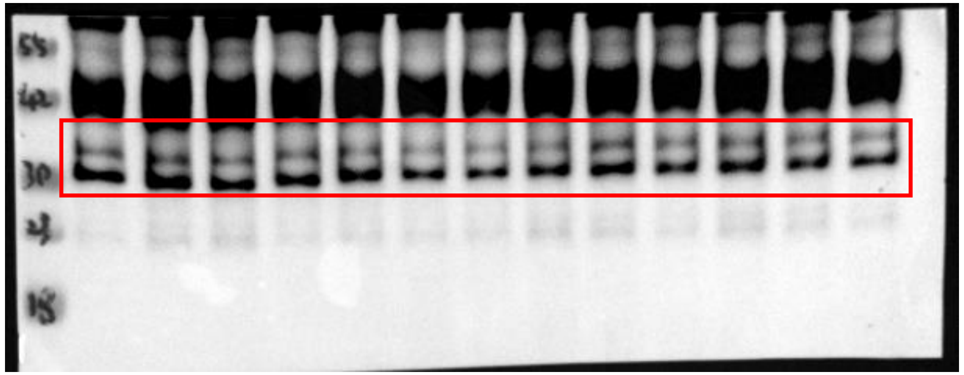

WCL
IB: Flag

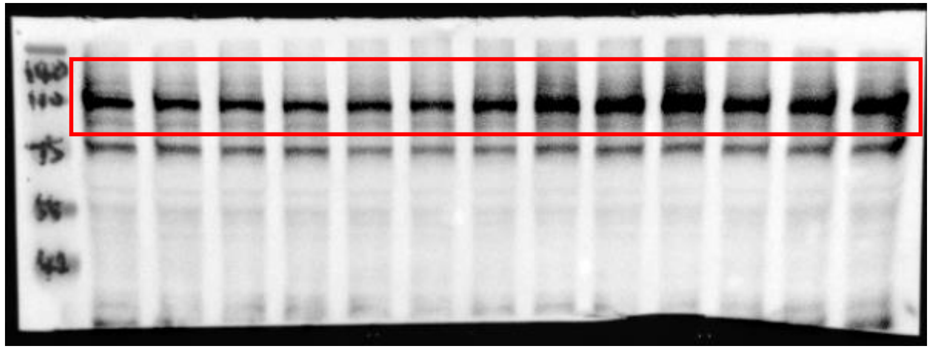

WCL
IB: GFP

Figure 7A

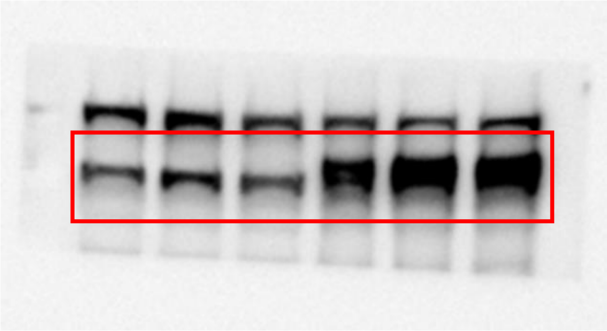

Caco-2 cell
IB: Trim2

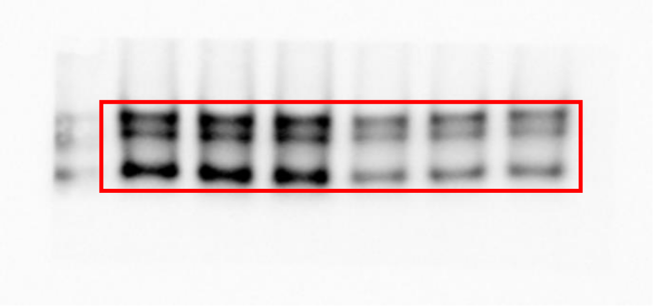

Caco-2 cell
IB: Bnip3

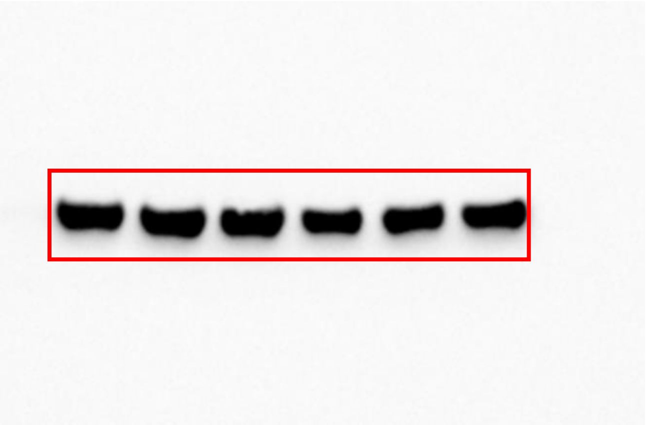

Caco-2 cell
IB: β-actin

Figure 7C

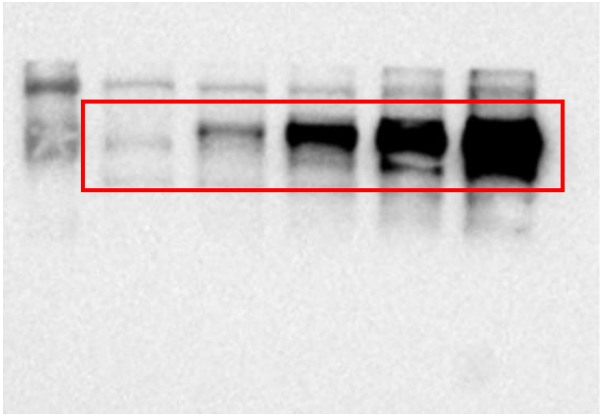

293T cell
IB: Trim2

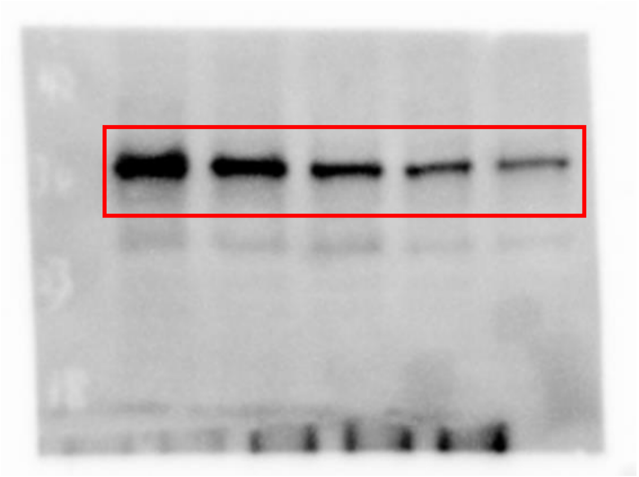

293T cell
IB: Bnip3

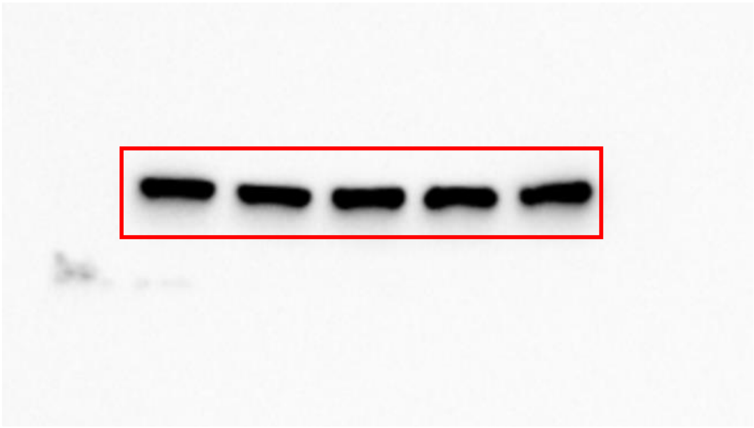

293T cell
IB: β-actin

Figure 7E

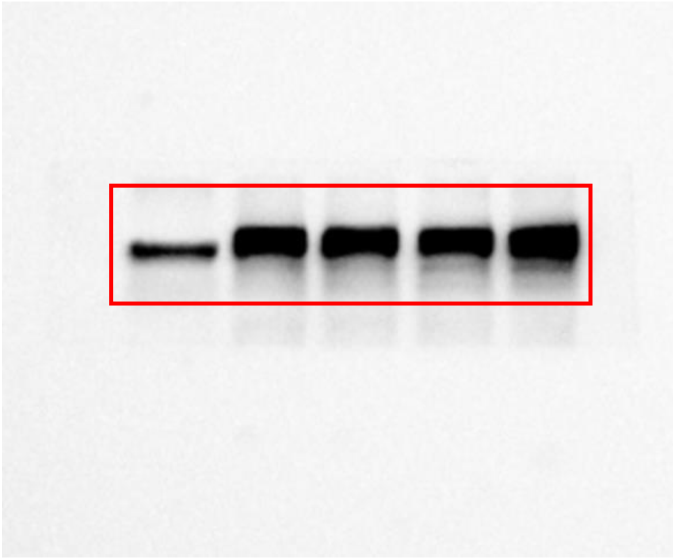

Caco-2 cell
IB: Trim2

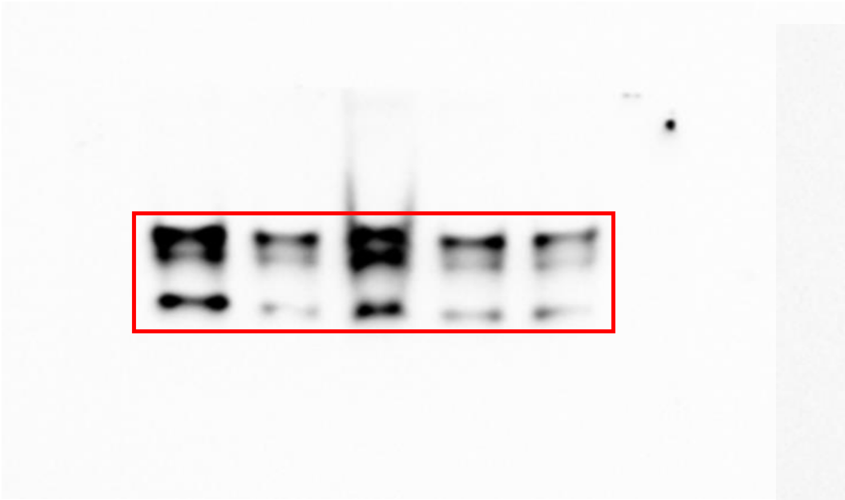

Caco-2 cell
IB: Bnip3

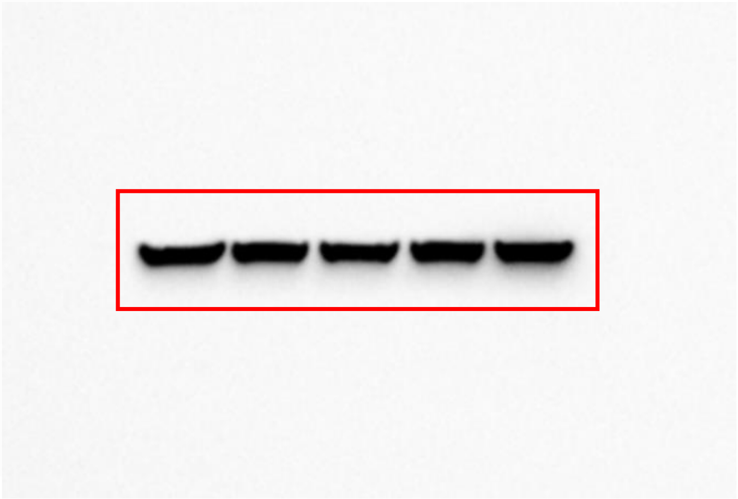

Caco-2 cell
IB: β-actin

Figure 7F

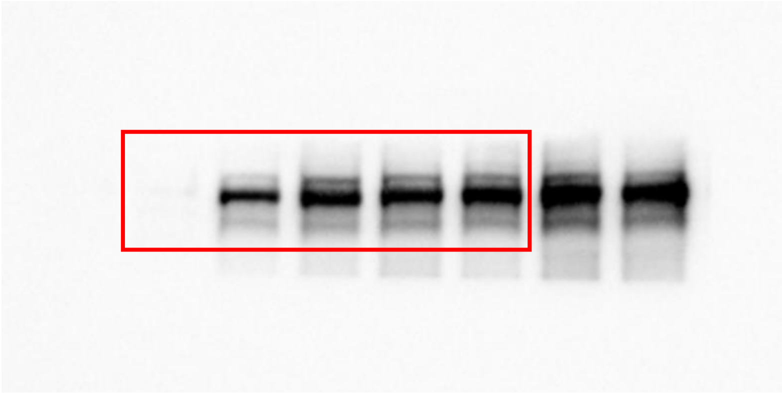

293T cell
IB: Trim2

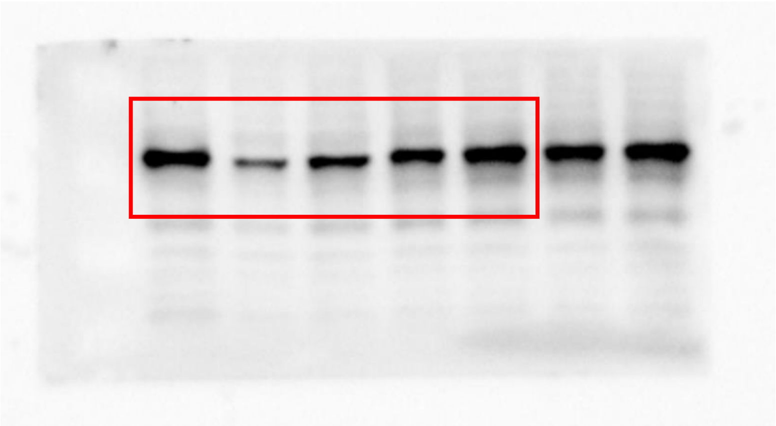

293T cell
IB: Bnip3

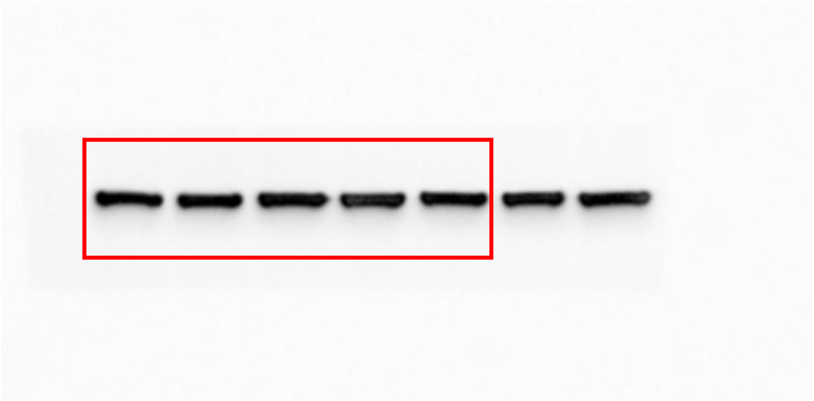

293T cell
IB: β-actin

Figure 7G

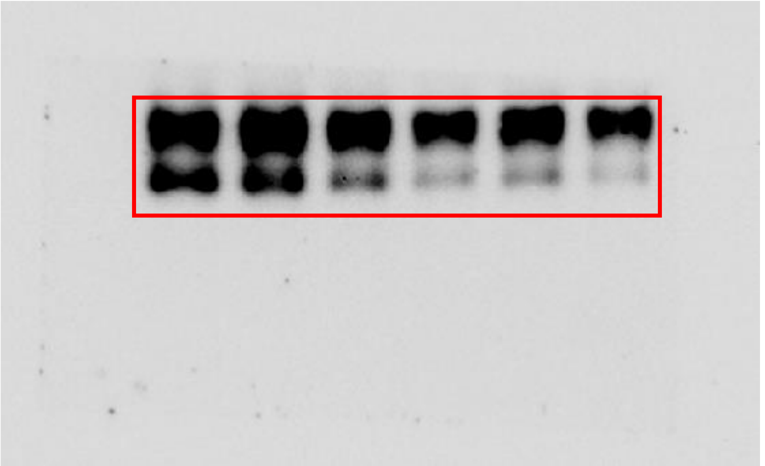

Caco-2 cell
IB: Bnip3

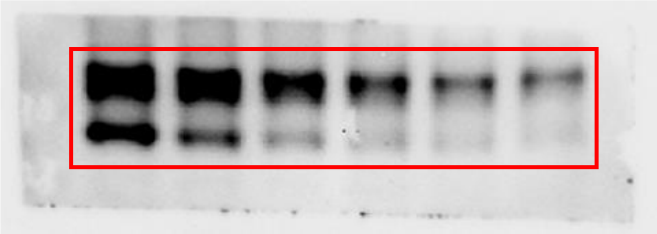

Caco-2 cell
IB: Bnip3

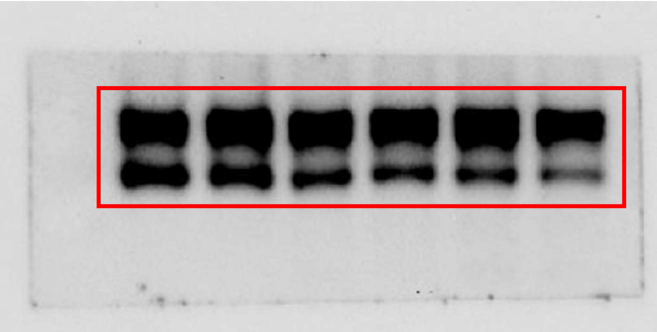

Caco-2 cell
IB: Bnip3

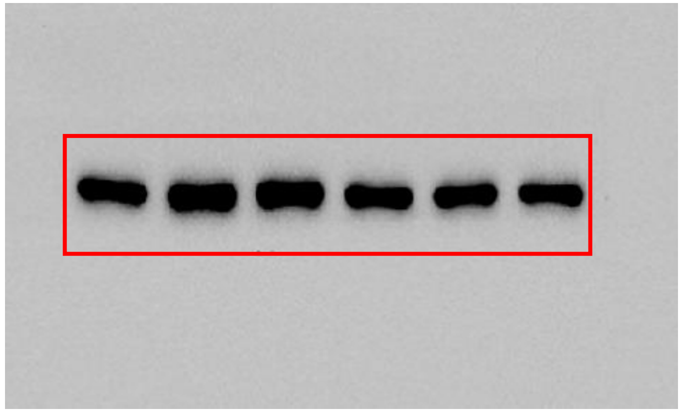

Caco-2 cell
IB: β-actin

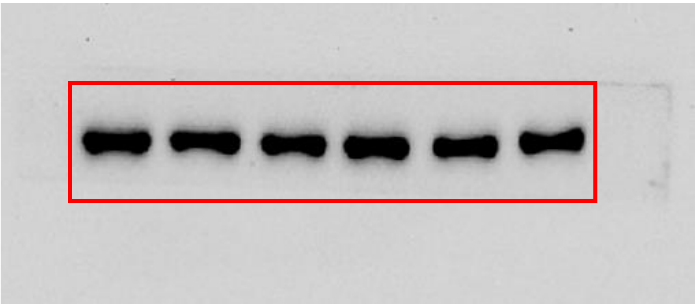

Caco-2 cell
IB: β-actin

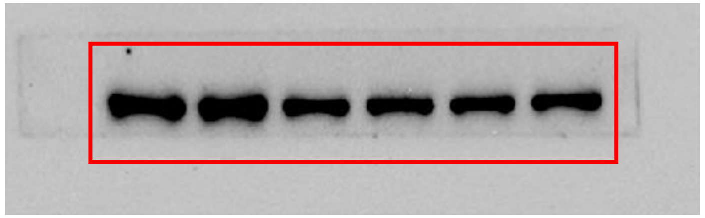

Caco-2 cell
IB: β-actin

Figure 7H

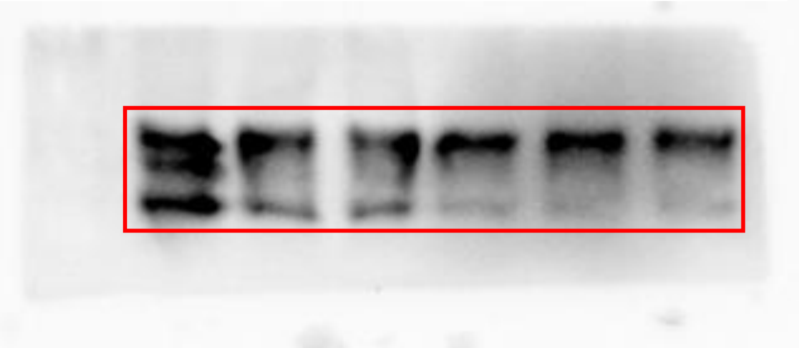

Caco-2 cell
IB: Bnip3

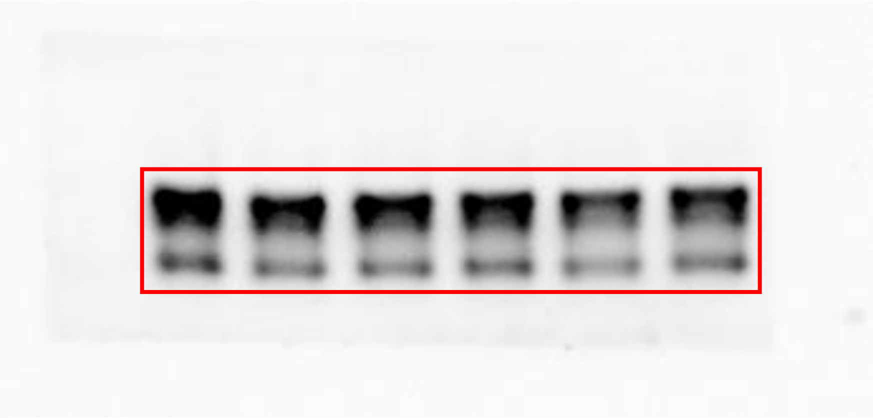

Caco-2 cell
IB: Bnip3

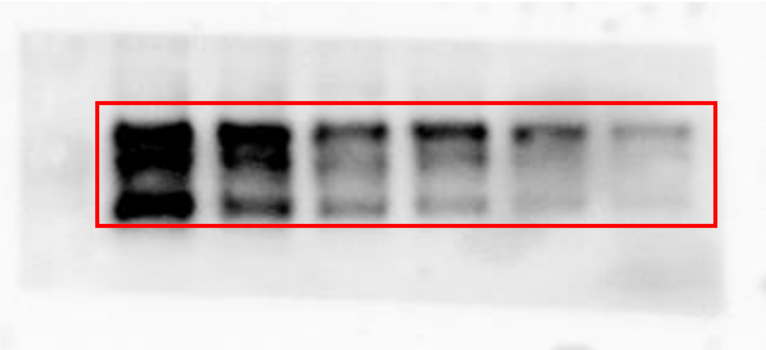

Caco-2 cell
IB: Bnip3

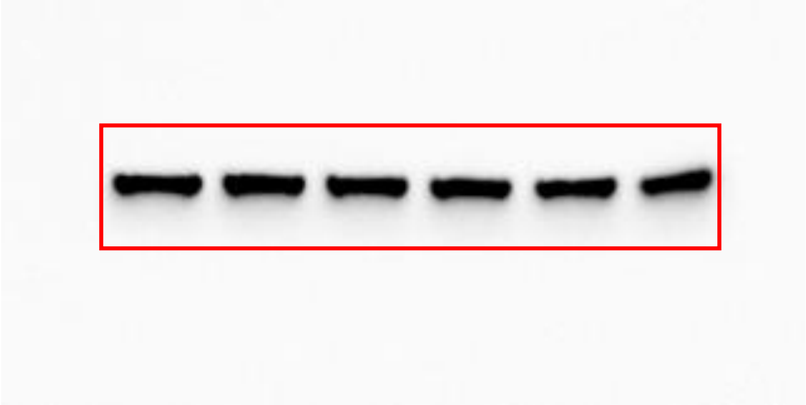

Caco-2 cell
IB: β-actin

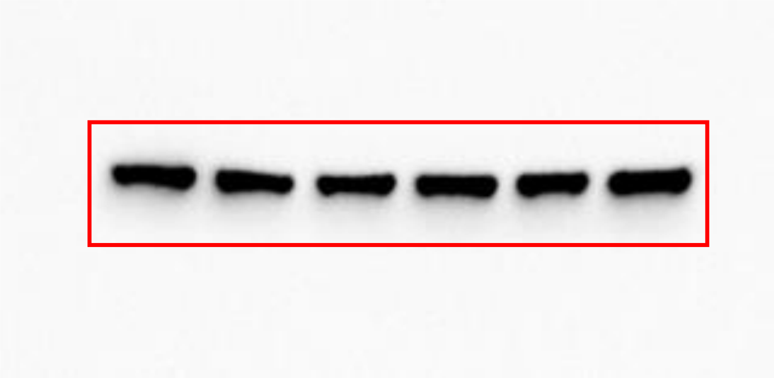

Caco-2 cell
IB: β-actin

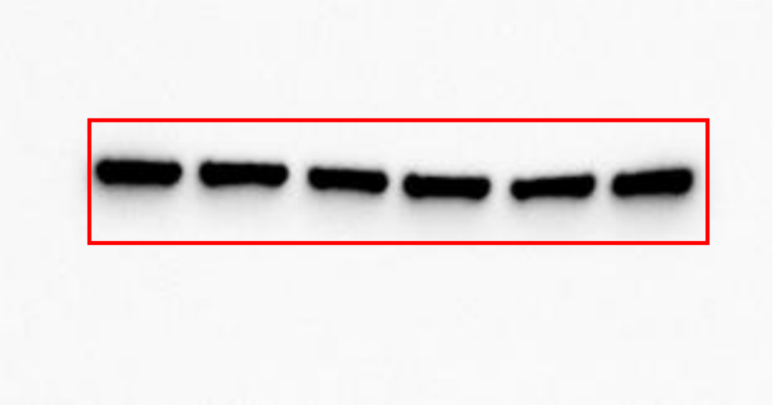

Caco-2 cell
IB: β-actin

Figure 7I

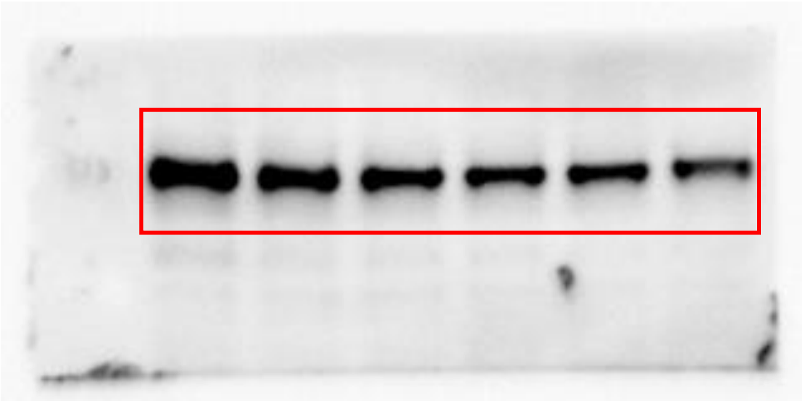

293T cell
IB: Flag

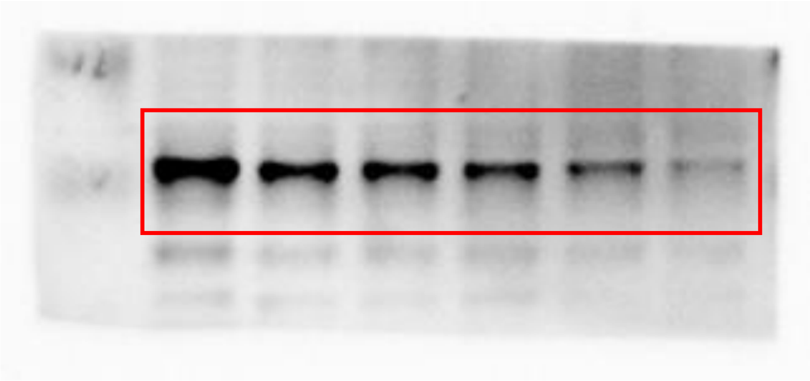

293T cell
IB: Flag

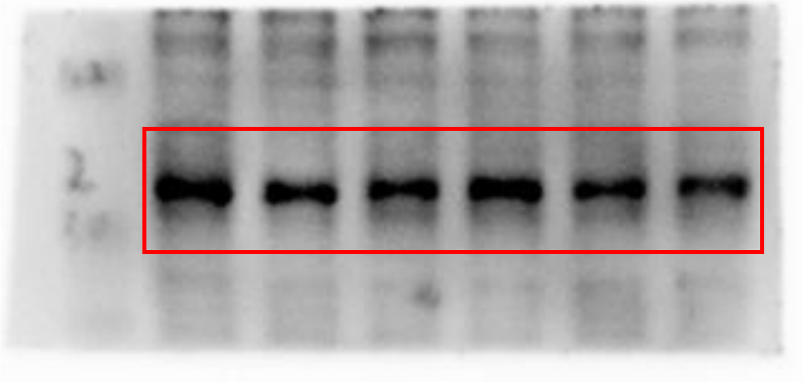

293T cell
IB: Flag

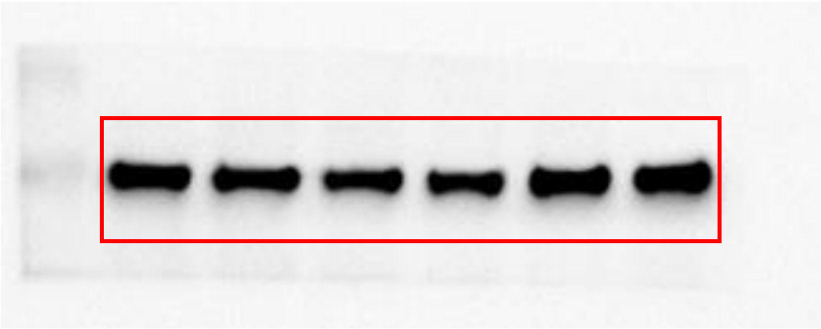

293T cell
IB: β-actin

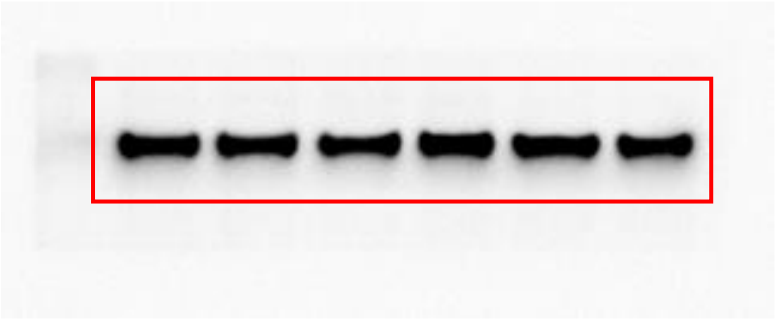

293T cell
IB: β-actin

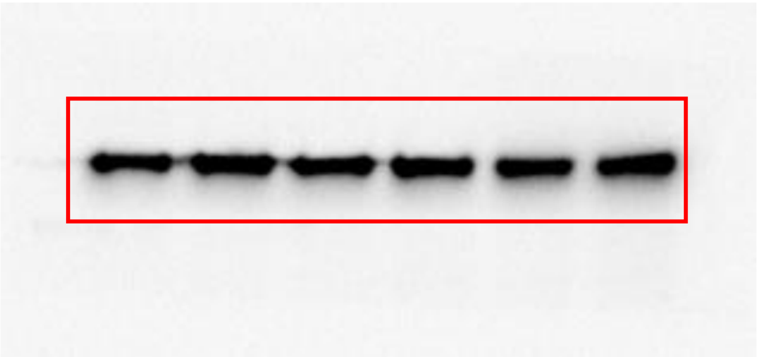

293T cell
IB: β-actin

Figure 8E

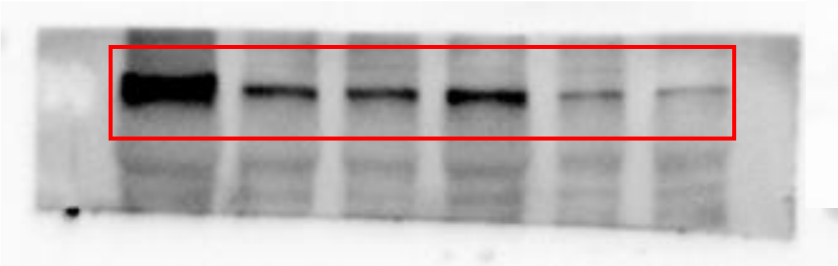

Caco-2 cell
IB: TRIM2

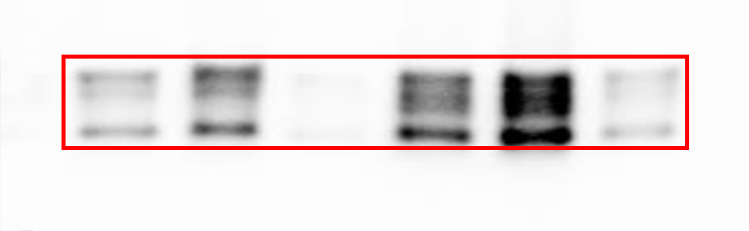

Caco-2 cell
IB: BNIP3

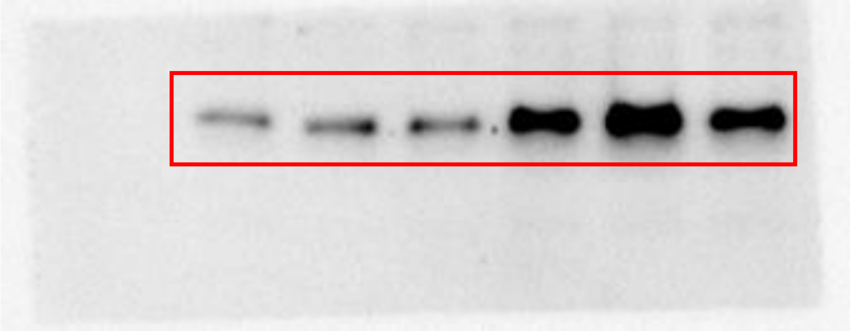

Caco-2 cell
IB: BAX

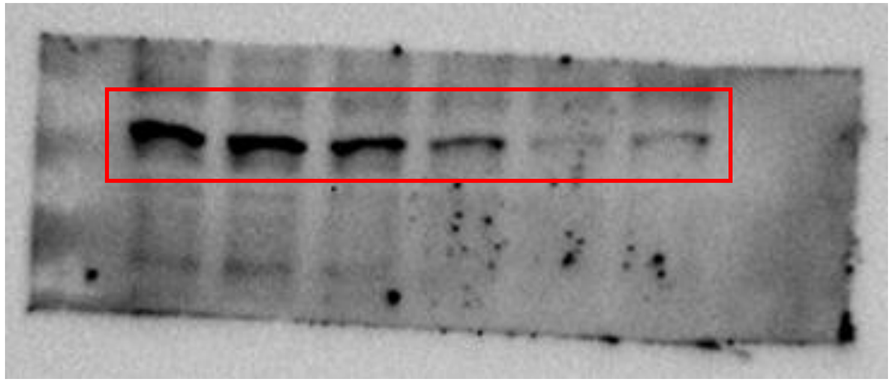

Caco-2 cell
IB: BCL-2

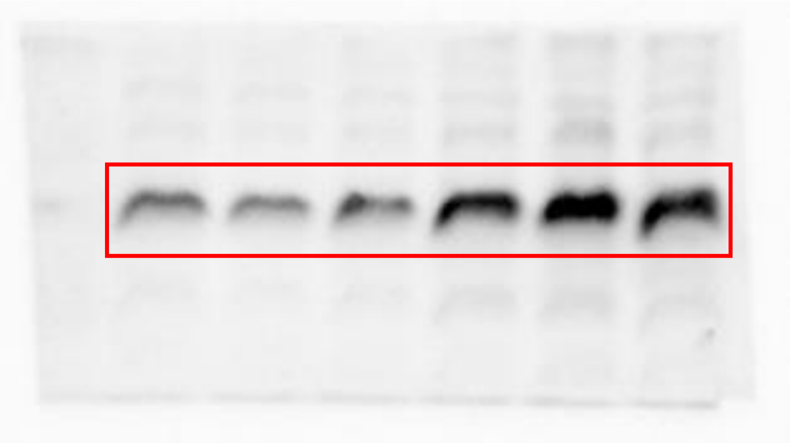

Caco-2 cell
IB: BAD

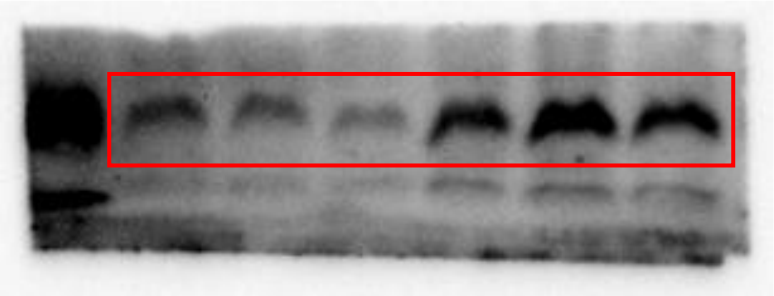

Caco-2 cell
IB: cleaved CASP3

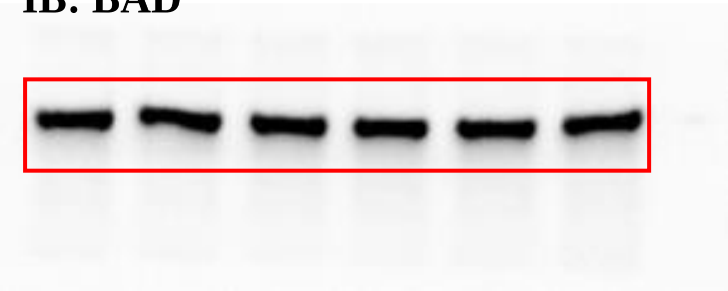

Caco-2 cell
IB: β-actin

Figure 8I

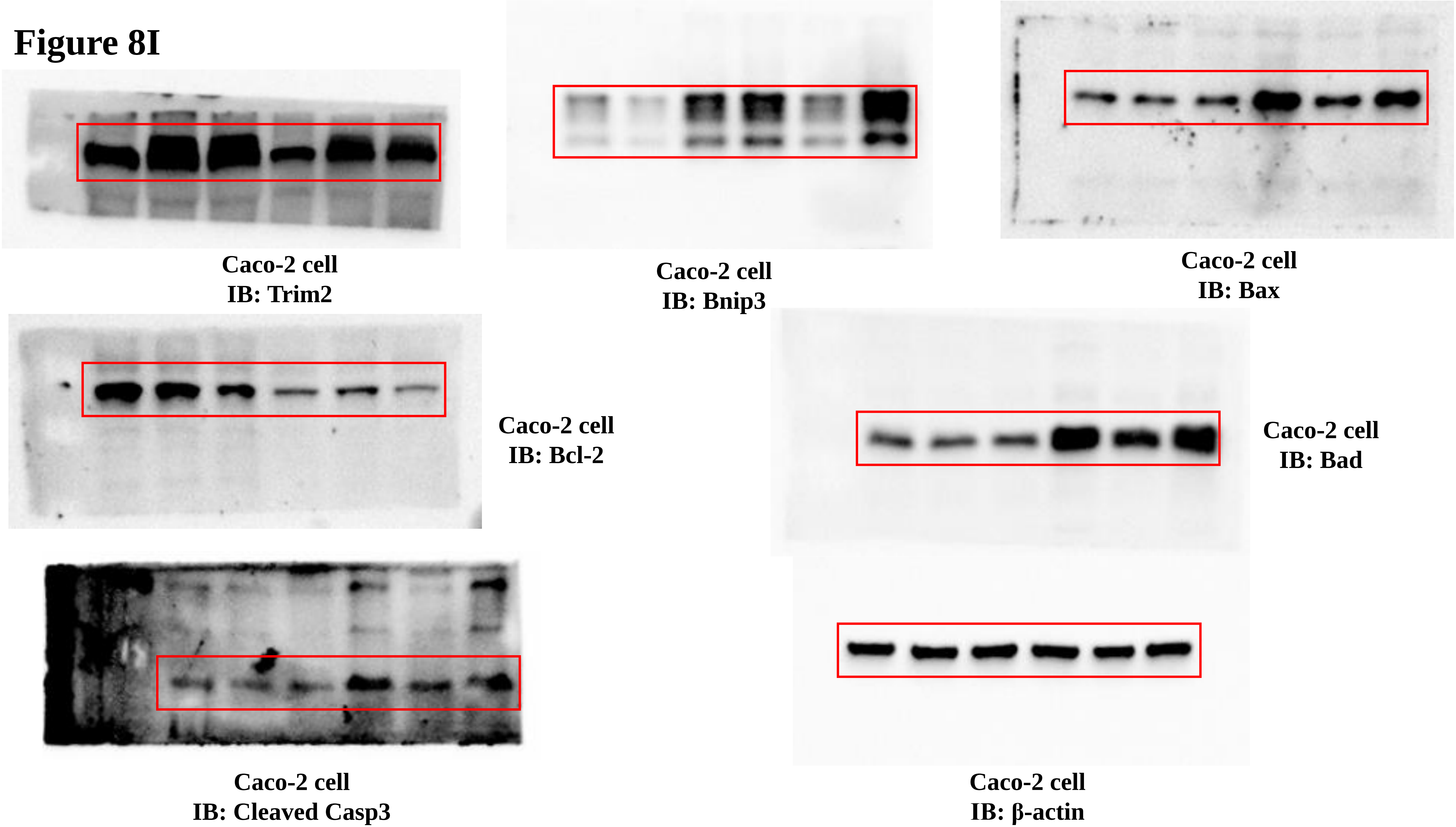
Caco-2 cell
IB: Trim2
Caco-2 cell
IB: Bnip3
Caco-2 cell
IB: Bax
Caco-2 cell
IB: Bcl-2
Caco-2 cell
IB: Bad
Caco-2 cell
IB: Cleaved Casp3
Caco-2 cell
IB: β-actin

Figure S2C

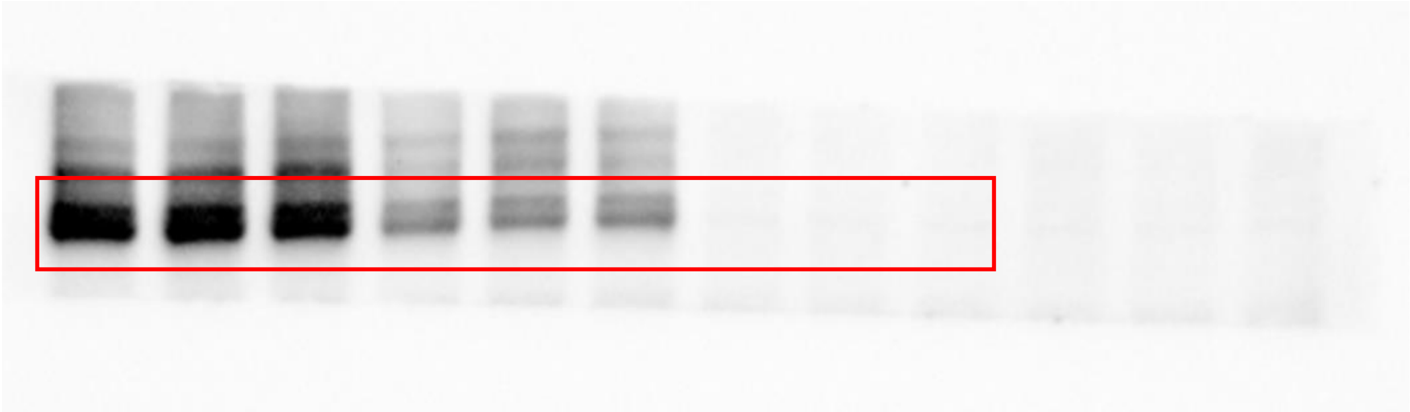

IB: Trim2

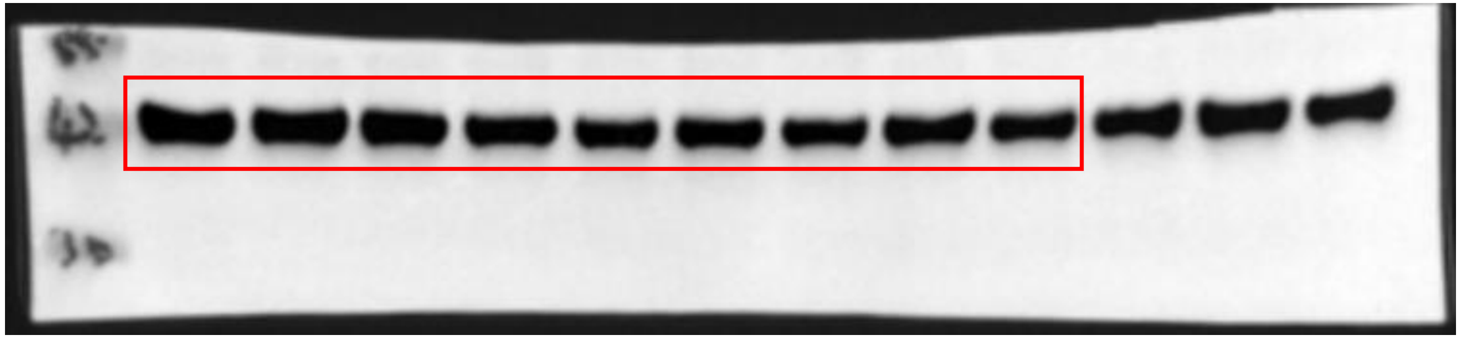

IB: β-actin

Figure S3F

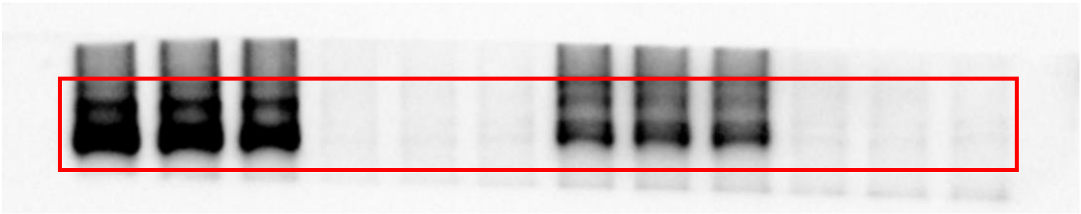

IB: Trim2

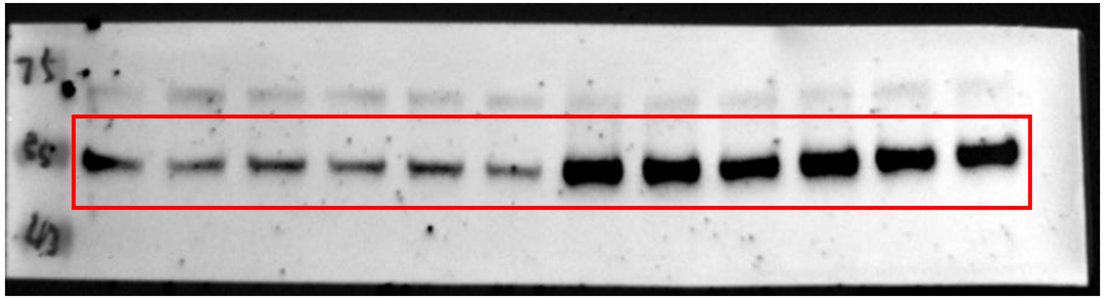

IB: Beclin-1

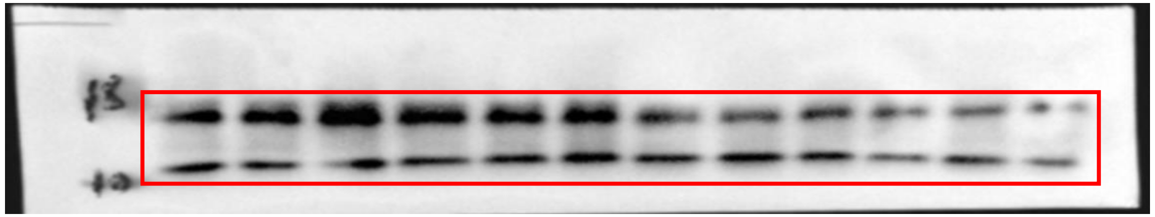

IB: LC3

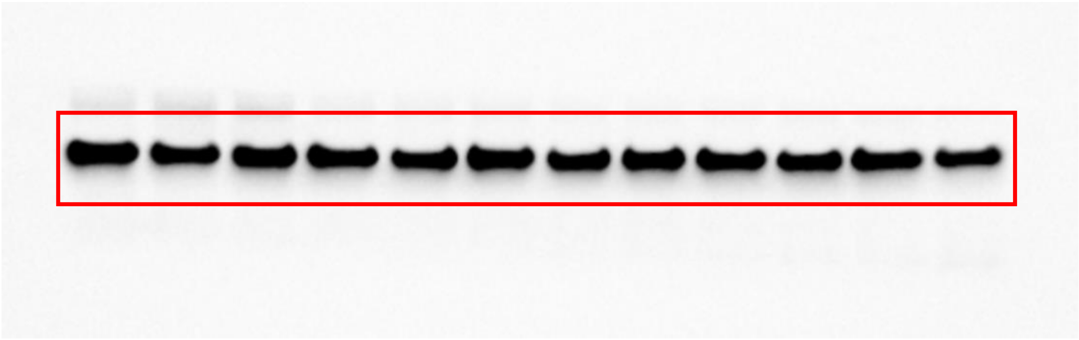

IB: β-actin

Figure S3G

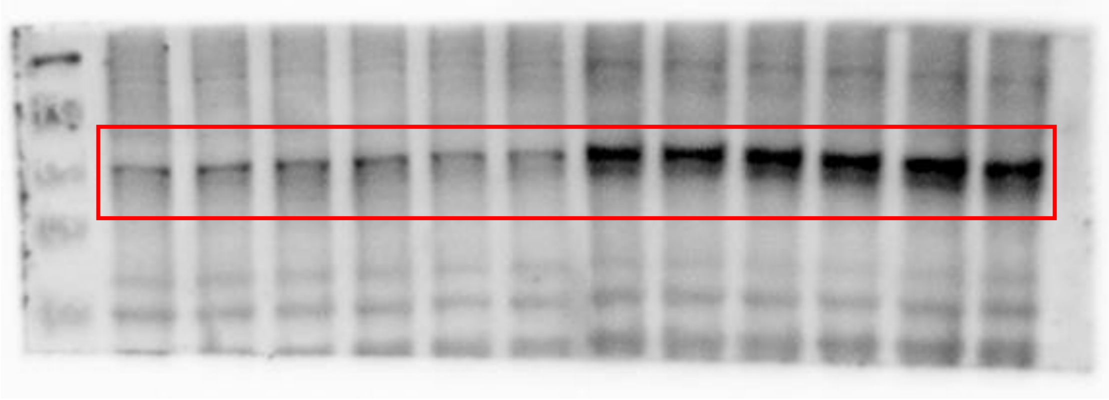

IB: p-Ripk3

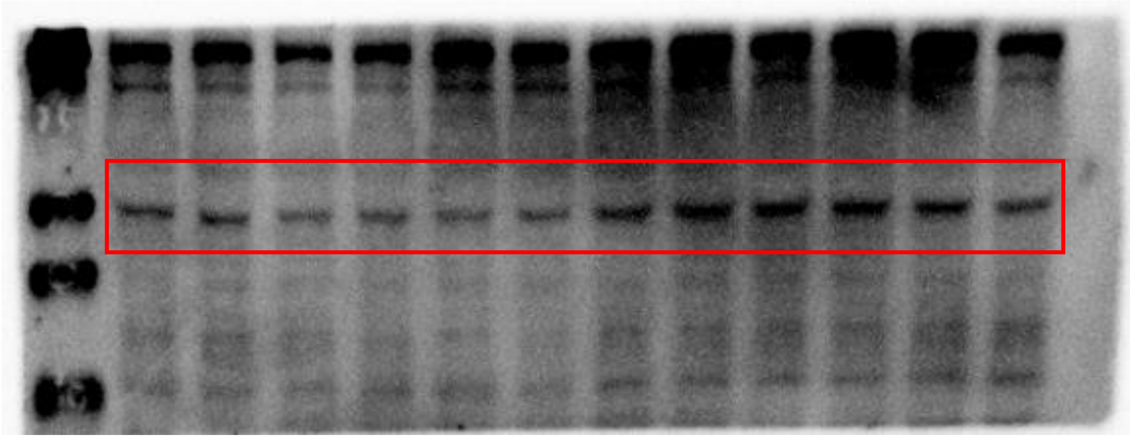

IB: Ripk3

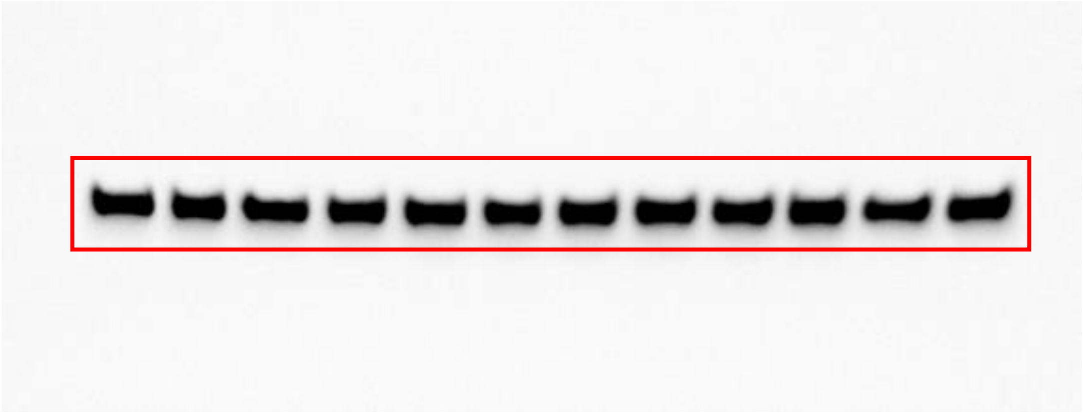

IB: β-actin

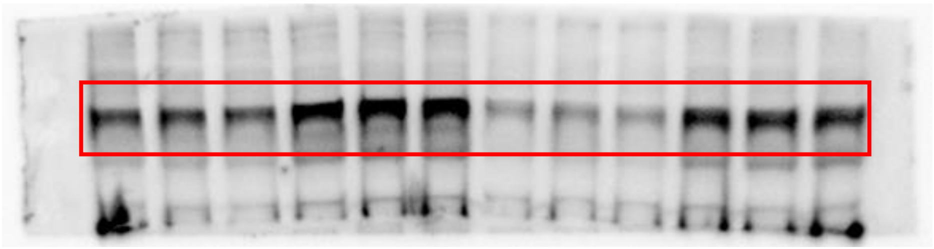

IECs
IB: Trim2

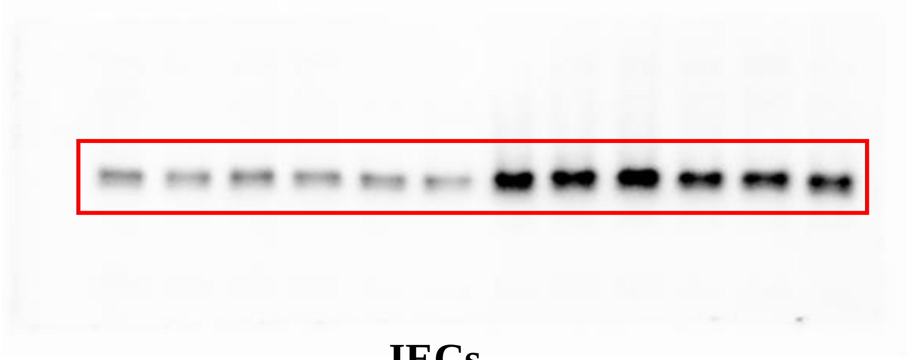

IECs
IB: Bax

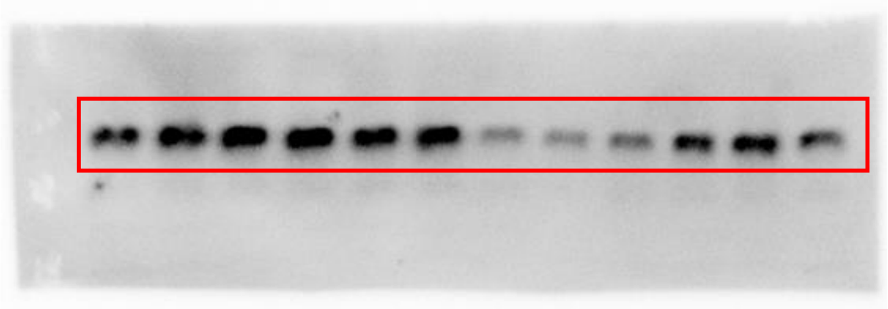

IECs
IB: Bcl-2

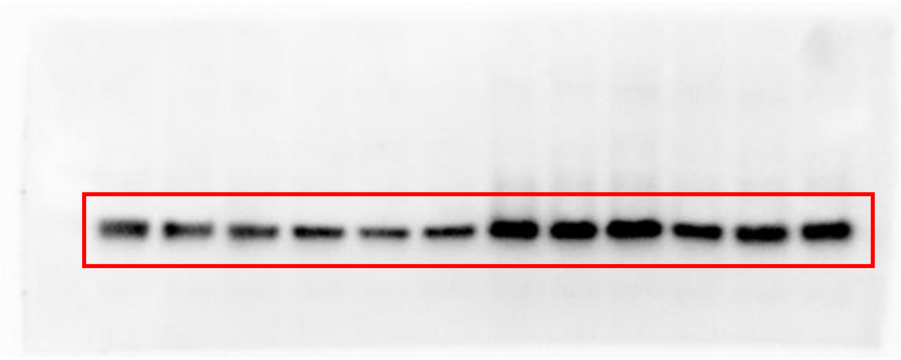

IECs
IB: Bad

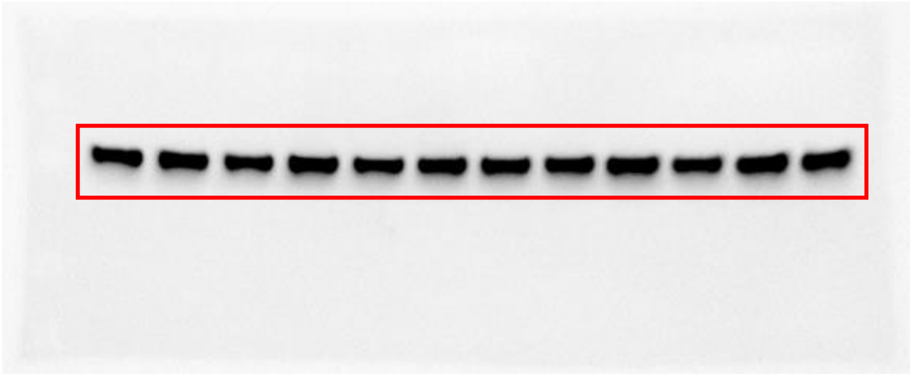

IECs
IB: β-actin

Figure S8

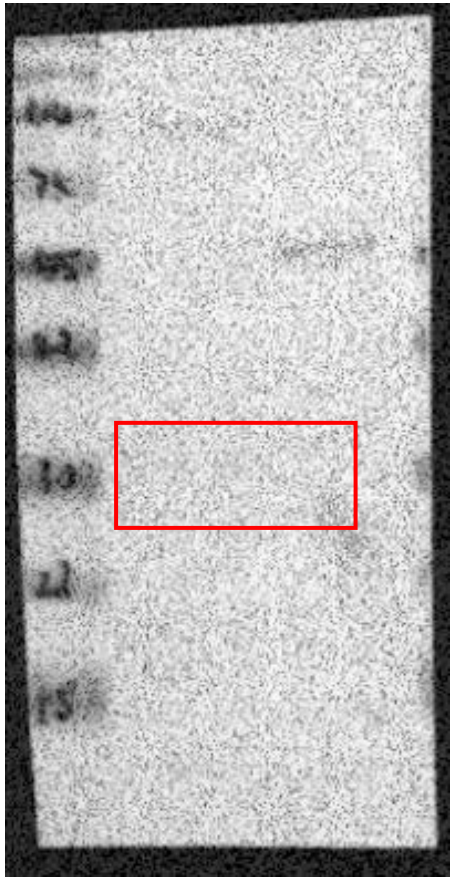

GST pull-down
IB: Flag

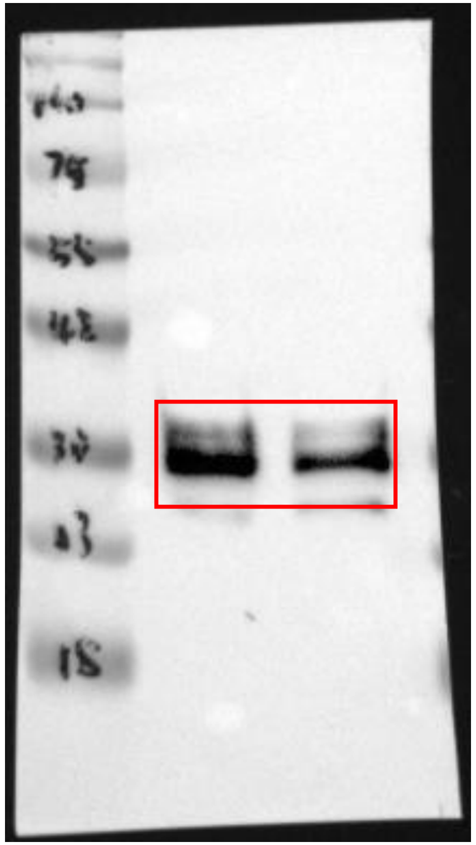

Input
IB: Flag

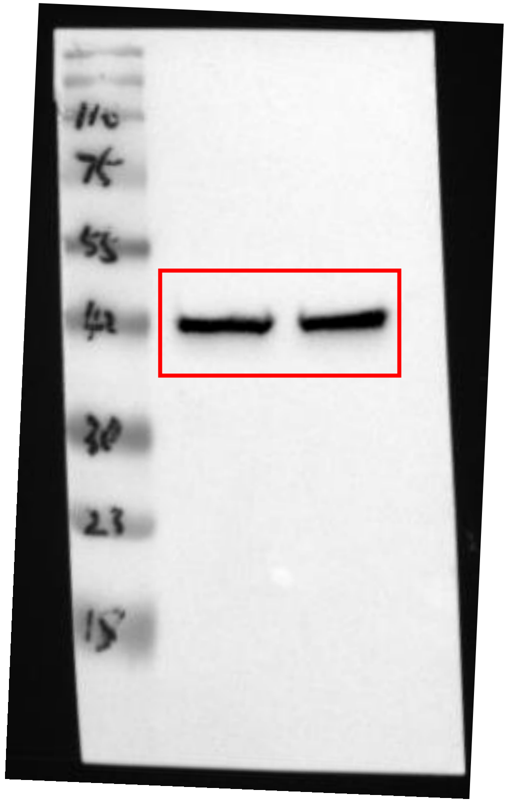

Input
IB: β-actin

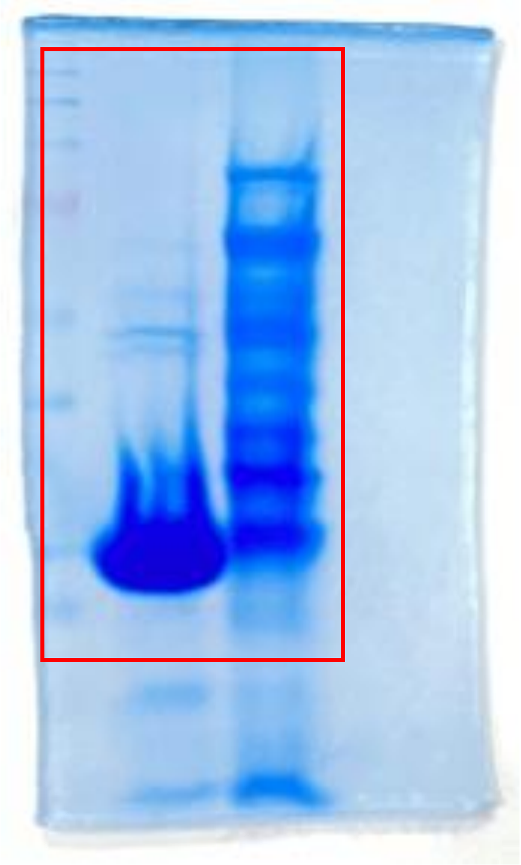

GST pull-down (1-163)
Coomassie

Figure S9

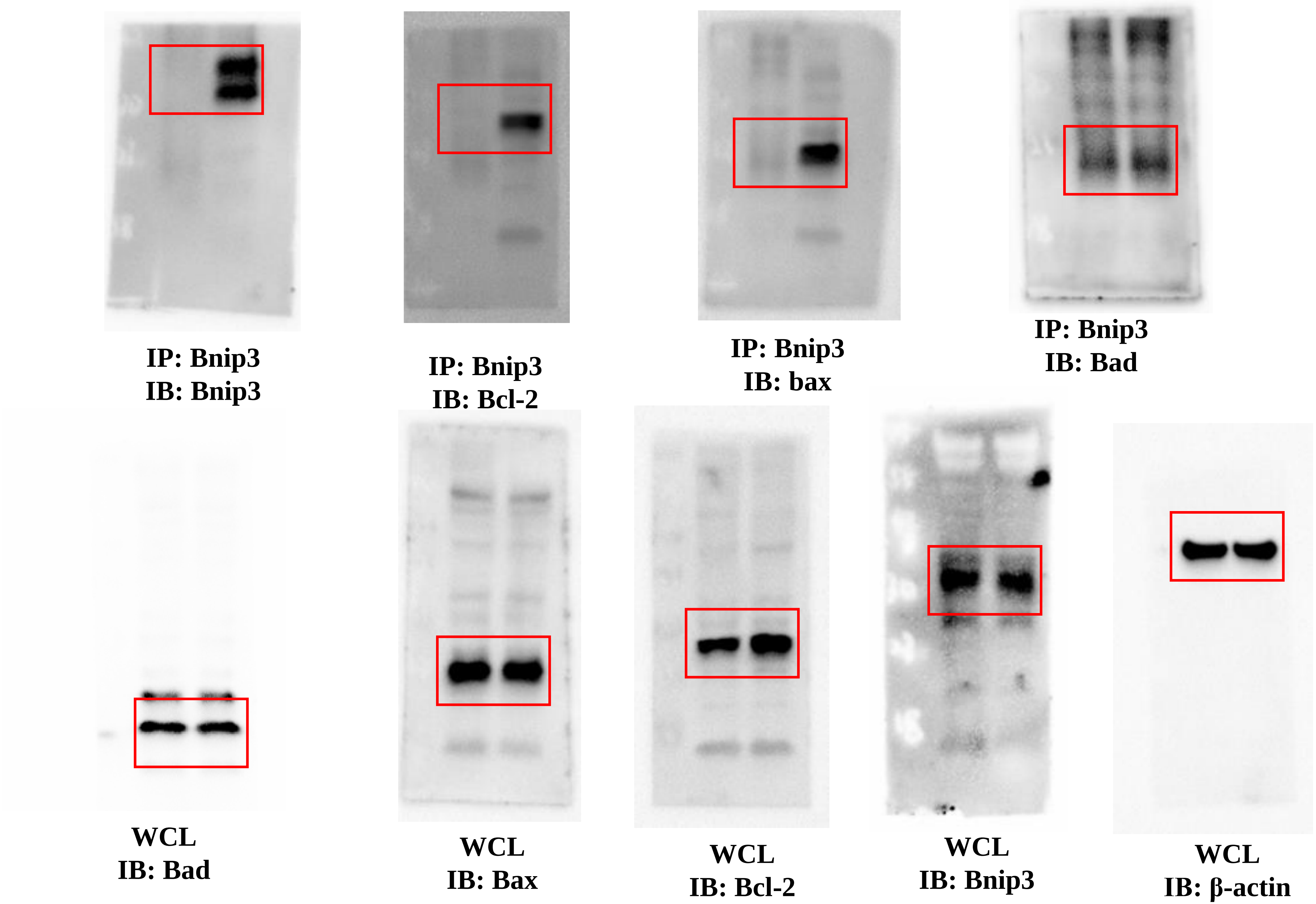
IP: Bnip3
IB: Bnip3
IP: Bnip3
IB: Bcl-2
IP: Bnip3
IB: bax
IP: Bnip3
IB: Bad
WCL
IB: Bad
WCL
IB: Bax
WCL
IB: Bcl-2
WCL
IB: Bnip3
WCL
IB: β-actin

Figure S10

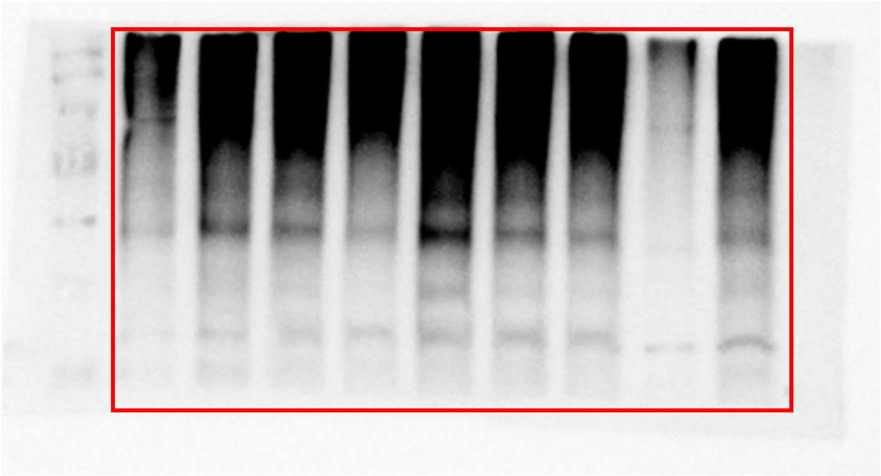

IP: Flag
IB: HA

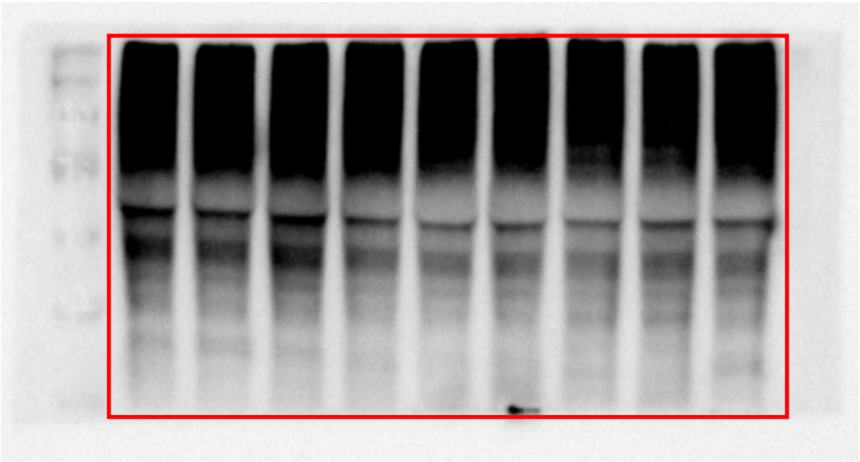

WCL
IB: HA

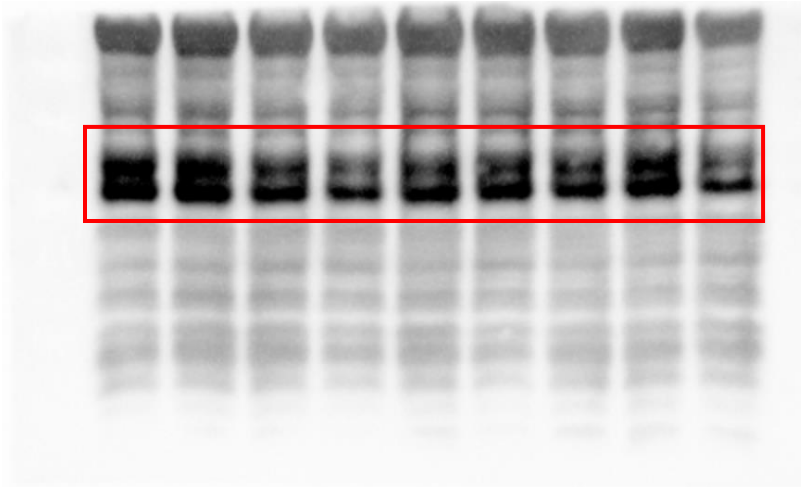

IP: Flag
IB: Flag

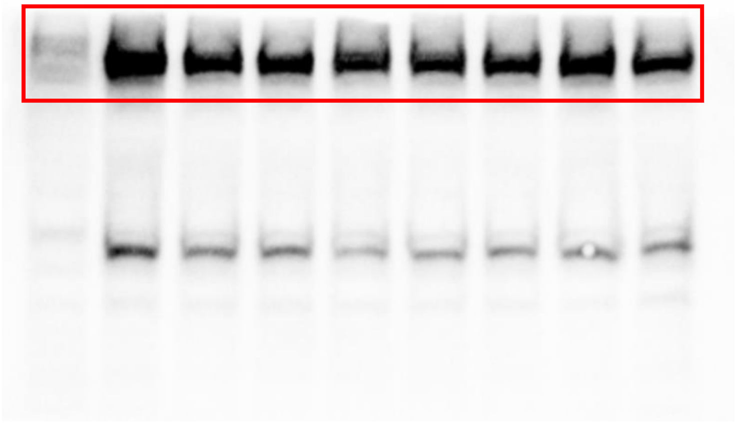

WCL
IB: GFP

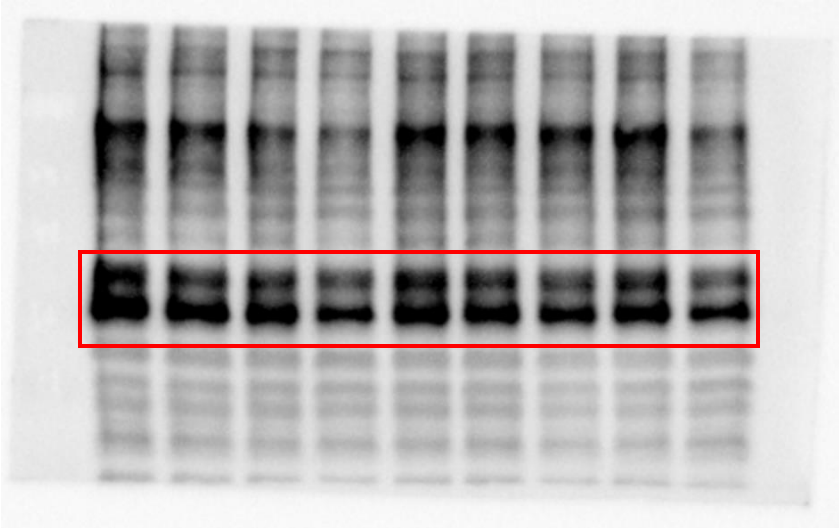

WCL
IB: Flag

Figure S11C

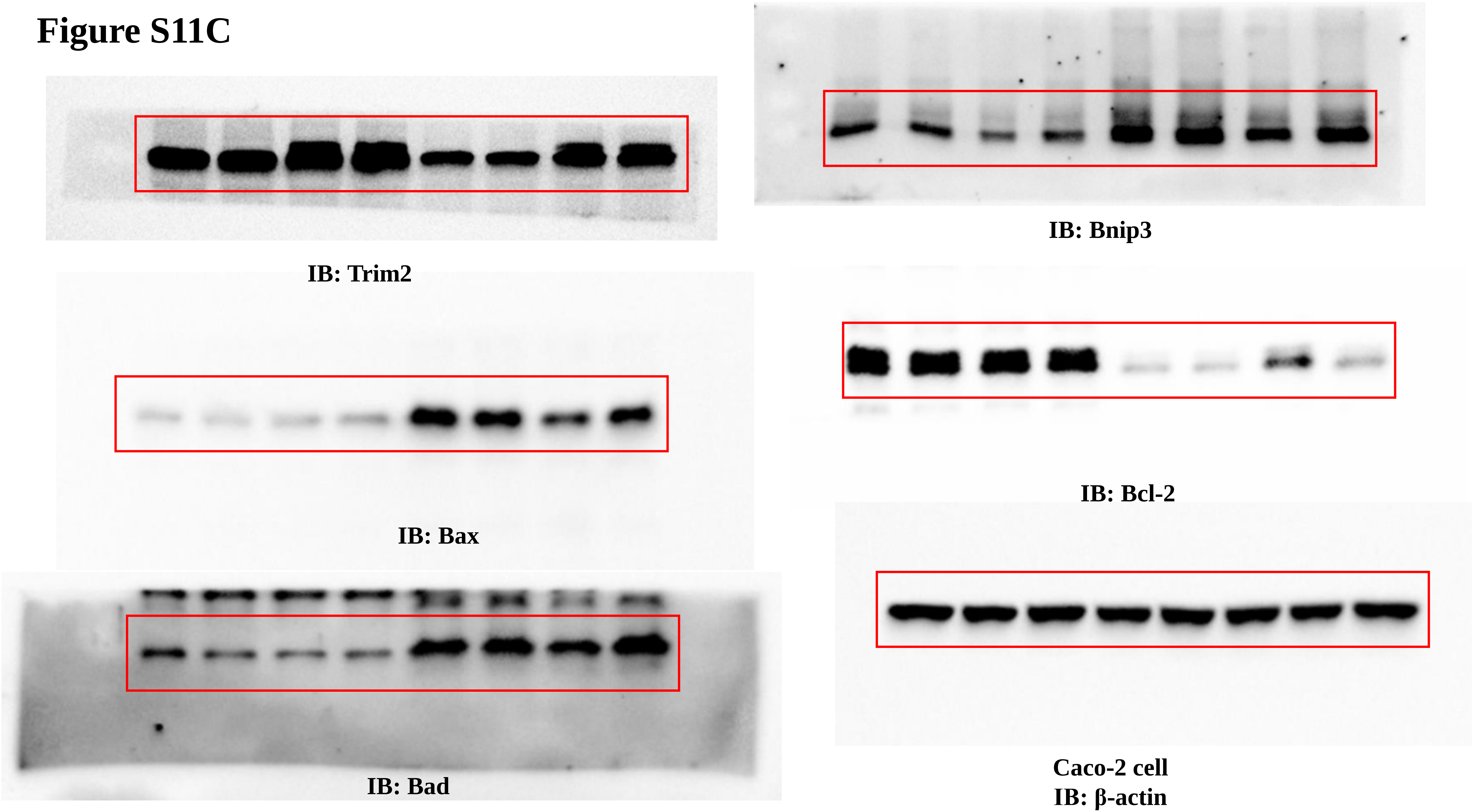

Supplement: Supplementary file 2 — Supplementary Information [file 42003_2025_8708_MOESM2_ESM.pdf]
