# Supplementary material for: TRIM2 inhibits apoptosis by ubiquitinating BNIP3 to protect the intestine against ischemia-reperfusion injury in mice
Source: Commun Biol. 2025 Aug 29;8:1308. doi: 10.1038/s42003-025-08708-2 (PMC12397258; doi:10.1038/s42003-025-08708-2)
Supplement: Supplementary file 3 — Description of Additional Supplementary files [file 42003_2025_8708_MOESM3_ESM.pdf]

Description of Additional Supplementary files

File name: Supplementary Data 1

Description: qRT-PCR primers used in this study

File name: Supplementary Data 2

Description: The source data behind the graphs in the paper
