# Supplementary material for: TRIM2 inhibits apoptosis by ubiquitinating BNIP3 to protect the intestine against ischemia-reperfusion injury in mice
Source: Commun Biol. 2025 Aug 29;8:1308. doi: 10.1038/s42003-025-08708-2 (PMC12397258; doi:10.1038/s42003-025-08708-2)
Supplement: Supplementary file 4 — Supplementary Data 1 [file 42003_2025_8708_MOESM4_ESM.docx]

**qRT-PCR primers used in this study**

| Gene | Forward primer | Reverse primer |
| --- | --- | --- |
| mTRIM1 | CCGGGAACAATATGGGTGAGA | AGGCTGTGAGCACAAGGAAG |
| mTRIM2 | TGGGCTCATTTGTCTGAGGG | CTGCACGCTGCTGGGTT |
| mTRIM3 | CCATGAAGGCAAGACAATGGA | TGTGCTGCTCCACCACATC |
| mTRIM5 | AAAACCATCGCCAGGGAACA | AGGGTCACCTGAACCCAGTA |
| mTRIM6 | CTCCTTGGCTGCAAGTCCTC | AGCTTGCTTAGAAAAAGAATCCAAT |
| mTRIM7 | GACTCCAGCGGTGCCTG | GAATCCAGCAACTCCTTGGC |
| mTRIM8 | GCTGCCCGCAGCACAAC | CTGCTTCATGAGCATCTTCCGTATC |
| mTRIM9 | CAACCACCCTGATCCAGCAT | TCCCTCCTTCAGTTATTGCCC |
| mTRIM10 | CGGTTCAGCACCCTGATTGA | AGAGTGCTTCTGATATCCGTCA |
| mTRIM11 | TGGCAGAAATGGCACGACG | CTTCTCCAGCCTCCCCTTGAG |
| mTRIM15 | CCTGAGCGAGACCTACTGTG | ACTCCTGAGACGATCCCTGT |
| mTRIM16 | CGGTGTATGCAGTTGGACCT | CCTCTGACACCGACACCAAA |
| mTRIM17 | GCAAGGCTCCTTCCTTGAGC | CACAGGCAGTCATCACAGGA |
| mTRIM18 | AAGAGCAGTCTGTACTTGCCC | CTGATCAGCTATCGGCTTCC |
| mTRIM19 | ATAGCAGCAGTGAGTCCAGC | GCTGGCTAATTTTCTGGGTTTC |
| mTRIM20 | TGGAAACCTTCATTCATTGGGA | CACGGTAACTGCAGGAAGAAG |
| mTRIM21 | AGGCTCCTGTCATTCAGATTCCA | GAACTGCCCCCATTCTTCCC |
| mTRIM23 | AGCACCAAGGTCACAAGCATT | TGAAGGTCCGTATGCAGTGAG |
| mTRIM24 | ATCTCTCATCAGCATCCGCC | TTGCAAGGGGTTGGGCTTTA |
| mTRIM25 | GGAGACCAGACACAGTCCAC | CGGGAGCAACAGGGGTTT |
| mTRIM26 | CTGCACTACACAGGACACCA | GTGAGGGATGGCTTTCCCAA |
| mTRIM27 | GGAGGGCTTCAAGGAGCAA | GCTGCTCAAACTCCCAGACA |
| mTRIM28 | TGCTGCCCTGTCTACATTCG | ACTGGACAATCCACCATAGCG |
| mTRIM29 | AAGGCTTTCCCTCCCTCCT | CCGGTAGTGAGACAGCAGAG |
| mTRIM30a | CTCAGTTCCTGCCCTCACAG | AGGAGCAAACACAGGCAAGA |
| mTRIM31 | CTTTTCGGGCTCCAAGTTGC | TGTCTCATCCCGTCCTGAGT |
| mTRIM32 | GGGCGGTCAGCAGGAATC | GCATCCAGGTTCAGGTGAGA |
| mTRIM33 | ACACAAGAGATTAATTTGAAGGGTG | TGGCAAAGGAGTGAAGGTCC |
| mTRIM34a | GGAGGTGAGGGTCCAAAGATTC | GCTGGATGGCTAGGCAGATT |
| mTRIM35 | CTAGCAGAACAGACCGAGGC | GGCTCTTGTGTTTCATGAGGA |
| mTRIM36 | AGGGAGCAACCGCACTC | GGTGACATTAACCACGGAGTCT |
| mTRIM37 | GCCCCTGAGGAAGGAATGAG | ACTGGAATGTGAATCTTCATCGG |
| mTRIM38 | AGGCTGATCCCCTGAAGGTT | CAGGATCACGCTGACTTGGTG |
| mTRIM39 | TGGAGGGCAAGTGCTTACAG | TCCAGAGTCACATCCGCAAT |
| mTRIM40 | GAAGAACCGGCTCATCTGCT | GAACCTTGTCAGTGACCCTCA |
| mTRIM41 | GGCTGCAAGAGTGGACCTG | CCCACCTCTACCTCCCAGTAG |
| mTRIM43 | CTCAACTCCTTCCGAGTGAAGA | CCTTCAAATCCACCTCCCAGT |
| mTRIM44 | GGGCTGGATTTGAGCACCTA | CACCGGAATCTTTGCTTCTCAG |
| mTRIM45 | CCAGGCTCATAGGCGACAG | CTACACAGTCCCGACACACC |
| mTRIM46 | GAAGAGAGGGACCAAGACAAGC | CCCACCATGGCCTATGTACC |
| mTRIM47 | GGCTACAGAAACTCGGCTCA | CACGATGTAGGCAAACTTGAGG |
| mTRIM50 | CTACAGCCGCATGAAGGAAG | TGTTGTTCACCAGTTTGCCG |
| mTRIM52 | AATGGCCACCAGTACCATCG | GTCTGGATAGAGCTGCCGAC |
| mTRIM54 | GTGCCAGACCATTGAGGACA | CTTGAAGCAGTTCGCCCTTG |
| mTRIM56 | AGGGCATTTTCCCAACTAGCA | TTGCCAGCCAACACTACTCT |
| mTRIM58 | AGTGGGACTGATGAGTGGGT | AATGAAGCCTCGGGCAGTAG |
| mTRIM63 | ATGAGTGAGACACGCTCTGG | TGGGGAGCCCTATGCTAGTC |
| mTRIM65 | GCTCTGGCAGAATTATCGCAATC | AAATGGTGTGTCACCCGCTG |
| mTRIM66 | ATGAGTGAGACACGCTCTGG | TGGGGAGCCCTATGCTAGTC |
| mTRIM67 | TGGAGCCCAAAGTATCTGCG | GGGGTACAGGTGGCAATTTCA |
| mTRIM68 | TACTCCCGCCTTGTCGTTTC | GATGCATTGGCTGCCCAAAA |
| mTRIM69 | GATGCCGAGTGGGTCAGAAC | GCAGTGTAGCTCCGTGGTAA |
| mTRIM71 | GACCGCATTATGTTCACGCC | TGACAGTGAAAGAGGCCACC |
| mTRIM72 | AGGTAGTTACAGGATGGGGCT | CATGGTGAGCCTGGGAAGAG |
| mTRIM75 | CTGATGGCACATGTCGAGGT | ACAGAAGTTGTGTCCGCACT |
| mTRIM80 | TCGCCAACAGGAGTGATGAG | TTGGTGCCTAGCAAGGGTTC |
| rTRIM1 | GAACGAGTGGATTGGCAAGA | CTGGGTCATAGAAAGACAGCATG |
| rTRIM2 | CGTGGAAACTGAGGGATTGA | GATGATGGTCTGCCGAAGC |
| rTRIM3 | ATGGACAAGCAGTTTCTGGTATG | GGCTCTGAGGAGGGATGTAGTT |
| rTRIM5 | ACCGCCGACAAAAGACAGA | ACCGCCGACAAAAGACAGA |
| rTRIM6 | GGCGTGGATCTTGGGTGT | CTCATAGGCTCTGTACTCGTGCT |
| rTRIM7 | AATCCCCGCCTCATCTTGT | AGCCCACTTCCACCTCCC |
| rTRIM8 | CGCATCAGGGACACTCGG | CTTCCTCCTTCAGTTGGCTCA |
| rTRIM9 | GGCGTGGTTTGCGTTTG | TGCTGGATCAGGGTGGTTG |
| rTRIM10 | CCTAAGGGAACCAGTCACCAT | GCTCGATATTCTCCACCACATT |
| rTRIM11 | GCAGGGTGCAGGATGTGAA | GTGTCTGGGTCCAAGGTTATGT |
| rTRIM13 | CGATACCAGTCAGTGGGAGGA | GGGCCAAAGAATACAAGGAGA |
| rTRIM14 | ACCCTGTGCCCCTGTCTTT | CCTTCAGGCGAGTGTCCATT |
| rTRIM15 | AAAGGCTGAAGCGGGAGC | TGGGAGACCGTCGTGATGTA |
| rTRIM16 | AAGTCTGTTTTGGTGTCCGTGTC | GTGGGTCTTGATGCCGTTG |
| rTRIM17 | GGGAAGGGCAAGAAAGGG | TTCGTCAGTAGGCGGTTGG |
| rTRIM21 | GTTGATTCCCCAGACCTGACA | TTGAGATGATGAGGCACGAGTT |
| rTRIM23 | CGGCTGTGGTGAAGACTATACAT | CAAAGGAGAAGGCGAGGAAC |
| rTRIM24 | ACGACTCGGATGATGACTTTGT | GCTGCGGTTGGTTGCCTAA |
| rTRIM25 | TGCGGCCTCACATCGAC | TCTCCGTGCTCAGGACAAAAC |
| rTRIM26 | ACTGTTGTGCGTGATGTGCC | CCTCAGGGTGTTCAAGTGGTTC |
| rTRIM27 | GCTGTGGAAGGCTTCAAGGA | GCTGCTCAAACTCCCAAACAA |
| rTRIM28 | AGGGGAGGAATGGAGTTGC | TGGAGAATGTAGAGTCGGTAGCC |
| rTRIM29 | CTCCACCTCTGCCCACCTAC | GTCACCGCCGTTTTCCATA |
| rTRIM30 | TCCATCCCAGAGGAAGAACAG | TCGCTCACAAAGCCAGCA |
| rTRIM31 | TTCCACGATGTCACCTTGATAGA | GATTGCCCCTTCGCCTC |
| rTRIM32 | GGGCAACTATCGCATACAAGTG | GGGCAAGTCGGCTCCAA |
| rTRIM33 | CAGTTGGAAGATGCTGGCTCA | CCAGGTGATAACGCAGAGGGT |
| rTRIM34 | GCCACTTGCCCTGTCTGC | CCCTCTGGGGTTGGTCACTG |
| rTRIM35 | ACCTCACGAGCGTCATCAACC | TGGGAGCCCTTGGAGAAAA |
| rTRIM36 | GACAGCGGAAGTGAGGACG | TTCGGGTTCATTAGAAGAAGTAGG |
| rTRIM37 | TGCTGAGGTTTTCCGATGC | TGGAGTGGAGCACGACAATG |
| rTRIM39 | GCCGATGGACGATGCTACA | GTTCCCCTGGTTTCCTTTCTT |
| rTRIM40 | GAGGCTGTGAGCACCGACTG | GCTGAGAACCCCGGAGACC |
| rTRIM41 | GCAGGCAGGACTAGAACGG | CACCTATTGAAAGTCTCCTTGATGT |
| rTRIM42 | AGGGCAAGGCGGGTCTA | CTGCGGAGGCTGGGTTT |
| rTRIM44 | CCAGTCATTGGGGCTCACC | GCCTTCAGTCCACCAGAATCTTT |
| rTRIM45 | TGGCTGATATGCGGACTGG | CGGGCACTGTATTCAACCTTA |
| rTRIM46 | GTATGTGCTGCGTGTCCGTG | GCTGGTCCTTGCTGATGGC |
| rTRIM47 | CGCCACTCAGGAGCATCG | AGTACGGCGGGATTGTGC |
| rTRIM50 | ATCGCATTGGCGTCTACCTA | ATCGCATTGGCGTCTACCTA |
| rTRIM52 | TCATAGCCATAGAAGGCATCG | TCATAGCCATAGAAGGCATCG |
| rTRIM55 | GCTCAGTGATGGTATTGCTGTACT | GATGGCTTGGGTCATTTCG |
| rTRIM56 | GAGGAGTTATTGGCGGGTGT | GCAGGGATTTGAGGATTCGTT |
| rTRIM58 | TCACAAATGGGTCTTACATCTACAC | GGCAGCAACAGCAAAATCAA |
| rTRIM59 | GCACAATTTTGAGGACGAGTTAAC | TGAGTGGGATTCGCAGAGGT |
| rTRIM62 | GCACGACGGCAACCAGTA | GCACGACGGCAACCAGTA |
| rTRIM63 | TGCCAAGCCGCTCATCA | CCTCCTCATCTGTCCCAAAGTC |
| rTRIM65 | AGTTCAGCCCGCACCCT | AGCGCCCGCTTCTTGG |
| rTRIM66 | CGCCTGCCGCTTATGG | GCTGCCTCACGATGTAGTTGAT |
| rTRIM67 | AGGACATGATGCTGGGTAAAGA | TGTTGCCCGTTGATGAAGAA |
| rTRIM68 | AGAACCTGCCTGACAATCCTG | TCCAACCTCCACTTCCCAGT |
| rTRIM69 | GGCGAACATTCAGGCACG | TTGGAGATAAGCTGTCTGGGTAC |
| rTRIM71 | CACCTGCTCCGTGCCCATC | TGAGTGCCCGTGAGTCCTGT |
| rTRIM72 | TGGTGGTGTCCGCCTCTG | CTACCGCTACCGTCTTGTCG |
| hTRIM2 | TCAGAGCCCTTTCCTGTCCT | CCTGCACGCTGGTTTCCAA |
| hBAX | GGGGAGCAGCCCAGAGG | CGATCCTGGATGAAACCCTGA |
| mBAX | AAACTGGTGCTCAAGGCCC | AGCCACCCTGGTCTTGGAT |
| rBAX | AAACTGGTGCTCAAGGCCC | GGGTCCCGAAGTAGGAAAGG |
| hBCL2 | GGGGTCATGTGTGTGGAGAG | GTTCCACAAAGGCATCCCAG |
| mBCL2 | TGAGTACCTGAACCGGCATC | TATAGTTCCACAAAGGCATCCCAG |
| rBCL2 | TGTGTGTGGAGAGCGTCAAC | ATAGTTCCACAAAGGCATCCCAG |
| hBAD | CGAAGGGATGGGGGAGGA | GGCGAGGAAGTCCCTTCTTA |
| mBAD | AGGATCCAAATGGGAACCCC | GGAACATACTCTGGGCTGCT |
| rBAD | TCCCCAAGGCAGGAGGAAT | TGCTTTGGGGTTCCCATTTG |
| hBNIP3 | GCCATCGGATTGGGGATCTAT | GCCACCCCAGGATCTAACAG |
| hβ-actin | GCCGCCAGCTCACCAT | TCGTCGCCCACATAGGAATC |
| mβ-actin | GATATCGCTGCGCTGGTCG | CATTCCCACCATCACACCCT |
| rβ-actin | CCCGCGAGTACAACCTTCTTG | GTCATCCATGGCGAACTGGTG |
