# Supplementary material for: TRIM2 inhibits apoptosis by ubiquitinating BNIP3 to protect the intestine against ischemia-reperfusion injury in mice
Source: Commun Biol. 2025 Aug 29;8:1308. doi: 10.1038/s42003-025-08708-2 (PMC12397258; doi:10.1038/s42003-025-08708-2)
Supplement: Supplementary file 6 — Reporting Summary [file 42003_2025_8708_MOESM6_ESM.pdf]

Corresponding author(s): Yong Li

Last updated by author(s): May 19, 2025

Reporting Summary

Nature Portfolio wishes to improve the reproducibility of the work that we publish. This form provides structure for consistency and transparency in reporting. For further information on Nature Portfolio policies, see our Editorial Policies and the Editorial Policy Checklist.

Statistics

For all statistical analyses, confirm that the following items are present in the figure legend, table legend, main text, or Methods section.

n/a	Confirmed	
☐	☒	The exact sample size (*n*) for each experimental group/condition, given as a discrete number and unit of measurement
☐	☒	A statement on whether measurements were taken from distinct samples or whether the same sample was measured repeatedly
☐	☒	The statistical test(s) used AND whether they are one- or two-sided<br>*Only common tests should be described solely by name; describe more complex techniques in the Methods section.*
☒	☐	A description of all covariates tested
☒	☐	A description of any assumptions or corrections, such as tests of normality and adjustment for multiple comparisons
☒	☐	A full description of the statistical parameters including central tendency (e.g. means) or other basic estimates (e.g. regression coefficient) AND variation (e.g. standard deviation) or associated estimates of uncertainty (e.g. confidence intervals)
☒	☐	For null hypothesis testing, the test statistic (e.g. *F*, *t*, *r*) with confidence intervals, effect sizes, degrees of freedom and *P* value noted<br>*Give P values as exact values whenever suitable.*
☒	☐	For Bayesian analysis, information on the choice of priors and Markov chain Monte Carlo settings
☒	☐	For hierarchical and complex designs, identification of the appropriate level for tests and full reporting of outcomes
☒	☐	Estimates of effect sizes (e.g. Cohen's *d*, Pearson's *r*), indicating how they were calculated

Our web collection on statistics for biologists contains articles on many of the points above.

Software and code

Policy information about availability of computer code

Data collection	n/s
Data analysis	Figdraw, Graphpad Prism10, Image J

For manuscripts utilizing custom algorithms or software that are central to the research but not yet described in published literature, software must be made available to editors and reviewers. We strongly encourage code deposition in a community repository (e.g. GitHub). See the Nature Portfolio guidelines for submitting code & software for further information.

Data

Policy information about availability of data

All manuscripts must include a data availability statement. This statement should provide the following information, where applicable:

- Accession codes, unique identifiers, or web links for publicly available datasets
- A description of any restrictions on data availability
- For clinical datasets or third party data, please ensure that the statement adheres to our policy

n/s

Research involving human participants, their data, or biological material

Policy information about studies with human participants or human data. See also policy information about sex, gender (identity/presentation), and sexual orientation and race, ethnicity and racism.

Reporting on sex and gender	n/s
Reporting on race, ethnicity, or other socially relevant groupings	n/s
Population characteristics	n/s
Recruitment	n/s
Ethics oversight	n/s

Note that full information on the approval of the study protocol must also be provided in the manuscript.

Field-specific reporting

Please select the one below that is the best fit for your research. If you are not sure, read the appropriate sections before making your selection.

☒ Life sciences ☐ Behavioural & social sciences ☐ Ecological, evolutionary & environmental sciences

For a reference copy of the document with all sections, see nature.com/documents/nr-reporting-summary-flat.pdf

Life sciences study design

All studies must disclose on these points even when the disclosure is negative.

Sample size	Sample size is estimate on the basis of our previous publication without prior power analysis
Data exclusions	No data were excluded
Replication	The same experiment was performed three times with independent groups, and the same trend was observed.
Randomization	All mice were age and sex-matched and then randomized into the different groups.
Blinding	The investigators were not blinded to group allocation during experiments. Conclusions were made based on quantitative parameters and statistical significance of the data, and thus on experimental observations, independent of blinding.

Reporting for specific materials, systems and methods

We require information from authors about some types of materials, experimental systems and methods used in many studies. Here, indicate whether each material, system or method listed is relevant to your study. If you are not sure if a list item applies to your research, read the appropriate section before selecting a response.

Materials & experimental systems

n/a	Involved in the study	
☐	☒	Antibodies
☐	☒	Eukaryotic cell lines
☒	☐	Palaeontology and archaeology
☐	☒	Animals and other organisms
☒	☐	Clinical data
☒	☐	Dual use research of concern
☒	☐	Plants

Methods

n/a	Involved in the study	
☒	☐	ChIP-seq
☐	☒	Flow cytometry
☒	☐	MRI-based neuroimaging

Antibodies

Antibodies used	The antibodies used were anti-Trim2 (20356-1-AP and 67342-1-Ig), anti-Bax (50599-2-Ig), anti-Bcl-2 (12789-1-AP), anti-cleaved-Casp3 (19677-1-AP), anti-Bad (10435-1-AP), anti-β-actin (67735-1-Ig), anti-Flag (66008-4-Ig and 20543-1-AP), anti-GFP (66002-1-Ig and 50430-2-AP), and anti-HA (66006-2-Ig and 51064-2-AP) antibodies purchased from Proteintech (Wuhan, China). The Ubiquitin

(ab7780), K48 ubiquitin (ab140601), and K63 ubiquitin (ab179434) antibodies were purchased from Abcam. The Bnip3 antibody (44060S) was purchased from Cell Signaling Technology.

Validation

Validation statements on the manufacturer's website are noted.

Eukaryotic cell lines

Policy information about cell lines and Sex and Gender in Research

Cell line source(s)

Caco-2 cells were purchased from Wuhan Pricella Biotechnology Co., Ltd.,The HEK293T cells were obtained from ATCC, while the IEC-6 cells were sourced from Shanghai Anwei Biotechnology Co., Ltd., Primary intestinal epithelial cells (IECs) from mouse intestine were obtained from Meisencell Biological Co. Ltd (CTCC-D007-MIC, Zhejiang, China).

Authentication

Cell lines are purchased directly and carefully preserved in the laboratory. Cell lines were examined for their morphology by microscopy.

Mycoplasma contamination

All cells tested negative for mycoplasma.

Commonly misidentified lines
(See ICLAC register)

No misidentified cell lines.

Animals and other research organisms

Policy information about studies involving animals; ARRIVE guidelines recommended for reporting animal research, and Sex and Gender in Research

Laboratory animals

Male C57BL/6 mice (8 weeks old, specific pathogen-free)

Wild animals

No animals were observed or captured in the field.

Reporting on sex

All the experimental animals used in this study were male.

Field-collected samples

In this study, the intestine tissue of mice was taken in the SPF animal laboratory.

Ethics oversight

All procedures involving mice were conducted in accordance with protocols approved by the Ethics Committee of The First Affiliated Hospital of Nanchang University (Approval No. CDYFY-IACUC-202407QR215) and in compliance with the Guide for the Care and Use of Laboratory Animals (NIH publication No. 86-23, revised 2011).

Note that full information on the approval of the study protocol must also be provided in the manuscript.

Plants

Seed stocks

n/s

Novel plant genotypes

n/s

Authentication

n/s

Flow Cytometry

Plots

Confirm that:

☒ The axis labels state the marker and fluorochrome used (e.g. CD4-FITC).

☒ The axis scales are clearly visible. Include numbers along axes only for bottom left plot of group (a 'group' is an analysis of identical markers).

☒ All plots are contour plots with outliers or pseudocolor plots.

☒ A numerical value for number of cells or percentage (with statistics) is provided.

Methodology

Sample preparation

IEC-6 and Caco-2 cells were cultured in 6-well plates (1×10^6 cells/well) for a period of 24 hours at 37°C and 5% CO_2. For H/R

Sample preparation	treatment, cells were incubated in DMEM without FBS, and treated with hypoxia under 5% CO2 and 1% O2 balanced with 94% N2 gas for 24 h (IEC-6 cells) or 12 h (Caco-2). Following this, the cells were reoxygenated by receiving fresh medium containing 10% FBS and were maintained under normoxia conditions for 4 h. Cells were harvested with trypsin/EDTA solution and suspended in PBS.
Instrument	NovoCyte D3000 (Agilent)
Software	FlowJo v10 software (FlowJo; BD Biosciences)
Cell population abundance	The purity of the samples was not determined.
Gating strategy	A gate was set using forward scatter (FSC) vs. side scatter (SSC) plot to select the cell population of interest. The apoptotic level of cells subjected to different treatments was assessed using the Annexin V-Alexa Fluor 647/PI Apoptosis Detection Kit (40304ES60, YEASEN, Shanghai, China), in accordance with the manufacturer's instructions.

☒ Tick this box to confirm that a figure exemplifying the gating strategy is provided in the Supplementary Information.
